# Cooperative Sulfur Transformations at a Dinickel Site: A Metal Bridging Sulfur Radical and Its H-Atom Abstraction Thermochemistry

**DOI:** 10.1021/jacs.4c05113

**Published:** 2024-08-07

**Authors:** Valeria Tagliavini, Peng-Cheng Duan, Sayanti Chatterjee, Eleonora Ferretti, Sebastian Dechert, Serhiy Demeshko, Liqun Kang, Sergey Peredkov, Serena DeBeer, Franc Meyer

**Affiliations:** † Institute of Inorganic Chemistry, University of Göttingen, Tammannstr. 4, D-37077 Göttingen, Germany; ‡ Max Planck Institute for Chemical Energy Conversion, Stiftstrasse 34-36, D-45470 Mülheim an der Ruhr, Germany; § Department of Chemistry, Indian Institute of Technology Roorkee, Roorkee, Uttarakhand 247667, India; ∥ International Center for Advanced Studies of Energy Conversion (ICASEC), University of Göttingen, Tammannstr. 6, D-37077 Göttingen, Germany

## Abstract

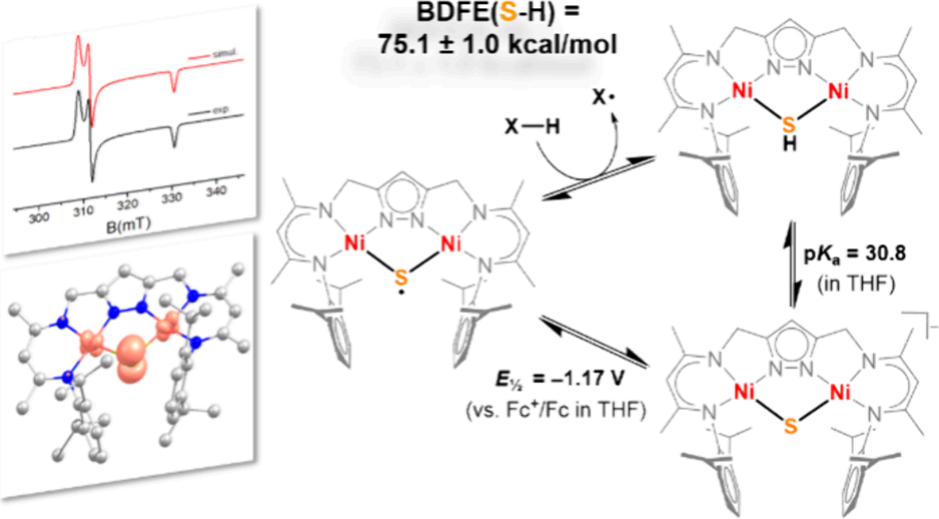

Starting from the dinickel(II) dihydride complex [ML(Ni–H)_2_] (**1**^**M**^), where L^3–^ is a bis(tridentate) pyrazolate-bridged bis(β-diketiminato) ligand and M^+^ is Na^+^ or K^+^, a series of complexes [KLNi_**2**_(S_2_)] (**2**^**K**^), [MLNi_2_S] (**3**^**M**^), [LNi_2_(SMe)] (**4**), and [LNi_2_(SH)] (**5**) has been prepared. The μ-sulfido complexes **3**^**M**^ can be reversibly oxidized at *E*_1/2_ = −1.17 V (in THF; vs Fc^+^/Fc) to give [LNi_2_(S^•^)] (**6**) featuring a bridging S-radical. **6** has been comprehensively characterized, including by X-ray diffraction, SQUID magnetometry, EPR and XAS/XES spectroscopies, and DFT calculations. The p*K*_a_ of the μ-hydrosulfido complex **5** in THF is 30.8 ± 0.4, which defines a S–H bond dissociation free energy (BDFE) of 75.1 ± 1.0 kcal mol^–1^. **6** reacts with H atom donors such as TEMPO-H and xanthene to give **5**, while **5** reacts with 2,4,6-tri(*tert*-butyl)phenoxy radical in a reverse H atom transfer to generate **6**. These findings provide the first full characterization of a genuine M–(μ-S^•–^)–M complex and provide insights into its proton-coupled electron transfer (PCET) reactivity, which is of interest in view of the prominence of M–(μ-SH/μ-S)–M units in biological systems and heterogeneous catalysis.

## Introduction

Cofactors containing transition metals and S-derived ligands such as sulfido (S^2–^), hydrosulfido (HS^–^) or thiolato (RS^–^) ligands, play many important roles in biological redox transformations, and metal sulfides are intimately related to the evolution of life on earth.^1^ Prominent examples include the ubiquitous Fe/S clusters^2^ or some enzymes of ancient origin such as Ni,Fe-containing carbon monoxide dehydrogenases (CODHs) and acetyl-CoA synthases (ACSs)^3^ as well as Mo/Cu CODH.^4^ On the other hand, transition metal sulfides such as Ni/Co-promoted Mo-based catalysts are widely used in refineries for hydrodesulfurization (HDS) of petroleum feedstock.^5^ This has triggered significant interest in the study of metal complexes with HS^–^ and various S_n_^x–^ ligands^6^ as model systems for the metalloprotein active sites^7−9^ or the surface sites of industrial catalysts.^10^

**Figure 1:**
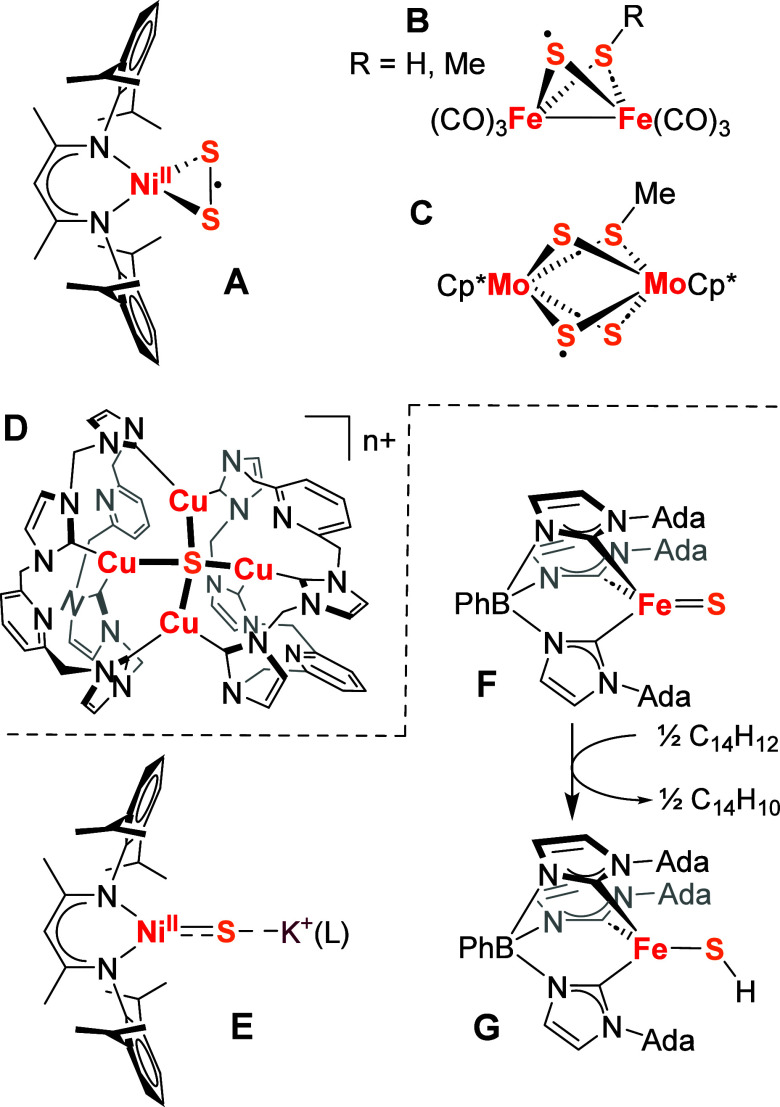
Selected examples of known mid and late transition complexes with S_2_^•–^ (A) or μ–S^•–^ (B, C) ligands (top), or with {Cu_4_(μ_4_-S)} (D), {Ni = S} (E) or terminal {Fe = S} (F) cores and HAA reactivity of the latter.

In transition metal complexes with S-donor ligands the high covalency of M–S bonds and the rich redox activity of sulfur can give rise to classical metal-based (ligand innocent) or ligand based redox behavior. As an example, the SOMO of the {Cu_4_S} cluster **D** (Figure 1) in its oxidized form (n = 3; *S* = 1/2), which is a model of the Cu_Z_ active site of N_2_O reductase (N_2_OR) in the 1-hole state, has about 39% contribution from the 3p_*z*_ orbital of the μ_4_-S.^9b^ X-ray absorption spectroscopy (XAS) has been a valuable tool for assessing ground state orbital composition of M–S bonding as well as metal–ligand bond covalency.^11^

Metal–thiolate (cysteinate) entities are particularly widespread in nature, and much work has been devoted to assessing the redox noninnocence of thiolato ligands and to identifying metal stabilized thiyl radicals.^12,13^ In contrast, there is limited information about “naked” sulfur radicals stabilized by metal coordination, despite the potential relevance of such sites. Sulfur radical anions such as S_3_^•–^ (but also disulfur S_2_^•–^ and tetrasulfur S_4_^•–^ radicals) give rise to the intense blue color of lapis lazuli,^14^ and they have been proposed as relevant species in photoredox cycles using solutions of K_2_S_*x*_ as photoredox catalyst.^15^ Some metal complexes with either end-on or side-on bound supersulfido (S_2_^•–^) ligands are known^16^; a prominent example is the paramagnetic (S = 1/2) complex **A** (Figure 1) reported by Driess et al., which dimerizes in solid state.^17^

Recently, Gong et al. reported the preparation of radical complexes M(O)(S)F_2_ (M = V, Nb, Ta) from the reactions of laser-ablated metal atoms and SOF_2_ in cryogenic matrixes, and from IR spectroscopy and density functional theory (DFT) calculations they concluded that the unpaired electron is located in a 3p orbital of the terminally bound sulfur.^18^ Terminal metal sulfides, whose potential radical character is often inferred only from their reactivity, are generally difficult to isolate, partly because the pronounced sulfur catenation tendency.^19^ Early evidence for terminal {Ni = S} intermediates was provided by Jones et al.,^20^ and a masked terminal {Ni = S} complex **E** (Figure 1) could be characterized by X-ray diffraction in 2015 by Hayton et al.^21^ The latter was shown to be highly nucleophilic and to react with various substrates such as CS_2_, NO, CO, and N_2_O.^22^ Most recently the terminal high-spin (*S* = 5/2) {Fe^III^=S} complex **F** could be structurally characterized and was shown to react with dihydroanthracene (DHA) via H atom abstraction (HAA) to give hydrosulfide complex **G**(23,24); the gas phase bond dissociation free energy (BDFE) of the S–H bond in **G** was estimated to be 70 kcal mol^–1^ based on DFT calculations. While mid and late transition metal oxo complexes {M = O} often react via HAA, this reactivity pattern is uncommon for metal sulfides that usually tend to couple forming S–S bonds instead. This has been rationalized in terms of the difference in E–H bond strengths (e.g., BDFE(S–H) = 79.2 kcal mol^–1^ for MeSH versus BDFE(O–H) = 96.4 kcal mol^–1^ for MeOH in the gas phase).^19,25^

In view of the prominence of metal-bridging μ-SH and μ-S sites for redox processes in biological systems and heterogeneous catalysis, and in view of the important role of sulfide ligands as proton relays (e.g., in the [FeMo] cofactor of nitrogenase),^26^ information about the S–H bond strength and proton coupled electron transfer (PCET) thermochemistry for M–S(H)–M sites would be very valuable, as would be the characterization of genuine metal stabilized sulfur radical ligands S^•–^. Of note, it has recently been reported that S-mediated C–H bond cleavage by sulfur adatoms on transition metal sulfides likely is the rate-determining step for the H_2_S reforming of methane.^27^ However, experimentally determined BDFE(S–H) values for well-defined complexes in solution are scarce and only available for two systems studied by Franz et al.: Complex (OC)_3_Fe(μ-SCH_3_)(μ-SH)Fe(CO)_3_ has a BDFE(S–H) of 69.4 ± 1.7 kcal mol^–1^, and according to DFT the spin density in the corresponding radical complex **B** (Figure 1) is largely shared between the two Fe centers and the μ-S bridge.^28^ A series of Cp*_2_Mo_2_S_4_(Me_n_H_m_) complexes, which serve as models for heterogeneous molybdenum sulfide catalysts, feature BDFE(S–H) values in the range 43–68 kcal mol^–1^ depending on the redox state.^29^ In both cases, however, the μ-S^•–^ species (**B**, **C**) rapidly dimerize via S–S bond formation, and only **C** could be characterized by EPR spectroscopy of the radical-dimer equilibrium mixture.^30^

**Scheme 1:**
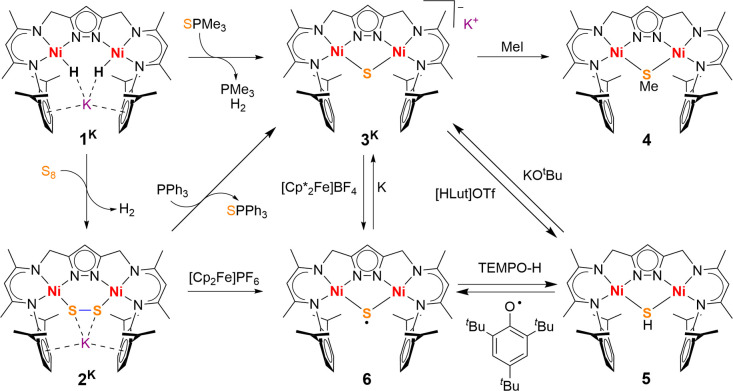
Overview of the Reactions and Complexes Studied in This Work

The present work aimed at the first full characterization of a genuine M–(μ-S^•–^)–M transition metal complex and the quantitative evaluation of its PCET thermochemistry. To that end we used a highly preorganized dinickel scaffold {LNi_2_} based on a trianionic pyrazolato-bridged bis(β-diketiminato) ligand platform, L^3–^.^31^ The dinickel(II) dihydride complexes [KLNi_**2**_H_2_] and [NaLNi_**2**_H_2_] (**1**^**K**^, **1**^**Na**^; the former is depicted in Scheme 1) can be described as masked dinickel(I) synthons^32^ as they were shown to rapidly eliminate H_2_ in the presence of various substrates such as HC≡CPh, O_2_, NO, PhNO, etc.,^33^ giving product complexes with the twice reduced substrate spanning the two Ni^II^ ions. Subsequent redox transformations centered on the activated substrate held within the dinickel(II) pocket were found to be facile in many cases, such as the interconversion of peroxido {Ni^II^–(O_2_^2–^)–Ni^II^} and superoxido {Ni^II^–(O_2_^•–^)–Ni^II^} species.^33a^ Cooperative bimetallic activation of S_8_ by [KLNi_**2**_H_2_] now allowed for the isolation and comprehensive characterization of a family of dinickel(II) complexes with different μ-(H)S_n_^x–^ ligands in the bimetallic cleft, including the target sulfur radical complex (Scheme 1).

## Results and Discussion

### μ_1,2_-Disulfido and μ-Sulfido Complexes **2**^**K**^ and **3^K^/3^Na^**

**Figure 2:**
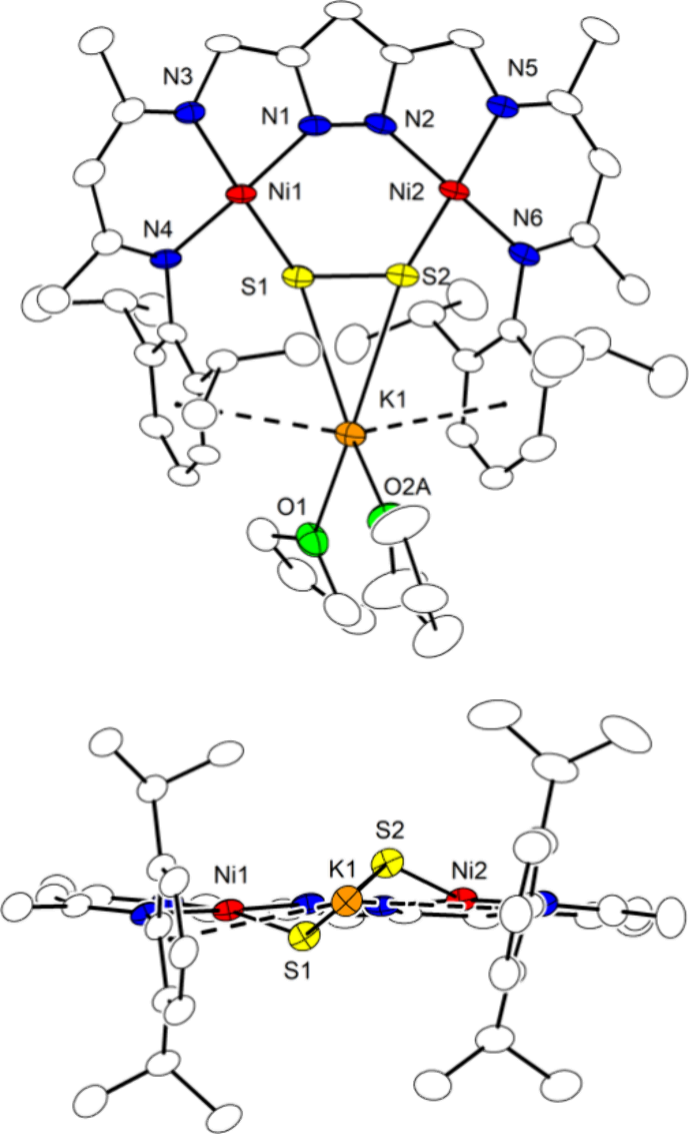
Views of the molecular structure of **2**^**K**^ (30% probability thermal ellipsoids). Hydrogen atoms are omitted for clarity; coordinating THF molecules have been removed for clarity in the bottom view.

Treatment of **1**^**K**^ with elemental sulfur in THF at rt led to an immediate change in color from orange to blood red, and gas evolution was observed. The reaction was also performed using the deuterium isotopologue of **1**^**K**^, [KLNi_**2**_D_2_] (**1**^**K-D2**^), in a J. Young NMR tube, and the release of D_2_ was confirmed by ^2^H NMR spectroscopy (Figure S1). The reaction thus follows the scenario previously observed for **1**^**K**^ (or **1**^**K-D2**^), i.e., via the reductive elimination of H_2_ (or D_2_, respectively) and 2e^–^ reductive binding of the added substrate. Red plate-like crystals of the product complex [KLNi_**2**_(S_2_)] (**2**^**K**^) suitable for X-ray diffraction were obtained in 80% yield by layering hexanes on a solution of **2**^**K**^ in THF at −30 °C. The molecular structure of **2**^**K**^ in solid state is displayed in Figure 2, and selected metric parameters are compiled in Table 1. The nickel ions are found four-coordinate in square planar environment within the two tridentate binding sites of L^3–^ as anticipated (sum of angles around Ni1 and Ni2 is 360.12° and 361.09°, respectively), with a disulfido unit in a *cis*-μ-1,2 bridging mode within the bimetallic pocket. The K^+^ is sandwiched between the flanking aryl groups of the β-diketiminato ligand parts with distances to the aryl ring centroids of 3.357(2) and 3.259(2) Å, indicative of cation-π interactions.^34^ Distances K1–S1 and K1–S2 are also quite short (3.103(2) and 3.157(2) Å, respectively), suggesting that the K^+^ contributes significantly to the stabilization of the disulfido unit in the bimetallic pocket. A similar situation was previously found for the dihydride complex **1**^**K**^(31) and the related μ-1,2-peroxido bridged dinickel(II) complex [KLNi_**2**_(O_2_)].^33a^ In the latter case, however, distances between K^+^ and the centroids of the aryl rings are even shorter (2.840(1)/2.830(1) Å), likely because of the much smaller Ni···Ni separation of 3.880(1) Å in [KLNi_**2**_(O_2_)]^33a^ compared to 4.290(1) Å in **2**^**K**^, caused by the size difference of the disulfido versus peroxido units within the pocket (cf. *d*(Ni···Ni) = 4.161(1) Å in the dihydride starting complex **1**^**K**^).^31^ Furthermore, shorter K–O distances of 2.515(3)/2.545(3) Å in [KLNi_**2**_(O_2_)] lead to the K^+^ being buried within the cleft of the two aryl groups, while it is more exposed and carries two additional THF ligands in **2**^**K**^.

**Table 1:** Selected Distances [Å] and Angles [°]

	**2^K^**	**3**^**K**^	**3**^Na^*	**4**	**5**	**6**
Ni–N	1.904(5)–1.931(6)	1.816(2)–1.932(2)	1.812(6)–1.927(6)	1.818(2)–1.899(2)	1.8272(19)–1.9031(19)	1.825(2)–1.898(2)
Ni–S	2.1599(19)/2.1672(19)	2.2404(7)/2.2435(7)	2.231(2)–2.2489(19)	2.2635(8)/2.2810(8)	2.2665(7)/2.2765(6)	2.2778(7)/2.2889(7)
Ni···Ni	4.2902(13)	3.6515(5)	3.6262(13)/3.6227(13)	3.6488(4)	3.7126(5)	3.7047(5)
S–S	2.1599(19)					
Ni–S–Ni	107.73(3) (cent. S–S)	109.05(3)	107.91(7)/108.33(8)	106.81(3)	109.62(2)	108.44(3)
Ni–S_2_–Ni	81.1(1)					
τ_4_	0.09/0.13	0.11/0.12	0.12–0.15	0.11/0.17	0.13/0.16	0.12/0.15

* The asymmetric unit contains two crystallographically independent molecules.

**Figure 3:**
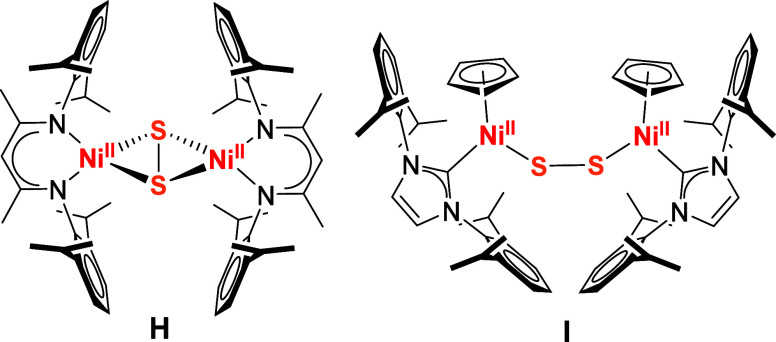
Selected examples of previously reported μ-disulfido dinickel(II) complexes.^17,39^

The front view of **2**^**K**^ (Figure 2 bottom) illustrates the large Ni1–S1–S2–Ni2 torsion angle of 81.1(1)°, similar to the Ni–O–O–Ni torsion angle of 81.4(3)° in [KLNi_**2**_(O_2_)]^33a^ and close to the C–S–S–C equilibrium angle of approximately 90° for organic disulfides.^35^ While disulfido-bridged dinuclear complexes with a M–S–S–M motif are well-known,^36^ few of them are based on dinucleating ligand scaffolds. The S–S distance of 2.036(3) Å in **2**^**K**^ is similar to the one observed in the only other crystallographically characterized dinickel complex with a Ni_2_(μ_1,2_-S_2_) motif **I** (2.0476(1) (Figure 3)^37^ but significantly shorter than values found for dinuclear, butterfly type disulfido dinickel(II) complexes with a Ni_2_(μ–η^2^,η^2^-S_2_) core such as **H** (2.177–2.297 Å)^38−40^ reflecting a less activated S–S bond in the *end-on* S_2_^2–^ bridge owing to a reduced back-donation by the Ni^II^ centers. It should be noted that polarization by side-on association of the K^+^ cation likely has a significant effect on the disulfido unit in **2**^**K**^, since the peroxido O–O bond in [KLNi_**2**_(O_2_)] was found to be lengthened compared to the one in [K(DB18C6)][LNi_**2**_(O_2_)] where the cation [K(DB18C6)]^+^ is separated from the complex anion (DB18C6 is dibenzo[18]-crown-6).^33a^

Complex **2**^**K**^ is diamagnetic in accordance with *S* = 0 Ni^II^(d^8^) ions, and it gives rise to sharp signals in the ^1^H and ^13^C NMR spectra in the common chemical shift range for C_2v_ symmetric complexes of the ligand L^3–^, evidencing that toggling of the S–S unit within the bimetallic pocket is rapid on the NMR time scale (Figures S1–S4). The UV–vis spectrum of **2**^**K**^ displays two intense bands centered at λ_max_ = 472 and 529 nm (ε ≈ 5000/4200 M^–1^ cm^–1^), which are tentatively assigned as the disulfide π*_σ_ → Ni^II^(d_*x*^2^__–*y*^2^_) and π*_ν_ → Ni^II^(d_*x*^2^__–*y*^2^_) charge-transfer (CT) transitions, respectively, from comparison with the absorption spectra of related disulfido complexes with Cu_2_(μ–η^2^:η^2^-S_2_) and Ni_2_(μ–η^2^:η^2^-S_2_) cores.^38–41^

**Figure 4:**
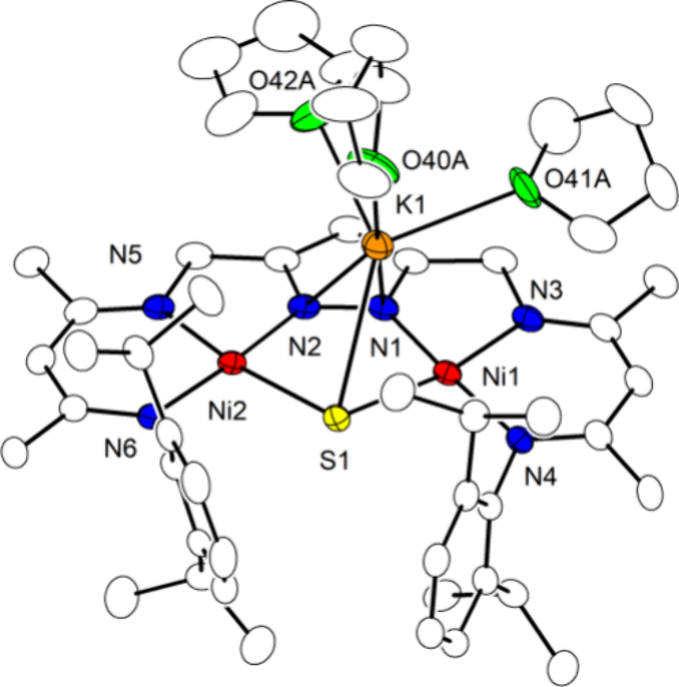
Plot (30% probability thermal ellipsoids) of the molecular structure of **3**^**K**^ (hydrogen atoms omitted for clarity).

The reaction of **2**^**K**^ with one equivalent of PPh_3_ in THF-*d*_8_ was followed via ^1^H and ^31^P NMR, showing gradual formation of SPPh_3_ and a new complex **3**^**K**^ over 2 days (Figures S9 and S10). Alternatively, **3**^**K**^ can be accessed directly via the reaction of dihydride complex **1**^**K**^ with SPMe_3_ at room temperature over 2 days, along with the formation of PMe_3_ (Scheme 1). The identity of **3**^**K**^ as the sulfido bridged dinickel(II) complex [KLNi_2_S] was confirmed by negative ion electrospray ionization (ESI) mass spectrometry of the reaction mixture, which shows a dominant signal at *m*/*z* = 753.32 characteristic for the anion [LNi_2_S]^−^ (Figure S17). Red crystals suitable for X-ray diffraction were obtained by hexane layering on a solution of **3**^**K**^ in THF; the molecular structure of **3**^**K**^ is shown in Figure 4.

The basic dinickel(II) core with both metal ions in square planar environment (sum of bond angles 360.07°/360.02°) is retained in **3**^**K**^, but a one-atom μ-S^2–^ is found in the pocket, leading to a much contracted Ni···Ni separation of 3.6515(5) Å. The quite acute Ni1–S–Ni2 angle of 109.05(3)° is in line with the Ni–S bonds mainly involving 3p orbitals on sulfur. The K^+^ in this case is located above the plane defined by the central five-membered {Ni_2_N_2_S} ring and is additionally ligated by three THF molecules. The Ni–S distances in complex **3**^**K**^ of 2.2404(7) Å and 2.2435(7) Å are significantly longer than the pseudoterminal Ni–S bond in **E** (2.064–2.084 Å) or the Ni–S bonds in known dinickel complexes with a μ-sulfido ligand such as [{PhB(CH_2_S*t*Bu)_3_}Ni]_2_(μ-S),^38^ [{L^tBu^Ni}_2_(μ-S)]^42^ and [{(IPr)Ni}_2_(μ-S)_2_] (IPr = 1,3-bis(2,6-diisopropylphenyl)imidazol-2-ylidene)^39^ that display *d*(Ni–S) in the range 2.06–2.10 Å. This is likely due to the geometric constraints imposed by the pyrazolato-based dinucleating ligand in **3**^**K**^.

Reacting the Na^+^ analogue of **1**^**K**^, [NaLNi_2_H_2_] (**1**^**Na**^),^31^ with SPMe_3_ gave the complex [NaLNi_2_S] (**3**^**Na**^) that could also be crystallized. Its molecular structure is very similar to the one of **3**^**K**^ (Table 1), with the Na^+^ similarly sitting on top of the {Ni_2_N_2_S} pentagon though closer to the Ni centers and only bound to two THF solvent molecules (Figure S51).

### Alkylation and Protonation of the μ-Sulfido Complex

**Figure 5:**
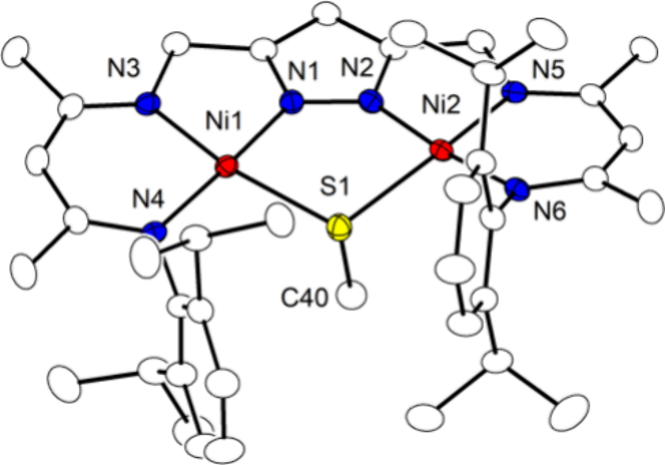
Plot (30% probability thermal ellipsoids) of the molecular structure of **4** (hydrogen atoms omitted for clarity).

Nucleophilicity of the bridging sulfide in **3**^**K**^ or **3**^**Na**^ was evidenced by the reaction with CH_3_OTs (TsO^–^ is tosylate) that leads to a rapid color change from red to olive green and formation of the μ-methylthiolato complex [LNi_2_(SMe)] (**4**). Green single crystals suitable for X-ray diffraction were obtained by layering hexanes on a solution of **4** in THF at low temperatures; the molecular structure is shown in Figure 5. The Ni···Ni separation remains essentially unchanged (3.6515(5) Å in **3**^**K**^ vs 3.6488(4) Å in **4**) while the Ni–S bonds become slightly longer concomitant with the Ni1–S1–Ni2 angle becoming even more acute (from 109.05(3)° in **3**^**K**^ to 106.81(3)° in **4**). The S-bound methyl group is oriented almost perpendicular to the plane defined by the central {Ni_2_N_2_S} ring. Bond angles around S close to 90° (Ni–S–C 92.74(11)/97.80(11)°) again indicate that the bonds mainly comprise sulfur p orbitals. The complex is diamagnetic and the SMe group appears as a singlet at 2.33 ppm in the ^1^H NMR spectrum. However, C_2v_ symmetry of **4** on the NMR time scale indicates rapid inversion at the bridging S atom with the methyl group swinging to both sides of the complex in solution.

**Figure 6:**
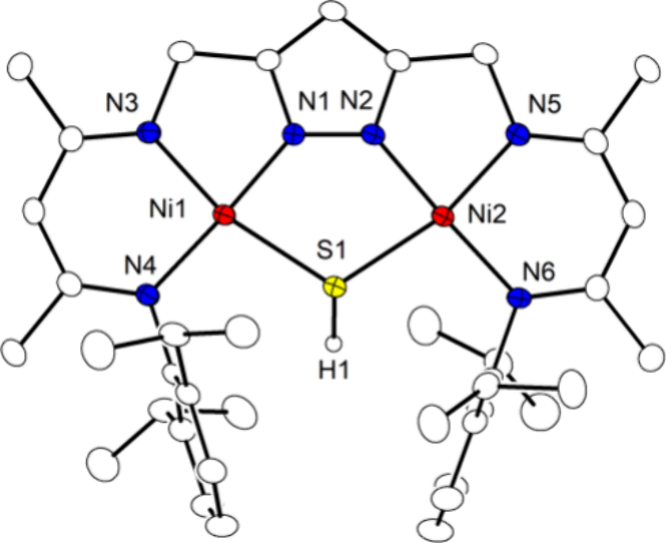
Plot (30% probability thermal ellipsoids) of the molecular structure of **5** (most hydrogen atoms omitted for clarity).

Treatment of **3**^**K**^ or **3**^**Na**^ with the relatively strong acid [HLut]OTf (p*K*_a_ = 9.5 in THF)^43^ produced the μ-hydrosulfido complex **5**, which is stable even when an excess of the acid is present. The characteristic CT absorption bands of **3**^**Na**^ at 458 and 373 nm decrease, while absorptions at 375 and 620 nm increase, with clean isosbestic points at 361, 388, and 574 nm (Figure S20). Crystals suitable for X-ray diffraction were obtained by layering hexanes on a solution of **5** in THF at room temperature; the molecular structure is shown in Figure 6. The {LNi_2_} core found in **3**^**K**^ and **4** is retained in **5** with a slightly elongated Ni···Ni separation of 3.7126(5) Å but very similar Ni–S bond lengths (2.2665(7)/2.2765(6) Å in **5**, 2.2635(8)/2.2810(8) Å in **4**) at. The SH proton could be found in the Fourier difference map and is located 1.21(3) Å from S1. In the IR spectrum the S–H stretching vibration is observed at 2508 cm^–1^ (Figure S36) similar to previously reported hydrosulfido complexes.^44^

**5** is diamagnetic as expected, and its ^1^H NMR spectrum displays the characteristic pattern of a C_2v_ symmetric complex of the pyrazolato-based ligand L^3–^. The SH proton resonates in the typical chemical shift range^44^ at relatively high field, δ = −3.49 (CDCl_3_) or −3.61 (THF) ppm (Figure S32). The positive ion ESI mass spectrum of a freshly prepared THF solution of **5** shows a major signal at *m*/*z* = 755.5 characteristic of the ion [**5**+H]^+^.

**Figure 7:**
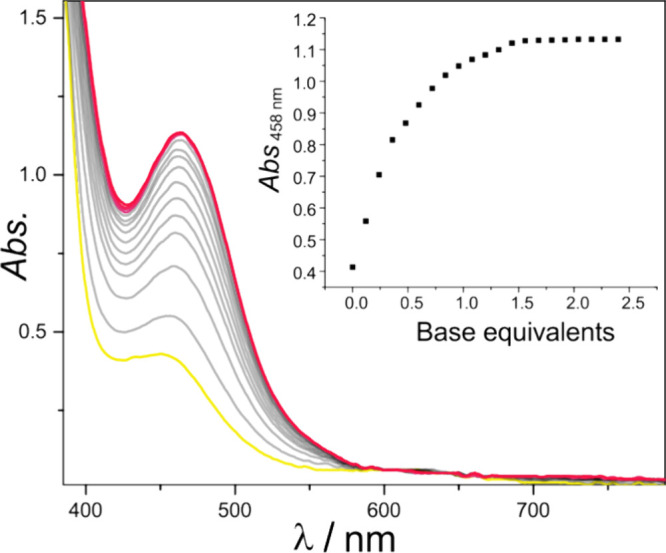
Titration of complex **5** (yellow) with the phosphonium ylide MeOCH = P(4-OMe-C_6_H_4_)_3_ at rt in THF, monitored by UV–vis spectroscopy. The inset shows the change of absorption at 458 nm vs equivalents of base added.

In order to determine the p*K*_a_ of the μ-SH unit, **3**^**Na**^ was treated with a variety of acids of different strength and the conversion to **5** was followed by UV–vis spectroscopy. One equivalent of either benzoic acid (p*K*_a_ = 25.1 in THF)^45^ or phenol (p*K*_a_ = 29 in THF)^46^ is sufficient to fully convert **3**^**Na**^ into **5** (Figures S20 and S21). On the other hand, back-titration of **5** with one equivalent of the phosphazene base P_4_-*t*Bu (p*K*_a_ = 33.9 in THF)^47^ gives **3**, which allows to bracket the p*K*_a_ of **5** in between 30 and 33 (Figure S23); addition of KO*t*Bu to **5** gives **3**^**K**^. Titration of **5** with the phosphonium ylide (MeOCH = P(4-OMe-C_6_H_4_)_3_ (p*K*_a_ = 31.7 in THF)^47^ established a proper equilibrium (Figure 7) and allowed to determine p*K*_a_(**5**) = 30.8 ± 0.4 (see SI for details).

### μ-Sulfido Radical Complex **6**

**Figure 8:**
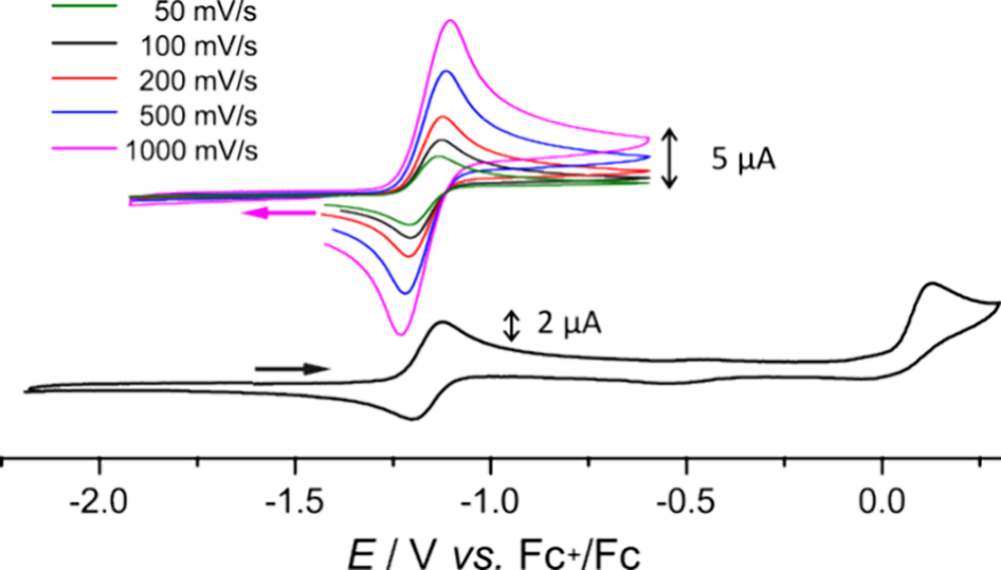
CV of **3**^**K**^ in THF at rt with NBu_4_PF_6_ as supporting electrolyte (0.1 M) in the range from −2.2 V to +0.3 V at scan 100 mV/s; the inset shows the reversible process at *E*_1/2_ = −1.17 V at different scan rates; potentials plotted versus Fc^+^/Fc.

Complexes **3**^**K**^/**3**^**Na**^ in THF show a well-behaved reversible oxidation at rather low potential *E*_1/2_ = −1.17 V vs the Fc^+^/Fc couple (Figure 8 and Figure S25), followed by an irreversible oxidation at *E*_a_^p^ ≈ 0.1 V (at scan rate 100 mV s^–1^). The reversible process at *E*_1/2_ = −1.17 V is independent of the alkali cation (K^+^ or Na^+^), and it also remains unperturbed if the crown ether 18-crown-6 is added to the solution of **3**^**K**^ prior to the CV measurement (Figure 8 and Figure S26). This suggests that **3**^**K**^/**3**^**Na**^ are solvent separated ion pairs in THF solution.

**Figure 9:**
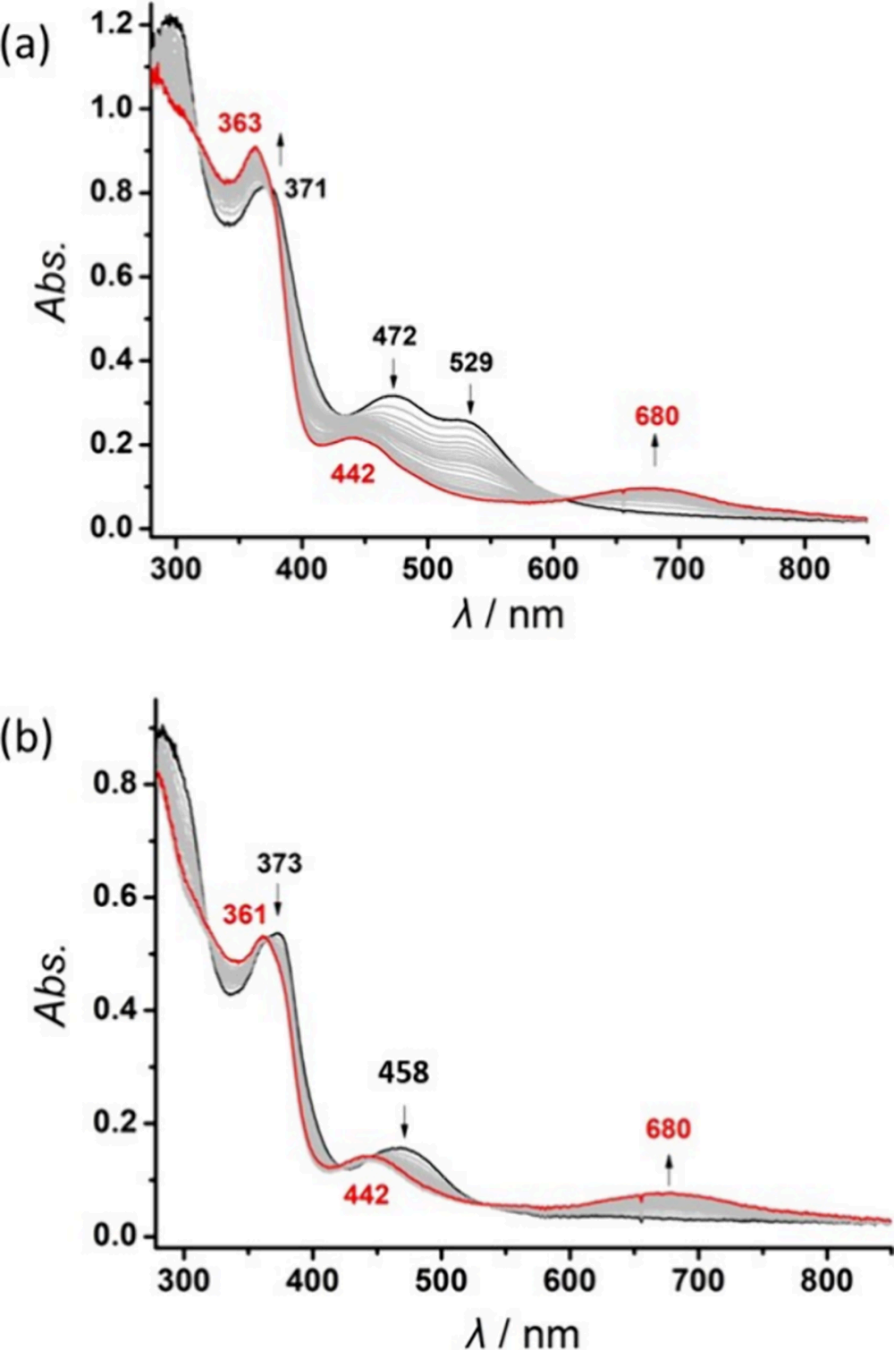
UV–vis SEC showing the oxidation of (a) **2**^**K**^ (black) or (b) **3**^**K**^ (black) to **6** (a and b, red) in THF solution containing 0.1 M [^n^Bu_4_N]PF_6_ (at −1.5 V vs Ag wire).

UV–vis spectro-electrochemistry (UV–vis SEC) of **3**^**K**^ shows the decrease of the characteristic absorption bands of **3**^**K**^ at 373 and 458 nm and the clean formation of a new species **6** with blue-shifted λ_max_ = 362, 442 nm and a new broad band at 680 nm (Figure 9b). Interestingly, the same species is also formed upon electrochemical oxidation of **2**^**K**^ (Figure 9a), suggesting that the disulfide unit of **2**^**K**^ loses one S atom upon oxidation. Indeed, the CV of **2**^**K**^ shows that **2**^**K**^ is irreversibly oxidized at *E*_a_^p^ ≈ −0.97 V and that a cathodic wave at *E*_c_^p^ ≈ −1.28 V typical for the reduction of formed **6** appears in the reverse scan (Figure S8). Desulfurization of a dinickel(II) disulfide complex upon oxidation has previously been observed by Riordan et al., who isolated in low yields the μ-sulfido complex [(PhTt^tBu^)Ni]_2_(μ-S) after reaction of [(PhTt^tBu^)Ni]_2_(μ–η^2^,η^2^-S_2_) with O_2_.^38^

Chemical oxidation of **3**^**K**^ in THF at −35 °C with decamethylferrocenium tetrafluoroborate, [Cp*_2_Fe]BF_4_ (*E*_1/2_ = −0.45 V in THF; see Figure S26),^48^ resulted in a rapid color change from red to emerald green, and green single crystals of the product **6** could be isolated in good yields (>50%) from layering hexanes onto solutions of the crude product in THF or toluene. Full conversion after addition of 1 equiv of [Cp*_2_Fe]BF_4_ (monitored by UV–vis spectroscopy) confirms the 1e^–^ nature of the redox process at *E*_1/2_ = −1.17 V.

**Figure 10:**
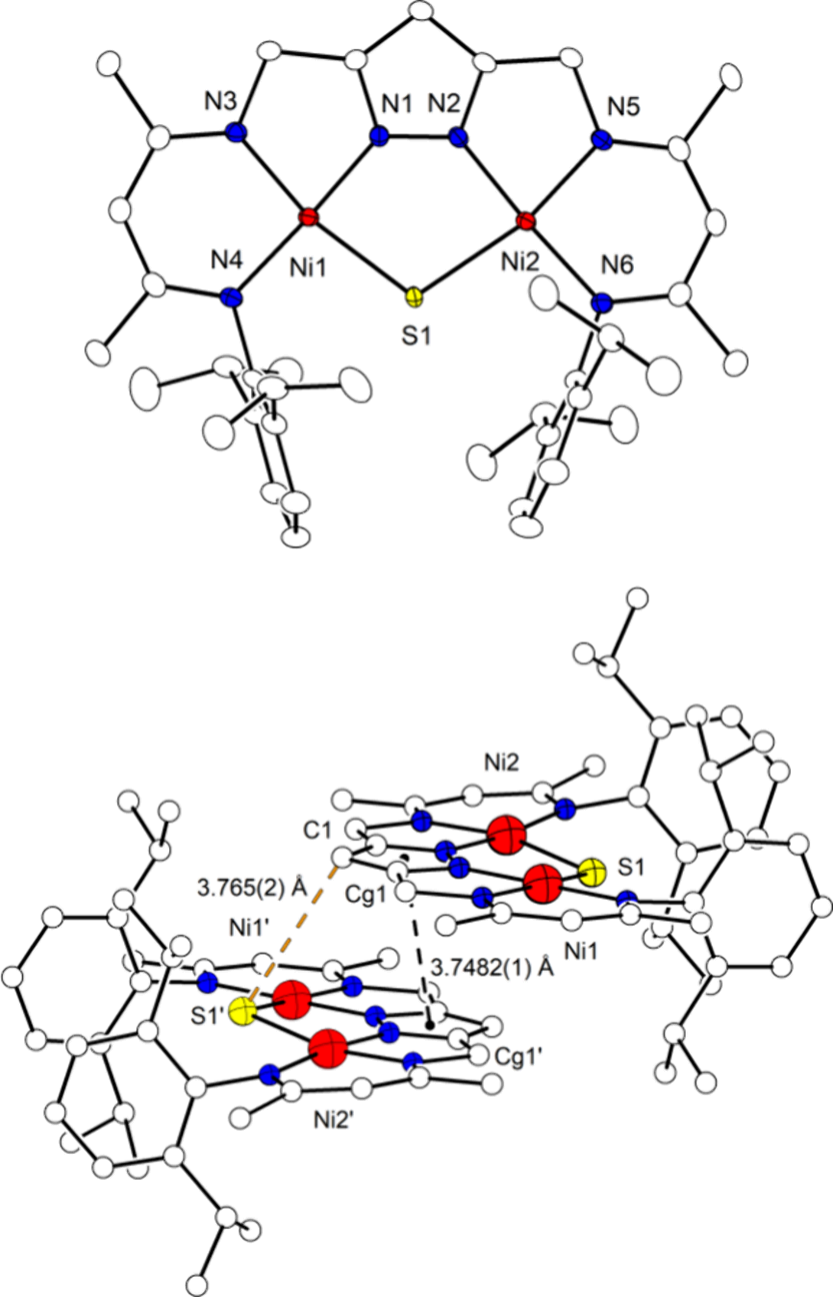
Top: plot (30% probability thermal ellipsoids) of the molecular structure of **6** (hydrogen atoms omitted for clarity). Bottom: plot emphasizing the head-to-tail packing of two [LNi_2_S] molecules in the crystal lattice. Cg1 is the centroid of the *pz*-atoms. Symmetry transformation used to generate equivalent atoms: (’) 1–*x*, 1–*y*, 1–*z*.

The molecular structure of neutral complex [LNi_2_S] (**6**) was determined by X-ray crystallography and is shown in Figure 10 (top). Its core is essentially identical to the one of μ-sulfido complexes **3**^**K**^ and **3**^**Na**^ (Table 1), but the alkali cations are now gone. The Ni···Ni distance is only slightly elongated by ∼0.05 Å (to 3.7047(5) Å) and the Ni–S bonds are slightly lengthened by ∼0.04 Å (to 2.2778(7)/ 2.2889(7) Å), which suggests a very small reorganization energy upon 1e^–^ redox interconversion of **3**^**K**^/**3**^**Na**^ and **6**. The CV of crystalline **6** dissolved in THF is essentially identical to the CVs of **3**^**K**^/**3**^**Na**^, displaying the reversible reduction of **6** at *E*_1/2_ = −1.17 V (Figures S26 and S39).

**Figure 11:**
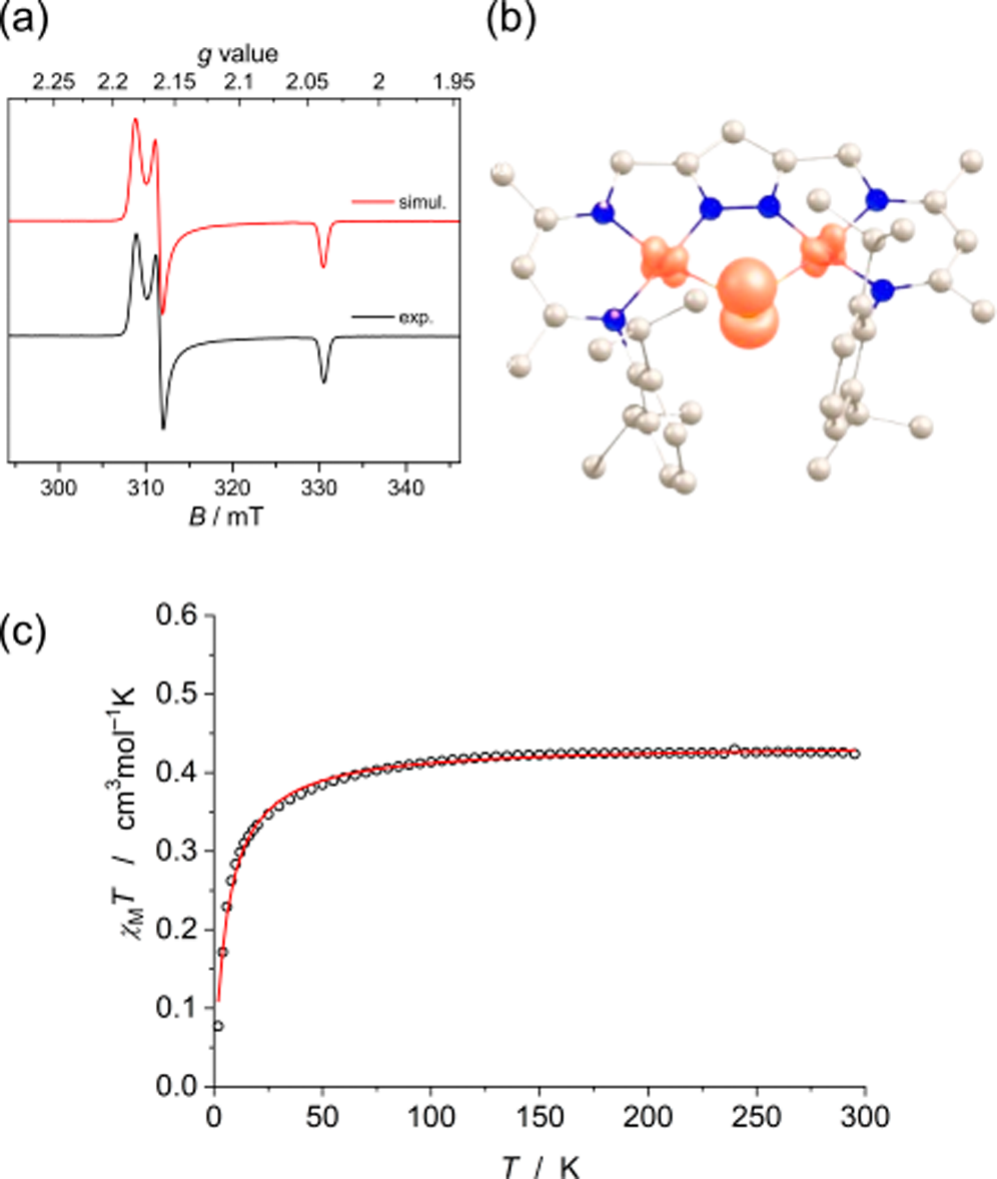
(a) X-band EPR spectrum of **6** in frozen MeTHF at 134 K (9.43 GHz, microwave power 5 mW). (b) Spin density plot of **6**. Mulliken spin population: Ni1 = 0.12, Ni2 = 0.13, S1 = 0.64. (c) Magnetic susceptibility data for **6** from 2–295 K. Data fitted for *S* = 1/2 spin system with *g*_av_ = 2.16 and Weiss temperature θ = −6.0 K.

SQUID magnetometry of solid crystalline **6** in the temperature range 2–295 K shows a χ_M_*T* value of 0.43 cm^3^ mol^–1^ K at high temperatures, which evidence that **6** is a paramagnetic *S* = 1/2 system with *g*_av_ = 2.16 (Figure 11c). The drop of χ_M_*T* below 50 K can be well modeled by assuming intermolecular antiferromagnetic interaction (Weiss temperature θ = −6.0 K corresponds to z*J*_inter_ = −16.7 cm^–1^; where *J*_inter_ is the interaction parameter between two nearest neighbor magnetic centers and z is the number of nearest neighbors), which may be explained by the close head-to-tail packing of two [LNi_2_S] molecules in the crystal lattice (Figure 10 bottom; suggesting z = 1). The distance of two neighboring *pz*-centroids is 3.7482(1) Å and the smallest distance of the S atom to one of the neighboring non-hydrogen atoms is to C1 with 3.765(2) Å (3.4961(6) Å to H1(-C1); 4.3849(6) Å to the closest neighboring centroid Cg1), which is somewhat higher than the sum of the van der Waals radii of S and C (3.5 Å).^49^

The X-band EPR spectrum of **6** in frozen 2-methyltetrahydrofuran (MeTHF) recorded at 134 K shows a rhombic spectrum with *g* values of 2.18, 2.16, and 2.04 (Figure 11a). A DFT optimized structure of [LNi_2_S] in the doublet ground state is in very good agreement with the structure of **6** obtained by X-ray diffraction. Mulliken population analysis (Figure 11b) indicates that the unpaired spin density is predominately located on the bridging S (64%) with minor contributions from the two nickel ions (12% and 13%). The SOMO is mostly composed of the S(p_*z*_) orbital that is perpendicular to the Ni–S–Ni plane. Thus, **6** can be best described as a dinickel(II) complex with a genuine bridging sulfur radical, S^•–^. The relatively large *g* anisotropic and rhombicity reflect a lifting of the degeneracy of the S(p_*x*_/p_*y*_) orbitals in the Ni–S–Ni plane.

**Figure 12:**
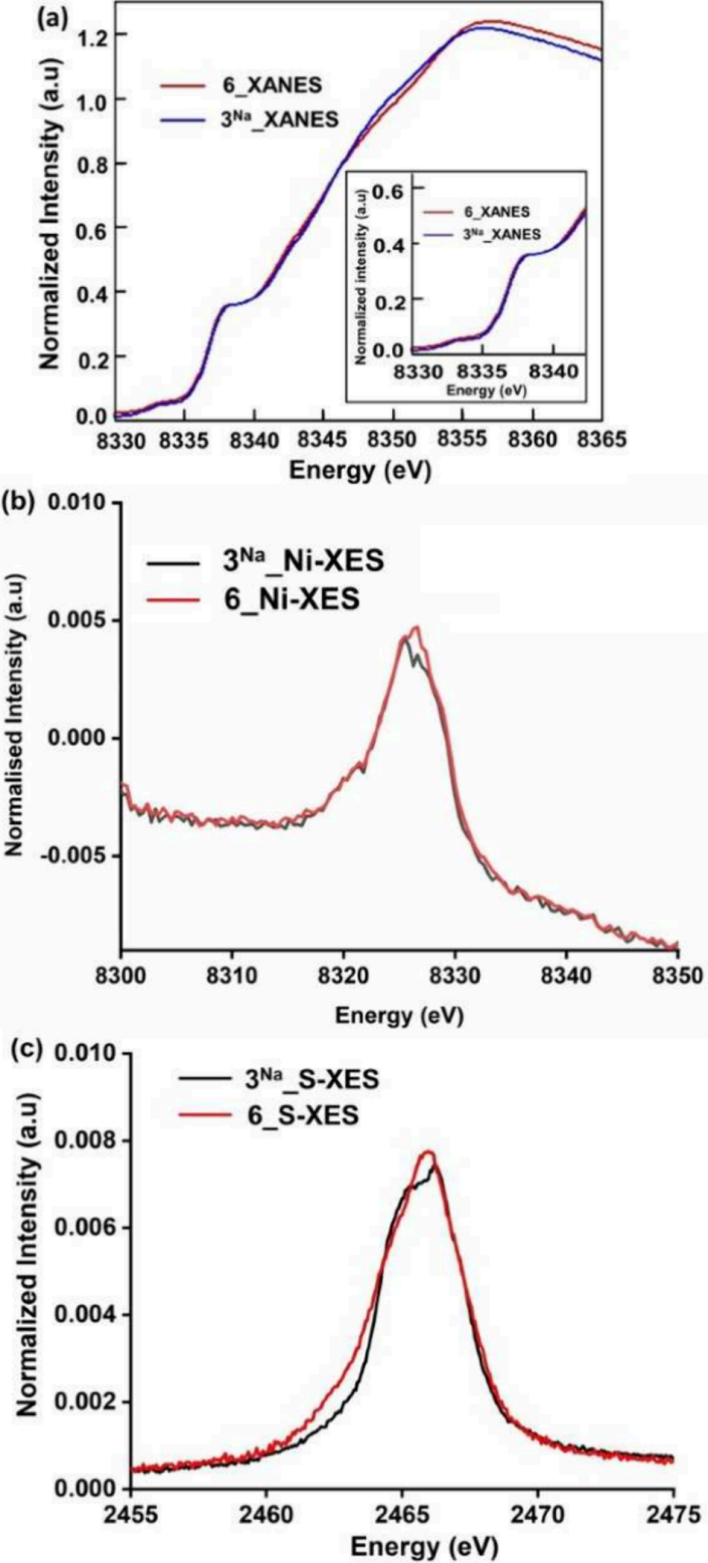
(a) Experimental Ni K-edge XAS spectra for complexes **3**^**Na**^ and **6**. (b) Experimental Ni VtC Kβ XES spectra for complexes **3**^**Na**^ and **6**. (c) Experimental S VtC Kβ XES spectra for complexes **3**^**Na**^ and **6**.

Further insight on the sulfido radical nature of the μ-S^•–^ ligand moiety in **6** was provided by X-ray absorption (XAS) and emission spectroscopies (XES). Figure 12a shows a comparison of the Ni K-edge XAS spectra of **6** and **3**^**Na**^. The edges are effectively superimposable and in both the cases consistent with a Ni^II^ assignment in which the 8333.4 eV pre-edge (Figure 12a inset) corresponds to a 1s to 3d transition and to higher energy at ∼8338 eV a 1s to 4p_*z*_ feature is observed, as expected for a planar Ni^II^ site.^50^ Further electronic structure characterization was obtained from Ni K-β XES and valence to core (VtC) data (Figure 11b). The Ni K-β mainline corresponds to 3p to 1s transitions, which will be modulated by 3p3d exchange in the final state. In both cases the spectra have no well-resolved *K*β’ feature and are consistent with low-spin Ni^II^ (Figure S55).^50^ Moreover, the Ni K-β mainline XES spectra for both the complexes **3**^**Na**^ and **6** (Figure S55) are identical, likely implicating no metal-based oxidation. The Ni VtC XES corresponds to transitions from filled ligand orbitals to the Ni 1s core hole (Figure 12b).^50,51^ These spectra show subtle modulations, which are reproduced by DFT calculations (See Section G of the SI and Figures S56–S58), but again show no evidence for a metal-based oxidation event. In contrast, the S XES (Figure 12c) show clear modulations with intensity growing on the low energy side in case of **6** (at ∼2462 eV) relative to **3**^**Na**^. Correlation of the experimental data to DFT calculations support that the observed spectra modulations derive from a sulfur-based oxidation in **6** (See Section G of the SI and Figures S59–S61).

### H-Atom Abstraction Thermochemistry and Reactivity of **6**

**Scheme 2:**
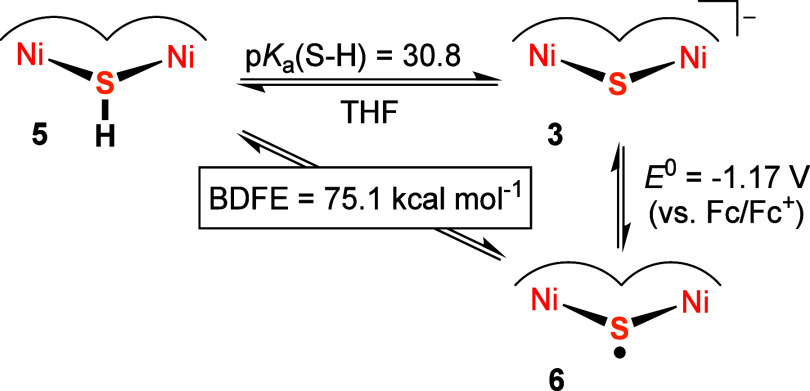
Parts of a Thermodynamic Scheme Involving μ-SH Complex **5**, μ-S^2–^ Complex **3** and μ-S^•–^ Complex **6**

With the experimentally determined redox potential *E*^0^ for the interconversion of **3** and **6** (assumed to equal *E*_1/2_) and the p*K*_a_ of **5** in hand, parts of a thermodynamic square scheme can be set up (Scheme 2) to derive a BDFE of 75.1 ± 1.0 kcal mol^–1^ for the S–H bond in the μ-SH complex **5** according to eq 1(25,52) where *C*_G,sol_ is equivalent to the H^+^/H^•^ standard reduction potential in the given solvent (with *C*_G,THF_ = 59.9 kcal mol^–1^).^25^
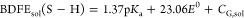
1

This value is lower than the S–H BDFEs of alkanethiols (around 84 kcal mol^–1^), thiophenol (77.7 kcal mol^–1^) or H_2_S (88.9 kcal mol^–1^) in water,^25^ but larger than S–H BDFEs of type **C** Cp*_2_Mo_2_S_4_(Me_n_H_m_) complexes which were found in the range 43–68 kcal mol^–1^ in MeCN^29^ or BDFE(S–H) = 69.4 ± 1.7 kcal mol^–1^ reported for complex (OC)_3_Fe(μ-SCH_3_)(μ-SH)Fe(CO)_3_ (**B**) in MeCN.^28^ Gas phase DFT calculations provided an estimate BDFE(S–H) = 70 kcal mol^–1^ of the terminal iron sulfide complex **F**. ^23^

While sulfur radical complex **6** is a poor oxidant, this is compensated by a very high basicity of the μ-S^2–^ complex **3**, resulting in a comparatively high BDFE(S–H) for a metal-bound SH group. Addition of H[DBU]PF_6_ (DBU is diazabicyclononane; p*K*_a_ = 19.1 in THF)^43^ to a solution of **6** in THF did not lead to any observable changes in the UV–vis spectrum (Figure S45). This indicates that the difference in p*K*_a_ for the μ-S^2–^ complex **3** and the μ-S^•–^ complex **6** is at least 12 units, and that thermodynamic coupling of the e^–^ and H^+^ transfers interconverting **5** and **6** is strong.

**Figure 13:**
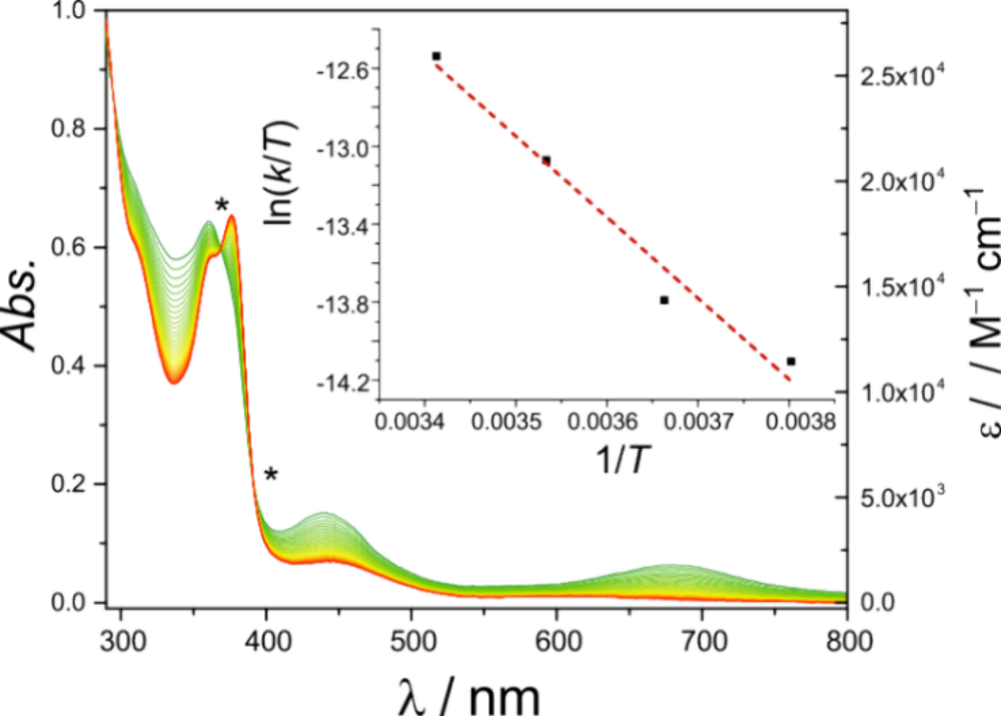
UV–vis monitoring of the reaction of **6** (10^–4^ M solution in THF) with 300 equiv of TEMPO-H at 283 K; spectra recorded every 120 s. Isosbestic points are indicated by an asterisk. The inset shows the plot of ln(*k*/*T*) versus inverse temperature (Eyring plot) for measurements at 263, 273, 283, and 293 K.

Complex **6** reacts quantitatively with TEMPO-H (BDFE(O–H) = 65.5 kcal mol^–1^ in THF^53^; calculated reaction free energy Δ*G*° = −9.6 kcal mol^–1^; see SI) under pseudo first-order conditions to give μ-SH complex **5**; the reaction can be conveniently monitored by UV–vis spectroscopy, showing the disappearance of the characteristic bands at 442 and 680 nm for **6** and the blue shift of the band at 373 to 365 nm (Figure 13). The final UV–vis spectrum matches the one of pristine **5** (cf. Figure S37). Eyring analysis gave activation parameters Δ*H*^‡^ = (8.2 ± 1.0) kcal mol^–1^ and Δ*S*^‡^ = −(44 ± 4) cal mol^–1^ K^–1^, which translates into an activation free energy Δ*G*^‡^_293_ = (21.1 ± 1.9) kcal mol^–1^. The free energies for initial electron transfer to give **3**, Δ*G*°_ET_, as well as for initial proton transfer to give putative **6H**^**+**^, Δ*G*°_PT_ and, are much larger (>39 kcal mol^–1^; see SI for calculations). Hence the stepwise ET/PT and PT/ET processes are largely disfavored and it can be safely concluded that the reaction proceeds via concerted proton–electron transfer (CPET). This is in line with the strong thermodynamic coupling of the e^–^ and H^+^ transfers for interconverting **6** and **5**, as mentioned above. The large negative activation entropy Δ*S*^‡^ suggests an associative CPET scenario via precursor complex formation.

μ-S^•–^ complex **6** also reacts cleanly with the C–H substrate xanthene (BDFE(C–H) = 72.2 kcal mol^–1^ in THF),^54^ albeit slower than with TEMPO-H in accordance with the lower driving force Δ*G*° (second order rate constant *k* = 2.30 × 10^–4^ M^–1^·s^–1^ at 293 K, Figures S43 and S44; compared to *k* = 1.16 × 10^–3^ M^–1^·s^–1^ for TEMPO-H). In contrast, no reaction was observed with dihydroanthracene (BDFE(C–H) = 76.3 kcal mol^–1^ in THF),^54^ which defines a BDFE window for the S–H bond in **5** that is well in agreement with BDFE(S–H) = 75.1 ± 1.0 kcal mol^–1^ derived from the potential/p*K*_a_ method. For 2,4,6-tri(*tert*-butyl)phenol BDFE(O–H) = 74.4 kcal/mol in THF has been reported,^53^ which is the same as BDFE(S–H) of **5** within experimental error. Complex **5** was found to slowly react with two equivalents of 2,4,6-tri(*tert*-butyl)phenoxy radical (TTBP) to generate **6** in a backward HAT (Scheme 1; see SI for experimental details).

## Summary and Conclusions

The highly preorganized dinickel(II) scaffold {LNi_2_} has allowed for the stabilization and transformation of a variety of S-based moieties within its bimetallic pocket. To that end the dihydrides [MLNi_**2**_H_2_] (**1**^**M**^; M = K, Na) serve as a convenient entry point for the synthesis of disulfido- and sulfido-bridged complexes [MLNi_2_(S_2_)] (**2**^**M**^) and [MLNi_2_S] (**3**^**M**^) via H_2_-releasing reductive activation of S_8_ or SPMe_3_, respectively. Oxidation of **3**^**M**^ was found to occur at very low redox potential (*E*_1/2_ = −1.17 V), and the resulting complex [LNi_2_S] (**6**) with *S* = 1/2 ground state can be best described as a dinickel(II) complex with a genuine bridging sulfur radical, S^•–^, as confirmed by a combination of SQUID magnetometry, EPR and XAS/XES spectroscopies and DFT calculations. In **6**, the SOMO is mostly composed of the S(p_*z*_) orbital that is perpendicular to the Ni–S–Ni plane, involved in π interactions with the two Ni^II^ ions. The anionic character of the sulfido complexes [LNi_2_S]^−^ (anionic parts of **3**^**M**^), as well as the preference of the bis(tridentate) ligand L^3–^ to enforce a square-planar environment of the Ni^II^ ions (d^8^, *S* = 0) in type [LNi_2_(μ-X)] complexes while restricting redox processes to the bridging ligand X in the bimetallic pocket, likely contribute to the facile formation of this unique sulfur radical complex. Furthermore, the bulky aryl substituents shielding the bimetallic pocket in **6** prevent the formation of intermolecular S–S bonds. With this platform, the entire series of complexes [MLNi_2_S] (**3**^**M**^), [LNi_2_S] (**6**) and [LNi_2_(SH)] (**5**) could be structurally characterized, representing the relevant trio of the thermodynamic square scheme for PCET chemistry at M–(μ-SH/μ-S)–M sites. The structures and metric parameters of the Ni–(μ-SH/μ-S)–Ni cores of **3**^**M**^, **6** and **5** are very similar, indicating that e^–^ and H^+^ transfers require very little reorganization. From the experimentally determined *E*_1/2_ and p*K*_a_ values a BDFE of 75.1 ± 1.0 kcal mol^–1^ for the S–H bond in the μ-SH complex **5** could be derived, which is larger than the very few BDFEs for metal-bridging μ-SH groups reported so far (in the range 43–70 kcal mol^–1^ in MeCN)^28,29^ or the recently estimated BDFE of a terminal {Fe^II^–SH} complex,^23^ albeit smaller than the typical S–H BDFEs of H_2_S and thiols.^25^ The S–H BDFE value for **5** derived from the potential/p*K*_a_ method has been corroborated by its reactivity: the μ-S^•–^ complex **6** readily reacts with TEMPO-H and substrates with weak C–H bonds such as xanthene to give **5**, while **6** is reformed upon reaction of μ-SH complex **5** with a phenoxy radical that forms an equally strong O–H bond. This study thus demonstrates the ability of M–(μ-SH/μ-S)–M to engage in S-centered PCET reactivity even with C–H substrates. A structural foundation is provided and a thermodynamic framework is established, which is of particular interest in view of the prominence of M-(μ-SH/μ-S)-M units in biological systems and the proposed relevance of S-mediated X–H/C–H bond activation steps in metal sulfide based heterogeneous catalysis. It will be interesting to evaluate how the identity of the metal ions, the degree of redox confinement to the μ-S atom or structural variations of the M–(μ-SH/μ-S)–M core effect the thermochemistry and reactivity.

## Experimental Section

### Materials and Methods

All experiments and manipulations were carried out under dry oxygen-free Argon using standard Schlenk techniques, or in a glovebox filled with dinitrogen (O_2_ < 0.5 ppm, H_2_O < 0.5 ppm). Solvents were dried by standard methods and freshly distilled prior use. THF, pentane and hexanes were dried over sodium in the presence of benzophenone; THF–d_8_ was also dried over sodium in the presence of benzophenone and stored over 3 Å molecular sieve. K was purchased as a dispersion in mineral oil and was washed repetitively with hexanes and then dried in vacuum prior to use. The starting materials [ML(Ni–H)_2_] (**1^M^**; M = Na, K) were prepared according to the literature procedure.^31^ TEMPO-H was prepared as described in literature. UV–vis spectra were recorded on an Agilent Cary 60 equipped with an Unisoku Cryostat (CoolSpek) and magnetic stirrer using quartz cuvettes with an attached tube and a screw cap with a septum. All UV–vis samples were prepared in a glovebox and transferred out of the glovebox prior to the measurement. Infrared spectra were recorded inside a glovebox on a Cary 630 FTIR spectrometer equipped with Dial Path Technology and analyzed by FTIR MicroLab software. ESI-MS spectra were recorded on Bruker HCT ultra spectrometer. Elemental analyses were performed by the analytical laboratory of the Institute of Inorganic Chemistry at the University of Göttingen using an Elementar Vario EL III instrument. ^1^H and ^13^C NMR spectra were recorded on Bruker Avance 300 or 400 or 500 spectrometers. Chemical shifts are reported in parts per million relative to residual proton and carbon signals of the solvent THF (δ_H_ = 1.73 and 3.59 ppm; δ_C_ = 25.31 and 67.21 ppm).

### Magnetic Measurements

Temperature-dependent magnetic susceptibility measurements for **6** were carried out with a Quantum-Design MPMS3 SQUID magnetometer equipped with a 7 T magnet in the range from 295 to 2.0 K at a magnetic field of 0.5 T. The powdered samples were contained in a polycarbonate capsule and fixed in a nonmagnetic sample holder. Each raw data file for the measured magnetic moment was corrected for the diamagnetic contribution of the capsule according to *M*^dia^ = χ_*g*_·*m*·*H*, with experimentally obtained gram susceptibilities of the capsule. The molar susceptibility data of the compounds were corrected for the diamagnetic contribution. Experimental data for **6** were modeled with the julX program^55^ using a fitting procedure to the spin Hamiltonian:
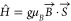
2

### EPR Measurement

EPR spectra were measured with a Bruker E500 ELEXSYS X-band spectrometer equipped with a standard cavity (ER4102ST, 9.43 GHz). The sample temperature was maintained constant with an Oxford instrument Helium flow cryostat (ESP910) and an Oxford temperature controller (ITC-4). The microwave frequency was measured with the built-in frequency counter and the magnetic field was calibrated by using an NMR field probe (Bruker ER035M). EPR spectra were simulated using Easy-Spin.^56^

### Electrochemistry

Cyclic voltammetry (CV) experiments were performed with an Interface 1000B potentiostat using a three electrode setup consisting of a glassy carbon working electrode, a platinum wire counter electrode and an Ag reference electrode, and were analyzed by Gamry Framework software. CV experiments were performed in deoxygenated THF containing NBu_4_PF_6_ (0.1 M) as supporting electrolyte; decamethylferrocene (Fc*) was used as an internal standard and potentials are referenced vs ferrocene, using *E*_1/2_(Fc*^+^/Fc*) = −0.45 V vs Fc^+^/Fc in THF (see Figure S26 and ref (48)). Spectroelectrochemistry experiments were carried out with the same instrument and the same standard setup in a CHI cell in a HP8453 UV–vis spectrophotometer.

### XAS and XES Measurements

Ni K-edge X-ray absorption data were measured at the SuperXAS beamline of the Swiss Light Source (SLS, Switzerland), and Ni and S valence-to-core X-ray Emission Spectroscopy (VtC XES) data collection was done at the PINK tender X-ray beamline at BESSY II. For XAS experiments, solid samples were diluted with boron nitride to achieve a 2% (w/w) concentration of nickel, then packed into 1 mm thick aluminum sample cells and sealed with 13 μm Kapton tape. For XES experiments, all samples were measured in the solid state. For Ni XES, the pure solids were ground to a fine powder and packed into 0.5 mm thick aluminum sample holders and sealed with 13 μm Kapton tape. For S XES, the pure solids were ground to a fine powder and packed into 0.5 mm thick aluminum sample holders and sealed with polypropylene. Details on the data acquisition and handling are provided in the Supporting Information, section G.

### DFT Calculations

ORCA, versions 4.2.1 and 5.0.3 has been used for all calculations.^57^ Details are provided in the Supporting Information, section G.

### Single-Crystal X-ray Structure Determinations

Crystal data and details of the data collections are provided in the Supporting Information, section F (Tables S2 and S3); selected bond lengths and angles are listed in Table 1, molecular structures are shown in Figures S49–S54. X-ray data were collected on a STOE IPDS II or a BRUKER D8-QUEST diffractometer (monochromated Mo–Kα radiation, λ = 0.71073 Å) by use of ω or ω and ϕ scans at low temperature. The structures were solved with SHELXT and refined on *F*^2^ using all reflections with SHELXL.^58^ Face-indexed absorption corrections were performed numerically with the program X-RED^59^ or by the multiscan method with SADABS.^60^

#### [KLNi_2_(S_2_)] (**2^K^**)

Elemental sulfur (2.56 mg, 0.0400 mmol, 1.00 equiv) was added to a solution of **1**^**K**^ (30.5 mg, 0.0400 mmol, 1.00 equiv) in THF (1 mL) at −35 °C. The color change from orange to blood red was accompanied by immediate gas evolution. The solution was stirred for 2 h at −35 °C. The solvent was then removed under reduced pressure. Suitable crystals for X-ray diffraction were obtained by layering hexanes on a solution of **2**^**K**^ in THF at −35 °C. (Yield: > 95%, from ^1^H NMR monitoring). ^**1**^**H NMR** (THF-*d*_8_, 500 MHz) = 6.87 (m, 6H, Ar), 5.62 (s, 1H, 4-Pz), 4.64 (s, 2H, C**H**CCH_3_), 4.09 (s, 4H, C**H**_2_Pz), 3.60–3.50 (m, 4H, C**H**(CH_3_)_2_), 1.90 (s, 6H, C**H**_3_), 1.40 (d, 12H, *J*_H–H_ = 7 Hz, CH(C**H**_3_)_2_), 1.20 (d, 6H, ^3^*J*_H–H_ = 7 Hz, CH(C**H**_3_)_2_), 1.02 (s, 12H, C**H**_3_).^**13**^**C NMR** (THF-*d*_8_, 100 MHz) = 159.9 (*C*^q^-Me), 158.0 (*C*^q^-Me), 156.4 (3(5)C-^Pz^), 152.7 (Ar), 143.8 (Ar), 123.4 (Ar), 122.7 (Ar), 97.4 (**C**HCCH_3_), 92.0 (4C-Pz), 51.9 (CH_2_Pz) 28.6 ((**C**H_3_)_2_CH), 26.6 (**C**H_3_^iPr^), 24.5 (**C**H_3_^iPr^), 22.3 (**C**H_3_). **ATR-IR** (ν/cm^–1^) = 3052 (w), 2954 (m), 2924 (m), 2862 (m), 1555 (m), 1528 (vs), 1462 (m), 1433 (vs), 1398 (vs), 1315 (m), 1276 (m), 1249 (m), 1232 (w), 1209 (w), 1188 (w), 1124 (w), 1097 (w), 1054 (m), 1030 (m), 954 (w), 934 (w), 900 (w), 856 (w), 795 (m), 744 (s), 729 (m), 713 (m), 646 (w), 625 (w), 547 (w), 521 (w), 425 (m). **UV–vis** (THF): λ_max_ (ε/M^–1^ cm^–1^) = 270 (20200), 371 (13500), 472 (5000), 529 (4200) nm. **Anal. Calcd** (%) for [KNi_2_(C_39_H_53_N_6_)S_2_·(THF)_2_]: C 58.15, H 7.16, N 8.66, S 6.61; found: C 58.98, H 7.16, N 9.52, S 6.53.

#### [KLNi_2_(μ-S)] (**3^K^**)

*Method A.* Excess PPh_3_ (23.6 mg, 0.09 mmol, 3.00 equiv) was added to a solution of **2**^**K**^ (24.0 mg, 0.03 mmol, 1.00 equiv) in THF-*d*_8_ at rt. Full conversion happened in around 40 h. Red block crystals suitable for X-ray diffraction were obtained within 2 days by layering hexanes on a solution of **3**^**K**^ in THF at −30 °C. *Method B.* S=PMe_3_ (5.20 mg, 0.048 mmol, 1.20 equiv) was added to a solution of **1**^**K**^ (30.5 mg, 0.040 mmol, 1.00 equiv) in THF (1 mL) at rt. The mixture darkened upon addition of S=PMe_3_. After stirring for 3 days at 40 °C, the solution was dried under reduced pressure. Red block crystals suitable for X-ray diffraction were obtained within 2 weeks by layering hexanes on a solution of crude **3**^**K**^ in THF at −30 °C. (Yield: > 95%, from ^1^H NMR monitoring). ^**1**^**H NMR** (THF-*d*_8_, 400 MHz) = 6.79 (m, 2H, ^3^J_H–H_ = 4 Hz, Ar), 6.67 (d, 4H, ^2^J_H–H_ = 8 Hz, Ar) 5.46 (s, 1H, 4-Pz), 4.47 (s, 2H, CHCCH_3_), 4.12 (s, 4H, CH2Pz), 3.42 (m, 4H, CH(CH_3_)_2_), 1.82 (s, 6H, CH_3_), 1.33 (d, 12H, ^2^J_H–H_ = 8 Hz, (CH_3_)_2_CH), 1.16 (s, 6H, CH_3_), 0.95 (d, 12H, ^2^J_H–H_ = 8 Hz, (CH_3_)_2_CH). ^**13**^**C NMR** (THF-*d*_8_, 100 MHz) = 159.2, 158.8, 153.9 (3(5)C-^Pz^), 149.8, 141.5, 124.3, 123.3, 96.1 (**C**HCCH_3_), 91.3 (4-Pz), 53.9 ((3),(5)C-Pz), 28.7 (CH_3_), 26.4 (CH_3_), 25.4 (CH_3_), 21.6 (CH_3_). **ATR-IR** (ν/cm^–1^) = 3056 (w), 2954 (s), 2928 (s), 2861 (s), 1549 (s), 1520 (vs), 1458 (s), 1431 (s), 1400 (vs), 1377 (w), 1366 (w), 1321 (w), 1308 (vs), 1288 (w), 1252 (m), 1231 (m), 1190 (m), 1079 (m), 1055 (vs), 1027 (m), 1005 (w), 950 (w), 938 (w), 894 (m), 855 (w), 806 (w), 793 (s), 754 (vs), 726 (vs), 711 (vs), 659 (w), 640 (w), 542 (w), 523 (m). **ESI-MS** (THF:CH_3_CN = 10:1): *m*/*z* (%) = 753.32 (**2**–K)^−^. **UV–vis** (THF): λ_max_ (ε/M^–1^ cm^–1^)= 373 (15400), 458 (5400) nm. **Anal. Calcd** (%) for [KNi_2_(C_39_H_53_N_6_)S·(THF)_2_]: C 60.14, H 7.41, N 8.95, S 3.42; found: C 59.63, H 7.40, N 8.58, S 2.93.

#### [NaLNi_2_(μ-S)] (**3^Na^**)

S=PMe_3_ (5.20 mg, 0.0480 mmol, 1.20 equiv) was added to a solution of **1**^**Na**^ (30.5 mg, 0.0400 mmol, 1.00 equiv) in THF (1 mL) at rt. No obvious color change was observed. After stirring for 3 days at 40 °C, the solution was dried under reduced pressure. Red block crystals suitable for X-ray diffraction were obtained within 2 weeks by layering hexanes on a solution of crude **3**^**Na**^ in THF at −30 °C. (Yield: > 95%, from ^1^H NMR monitoring). ^**1**^**H NMR** (THF-*d*_8_, 400 MHz) = 6.80 (m, 2H, ^3^J_H–H_ = 4 Hz, Ar), 6.67 (d, 4H, ^2^J_H–H_ = 8 Hz, Ar) 5.50 (s, 1H, 4-Pz), 4.50 (s, 2H, CHCCH_3_), 4.15 (s, 4H, CH_2_Pz), 3.48 (m, 4H, CH(CH_3_)_2_), 1.85(s, 6H, CH_3_), 1.28 (d, 12H, ^2^J_H–H_ = 8 Hz, (CH_3_)_2_CH), 1.16 (s, 6H, CH_3_), 0.95 (d, 12H, ^2^J_H–H_ = 8 Hz, (CH_3_)_2_CH). **UV–vis** (THF): λ_max_ (ε/M^–1^ cm^–1^)= 373 (15500), 458 (5400) nm. **Anal. Calcd** (%) for [NaNi_2_(C_39_H_53_N_6_)S·(THF)_2_]: C 61.19, H 7.54, N 9.11, S 3.48; found: C 60.42, H 7.43, N 9.12, S 3.53.

#### LNi_2_(μ-SCH_3_) (**4**)

A solution of **3**^**K**^ (16.0 mg, 0.02 mmol, 1.00 equiv) in THF (2 mL) was treated with excess CH_3_OTs. The solution color changed from orange to green immediately, and the reaction mixture was stirred at rt for 30 min. Green block-shaped crystals suitable for X-ray diffraction were obtained by layering hexanes on a solution of crude **4** in THF at −30 °C; yield 70%. ^**1**^**H NMR** (THF-*d*_8_, 400 MHz) = 7.01 (m, 2H, ^3^J_H–H_ = 4 Hz, Ar), 6.89 (d, 4H, ^2^J_H–H_ = 8 Hz, Ar) 5.61 (s, 1H, 4-Pz), 4.76 (s, 2H, CHCCH_3_), 4.26 (s, 4H, CH2Pz), 3.34 (m, 4H, CH(CH_3_)_2_), 2.33 (s, 3H, SCH_3_),, 2.02 (s, 6H, CH_3_),, 1.28 (s, 6H, CH_3_), 1.19 (d, 12H, ^2^J_H–H_ = 8 Hz, (CH_3_)_2_CH), 1.02 (d, 12H, ^2^J_H–H_ = 8 Hz, (CH_3_)_2_CH). ^**13**^**C NMR** (THF-*d*_8_, 100 MHz) = 159.8 (*C*^q^-Me), 153.1 (C-^Pz^), 148.3 (C-^Pz^), 141.9 (Ar), 133.0 (Ar), 125.0, 123.0 (Ar), 97.0 (**C**HCCH_3_), 91.1 (4-Pz), 65.3 (CH_2_Pz), 54.1 (CH_2_Pz), 28.7 (CH_3_), 27.8 (CH_3_), 20.5 (CH_3_), 17.7 (CH_3_), 14.8 (CH_3_). **ATR-IR** (ν/cm^–1^) = 3052 (w), 2953 (m), 2922 (m), 2864 (m), 1550 (m), 1528 (vs), 1460 (w), 1435 (s), 1394 (vs), 1380 (vs), 1312 (vs), 1266 (w), 1251 (w), 1178 (m), 1084 (m), 1055 (m), 1033 (m), 991(m), 940 (w), 799 (s), 763 (vs), 739 (vs), 595 (w), 546 (w), 527 (w), 463 (w), 437 (m), 404 (m). **Anal. Calcd** (%) for [Ni_2_(C_39_H_53_N_6_)SCH_3_]: C 62.36, H 7.33, N 10.91, S 4.16; found: C 62.63, H 7.42, N 10.19, S 4.75.

#### [LNi_2_(SH)] (**5**)

Treatment of **3**^**K**^ (33.0 mg, 0.04 mmol, 1.00 equiv) in THF (2 mL) with [HLut]OTf (10.3 mg, 0.04 mmol, 1.00 equiv) resulted in an immediate color change from red to brown. The reaction mixture was stirred for 1 h. After filtration, crystals suitable for X-ray diffraction were obtained by layering hexanes on a solution of **5** in THF at −30 °C; yield: 80%. ^**1**^**H NMR** (THF-*d*_8_, 400 MHz) = 6.96 (d, 2H, *J*_H–H_ = 12 Hz, Ar), 6.85 (d, 4H, *J*_H–H_ = 8 Hz, Ar), 5.55 (s, 1H, 4-Pz), 4.75 (s, 2H, C**H**CCH_3_), 4.23 (s, 4H, CH_2_Pz), 3.29 (m, 4H, C**H**(CH_3_)_2_), 1.99 (s, 6H, C**H**_3_CCH), 1.49 (d, 12H, *J*_H–H_ = 4 Hz, CH(C**H**_3_)_2_), 1.29 (s, 6H, C**H**_3_CCH), 1.01 (d, 12H, *J*_H–H_ = 8 Hz, CH(C**H**_3_)_2_), −3.61 (s, 1H, SH). ^**13**^**C NMR** (THF-*d*_8_, 100 MHz) = 162.0 (*C*^q^-Me), 159.9 (*C*^q^-Me), 154.1 (C-^Pz^), 149.6 (C-^Pz^), 141.8 (Ar), 129.8 (Ar), 124.8 (Ar), 97.9 (**C**HCCH_3_), 92.3 (4-Pz), 55.6 (CH_2_Pz), 32.7 (**C**H_3_), 30.8 (**C**H_3_), 29.0 (**C**H_3_), 23.7 (**C**H_3_), 21.7 (**C**H_3_) 14.5 (**C**H_3_). **ATR-IR** (ν/cm^–1^) = 3056 (m), 2951 (m), 2924 (m), 2864 (w), 2500 (s) (SH), 1556 (m), 1530 (vs), 1464 (vs), 1434 (vs), 1398 (vs), 1359 (m), 1313 (s), 1282 (w), 1250 (m), 1233 (w), 1191 (w), 1126 (w), 1108 (w), 1087 (w), 1075 (w), 1056 (w), 1032 (m), 1009 (w), 983 (w), 934 (w), 916 (w), 860 (w), 795 (s), 760 (vs), 741 (vs), 714 (w), 641 (w), 543 (w), 529 (w). **UV–vis** (THF): λ_max_ (ε/M^–1^ cm^–1^) = 270 (25700), 309 (15200), 375 (16300), 450 (1800), 620 (200). **Anal. Calcd** (%) for [Ni_2_(C_39_H_53_N_6_)SH]: C 61.93, H 7.20, N 11.11, S 4.24; found: C 60.48, H 7.10, N 10.17, S 4.40.

#### [LNi_2_(μ-S)] (**6**)

*Method A.* To a precooled and blood red solution of **3**^**K**^ (16.0 mg, 0.020 mmol, 1.00 equiv) in THF (2 mL) was added [Cp*_2_Fe][BF_4_] (6.55 mg, 0.024 mmol, 1.20 equiv). The reaction mixture was allowed to react or 2 h at −35 °C. The solvent was removed under vacuum and the residue washed with cold THF (3 × 1 mL), then dissolved in a minimum amount of toluene and filtered. Emerald crystals suitable for X-ray diffraction were obtained by layering hexanes on a solution of the toluene filtrate at −30 °C; yield: 50%. *Method B*. A 100 mL Schlenk flask was charged with complex **3**^**K**^ (30.5 mg, 0.04 mmol, 1.0 equiv) and THF (2 mL). The solution was degassed by the freeze–pump–thaw method, then exposed to 1.0 equiv of dried dioxygen for 2 h at −40 °C while stirring. The color of the solution changed from orange to green. The mixture was then evaporated and the residue dissolved in THF and filtered. Green block-shaped crystals suitable for X-ray diffraction were obtained by layering hexanes on a solution of crude **6** in THF at −30 °C; yield 70%. **ATR-IR** (ν̃/cm^–1^) = 3058 (w), 2956 (m), 2924 (m), 2865 (m), 1553 (m), 1532 (s), 1461 (s), 1437 (s), 1394 (s), 1369 (s), 1313 (s), 1252 (s), 1234 (s), 1187 (s), 1176 (s), 1092 (m), 1032 (s), 982 (s), 936 (m), 916 (m), 870 (w), 797 (s), 759 (s), 743 (s), 714 (m), 588 (m), 565(m). **UV–vis** (THF): λ_max_ (ε/M^–1^ cm^–1^) = 270 (2500), 361 (16100), 442 (3300), 680 (1300) nm. **Anal. Calcd** (%) for [Ni_2_(C_39_H_53_N_6_)S·(THF)_0.5_]: C 62.23, H 7.26, N 10.62, S 4.05; found: C 63.32, H 7.48, N 10.23, S 3.97.

## Supporting Information Available

NMR and IR spectra; ESI mass spectra; additional UV–vis spectra, kinetic data and electrochemical measurements; detailed crystallographic information; details about XAS/XES measurements and DFT calculations (PDF)
