# Supplementary material for: Cooperative Sulfur Transformations at a Dinickel Site: A Metal Bridging Sulfur Radical and Its H-Atom Abstraction Thermochemistry
Source: J Am Chem Soc. 2024 Aug 7;146(33):23158–70. doi: 10.1021/jacs.4c05113 (PMC11345757; doi:10.1021/jacs.4c05113)
Supplement: Supplementary file 1 — ja4c05113_si_001.pdf [file ja4c05113_si_001.pdf]

Supporting Information for

Cooperative Sulfur Transformations at a Dinickel Site: A Metal Bridging Sulfur Radical and its H-Atom Abstraction Thermochemistry

Valeria Tagliavini,[a,§] Peng-Cheng Duan,[a,§,#] Sayanti Chatterjee,[b,c] Eleonora Ferretti,[a] Sebastian Dechert,[a] Serhiy Demeshko,[a] Liqun Kang,[b] Sergey Peredkov,[b] Serena DeBeer[b] and Franc Meyer[a,d]*

[a] University of Göttingen, Institute of Inorganic Chemistry, Tammannstr. 4, D-37077 Göttingen, Germany

[b] Max Planck Institute for Chemical Energy Conversion, Stiftstrasse 34-36, D-45470 Mülheim an der Ruhr, Germany

[c] Indian Institute of Technology Roorkee, Department of Chemistry, Roorkee 247667, Uttarakhand, India

[d] University of Göttingen, International Center for Advanced Studies of Energy Conversion (ICASEC), Tammannstr. 6, D-37077 Göttingen, Germany

[§] V.T. and P.-C.D. contributed equally to the work

Present address:
[#] Henan University, Key Laboratory for Special Functional Materials of Ministry of Education, National and Local Joint Engineering Research Center for High-Efficiency Display and Lighting Technology, School of Materials Science and Engineering, 475004, Kaifeng, China

* To whom correspondence should be addressed. Email: franc.meyer@chemie.uni-goettingen.de

Table of Contents

A Complex 2^K S3
A.1 NMR spectra S3
A.2 UV-vis spectra S6
A.3 IR spectrum S7
A.4 CV measurements S8
B Complexes 3^K and 3^{Na} S9
B.1 NMR spectra S10
Monitoring of the reaction of $\mathbf{2^K}$ with PPh_3 ..... S10
Direct synthesis of $\mathbf{3^M}$ from $\mathbf{1^M}$ S12
B.2 IR spectrum S16
B.3 ESI mass spectrum S17
B.4 UV-vis spectra S18
B.5 Titration experiments S19
B.6 pK_a determination S21
B.7 CV measurements S23
B.8 UV-vis SEC of $\mathbf{3^{Na}}$ S25
C Complex 4 ..... S26
C.1 NMR spectra S26
C.2 IR spectrum S28
D Complex 5 ..... S29
D.1 NMR spectra S29
D.2 IR spectra S32
D.3 UV-vis spectrum S33
D.4 CV measurement S34
E Complex 6 ..... S35
E.1 CV measurements S35
E.2 Substrate reactivity S36
E.3 Protonation of radical complex **6** S40
E.4 Comparison of concerted and stepwise pathways S41
E.5 Mass spectrometry S43
E.6 Reaction of **5** with phenoxy radical TTBP to give **6** S43
E.7 IR spectrum S44
F X-ray Crystallography S45
G XES and XAS Data and DFT Calculations S54
H References S65

A Complex 2^{K}

A.1 NMR spectra

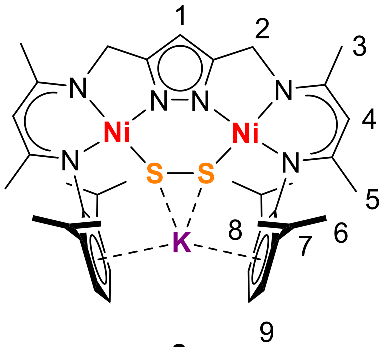

2

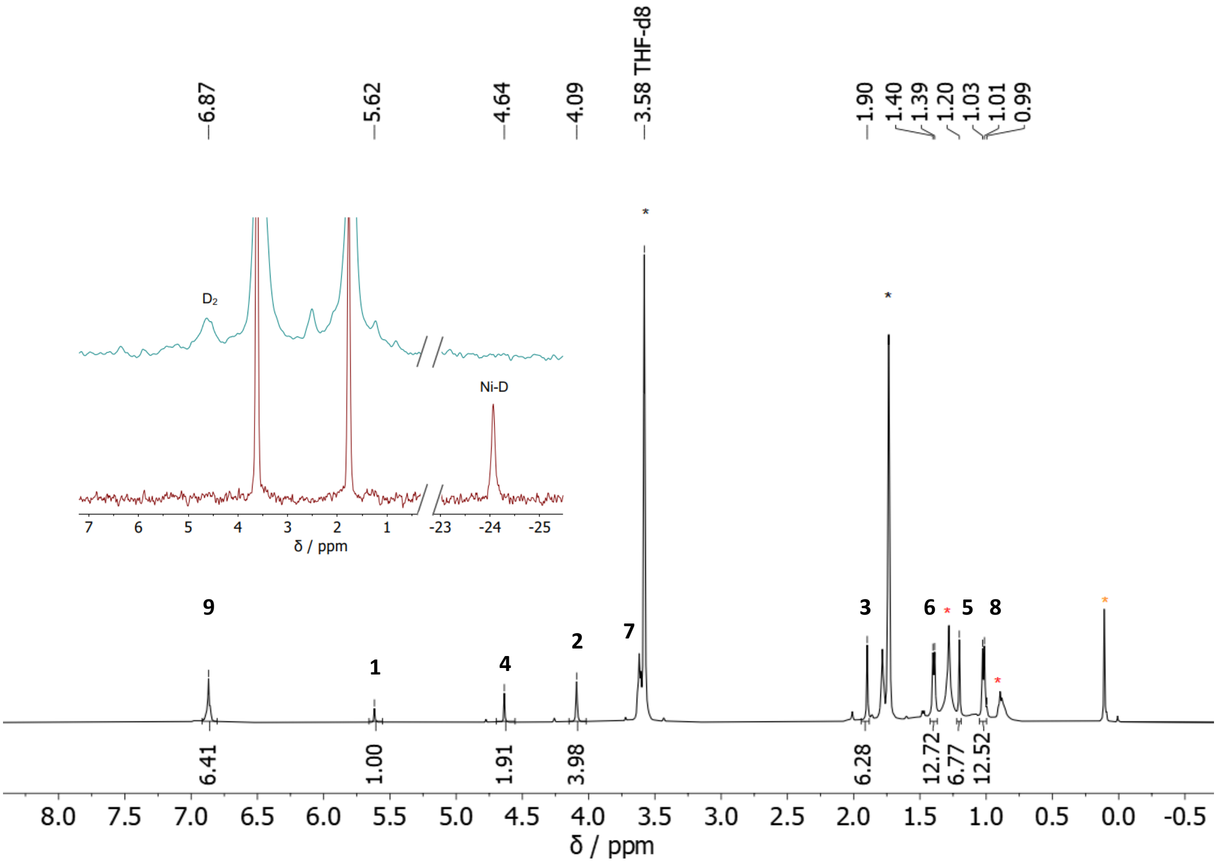

Figure S1. ^{1}H NMR spectrum (500 MHz, 238 K) of complex $\mathbf{2}^{\mathbf{K}}$ in THF-d_8. Solvent signals are marked with an asterisk (*; black for THF, red for hexanes, orange for grease). The inset shows the ^{2}H NMR spectrum of $[KL(Ni\text{-}D)_2]$ ($\mathbf{1}^{\mathbf{K\text{-}D2}}$; bottom) and after addition of S_8 (top), evidencing the liberation of D_2.

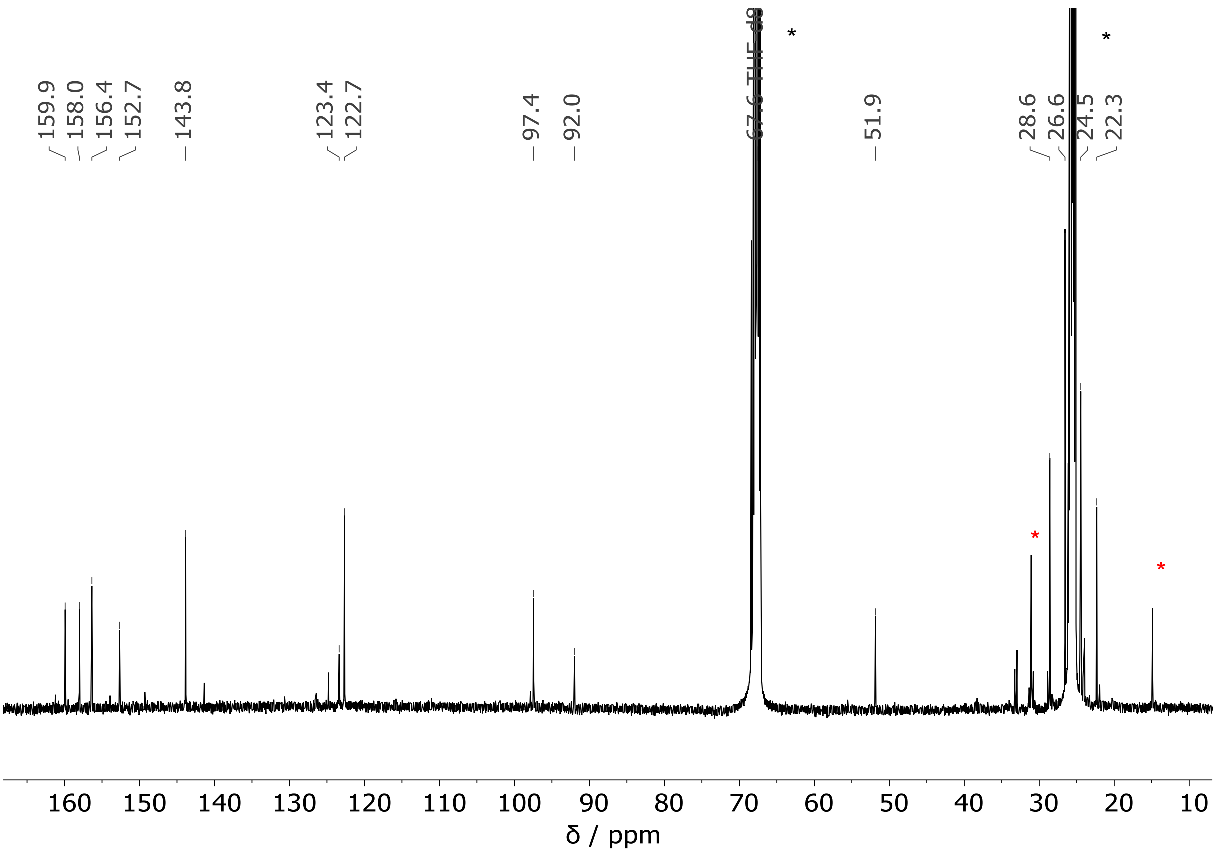

Figure S2. $^{13}C\{^1H\}$ NMR spectrum (126 MHz, 238 K) of complex **2**^{K} in THF-d_8. Solvent signals are marked with an asterisk (*).

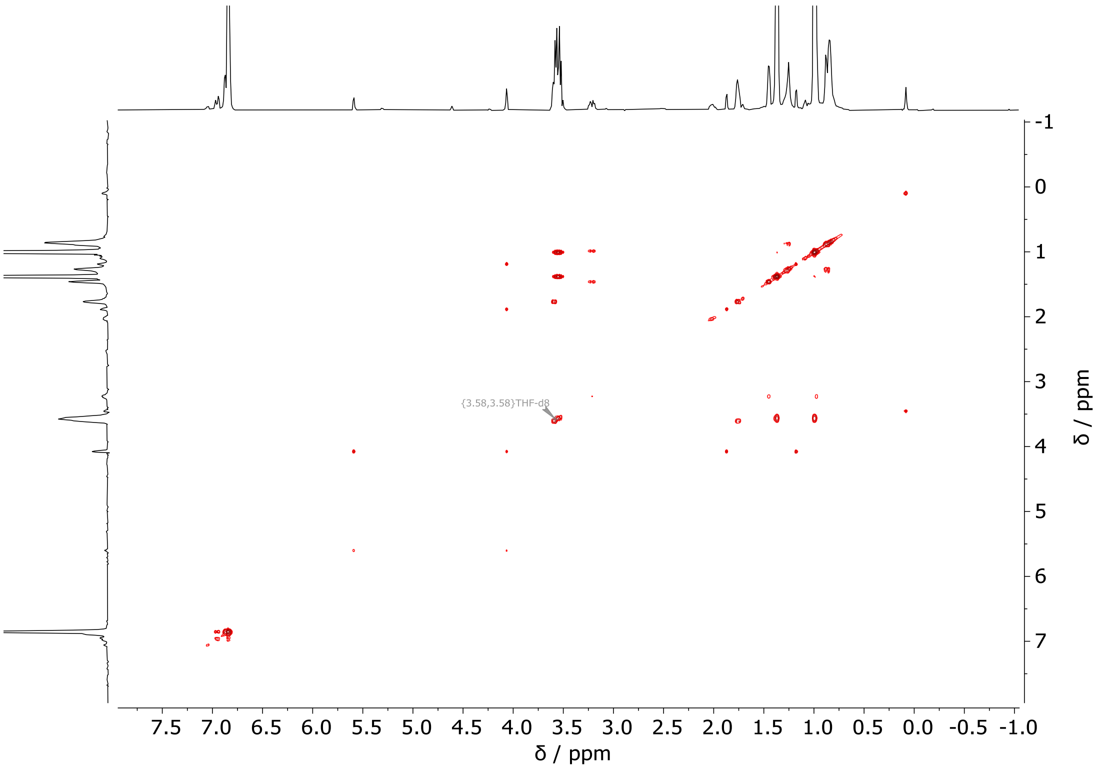

Figure S3. ^{1}H-^{1}H COSY spectrum (500 MHz, 238 K) of complex **2**$^{\mathbf{K}}$ in THF-d$_8$.

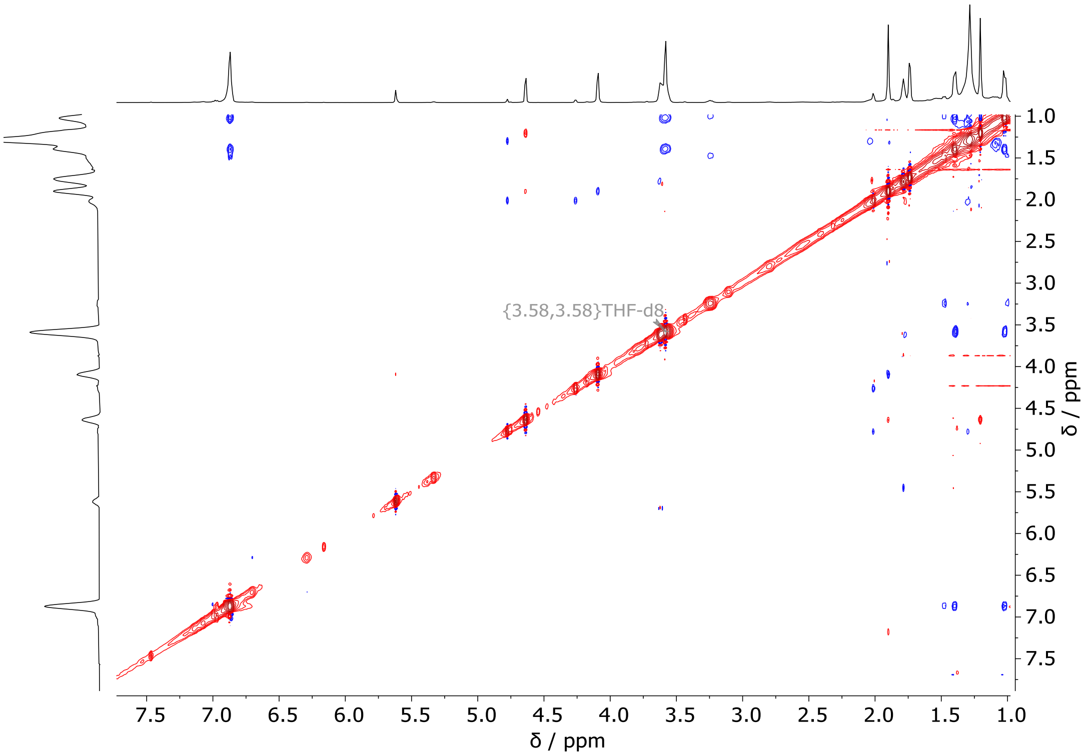

Figure S4. ^{1}H-^{1}H NOESY (500 MHz, 238 K) spectrum of complex **2**$^{\mathbf{K}}$ in THF-d$_8$.

A.2 UV-vis spectra

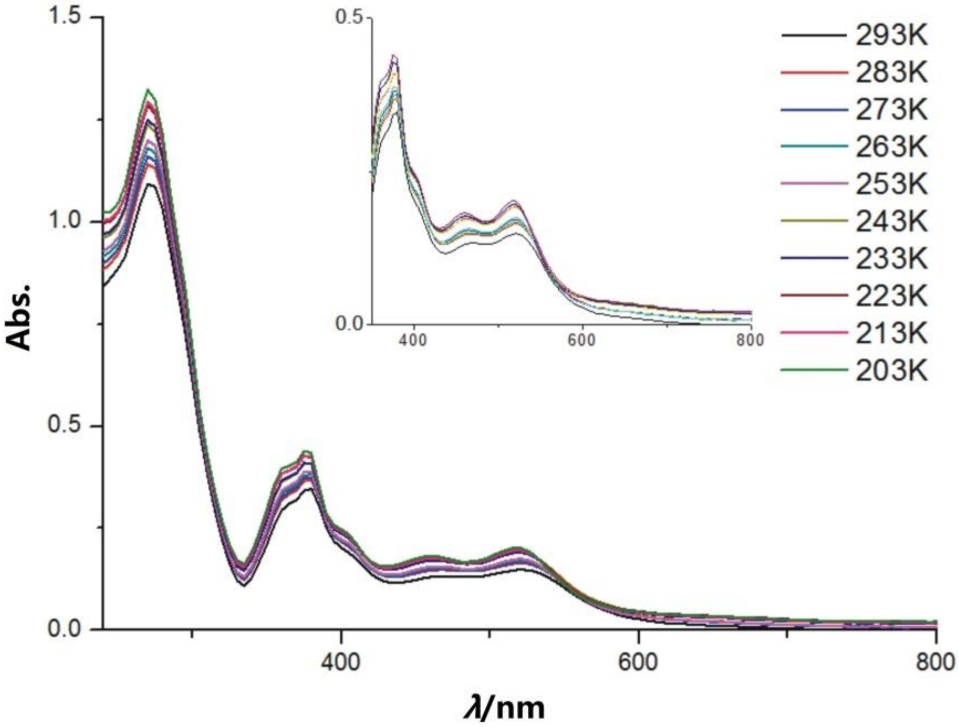

Figure S5. Variable temperature UV/vis spectra of complex **2**^{K} in THF solution in the temperature range from 293 K to 203 K.

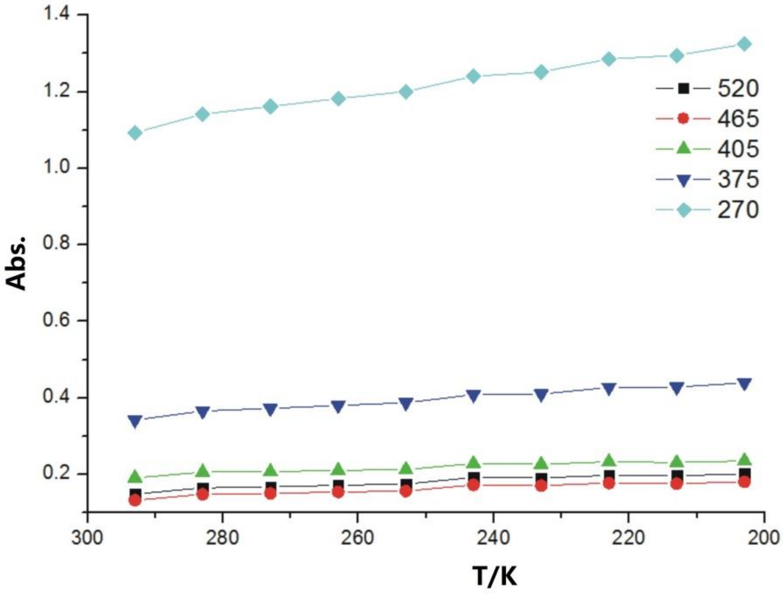

Figure S6. UV-vis absorption changes of a THF solution of complex **2**^{K} in the temperature range from 293 K to 203 K at selected wavelengths.

A.3 IR spectrum

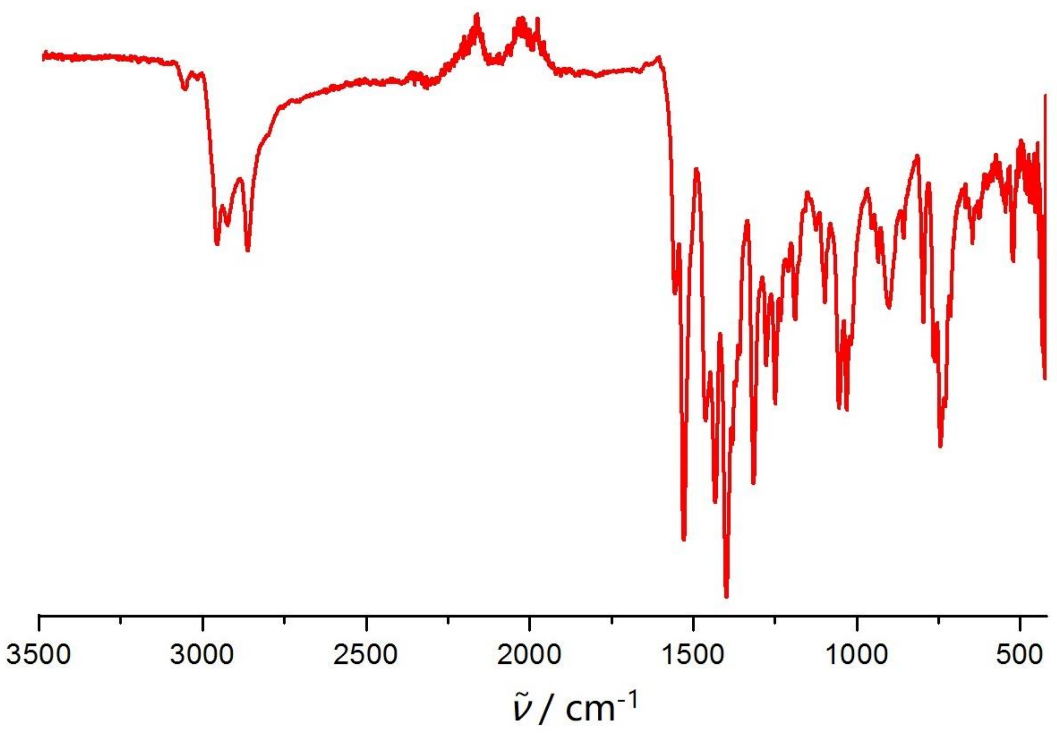

Figure S7. ATR IR spectrum of crystalline material of complex **2**^{K}.

A.4 CV measurements

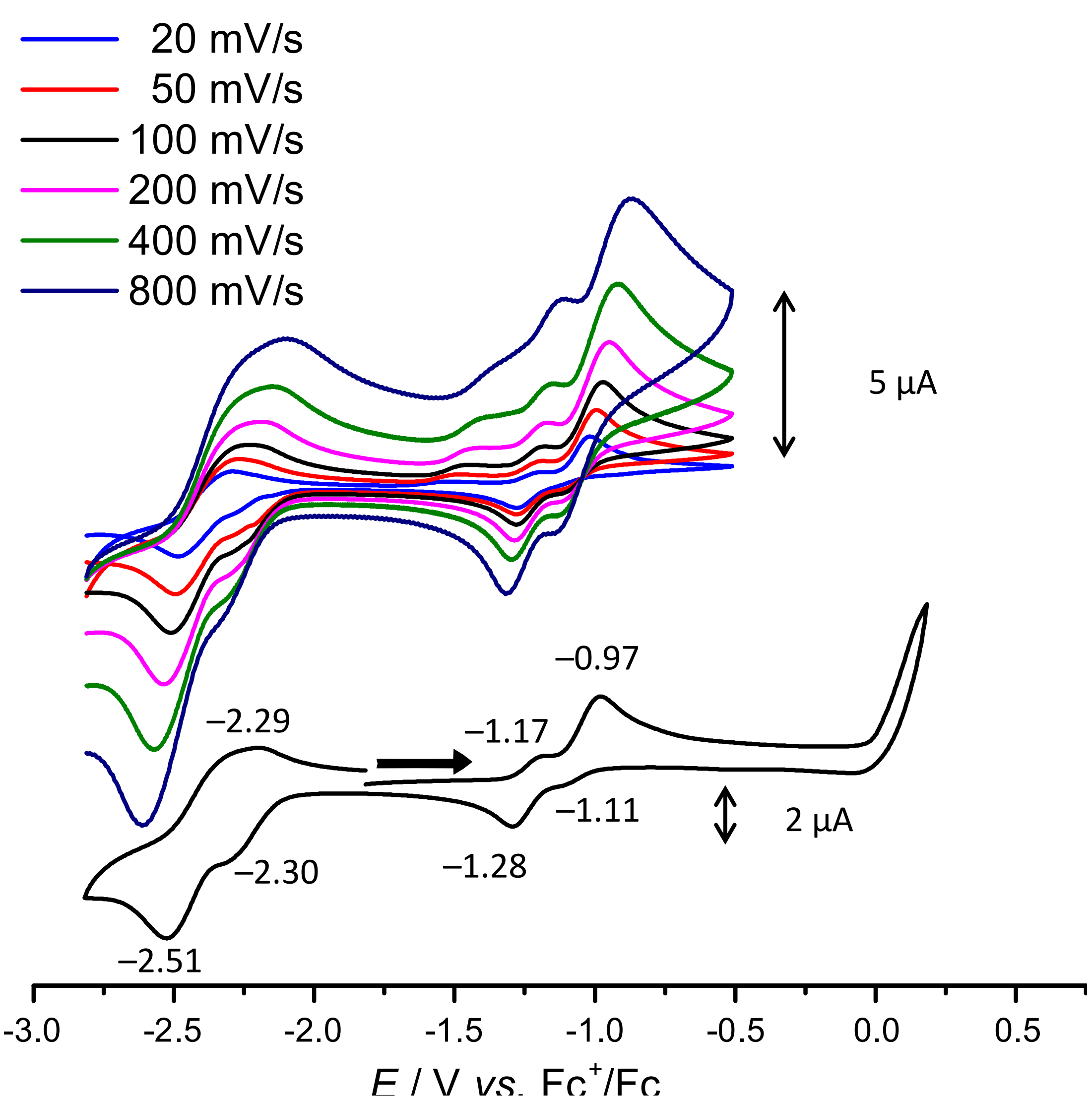

Figure S8. CV of **2**^{K} in THF at rt, with NBu_4PF_6 as supporting electrolyte (0.1 M) at different scan rates. The lower CV (100 mV/s) shows the 2nd scan.

B Complexes 3^K and 3^{Na}

As described in the main manuscript, complex **3**^{K} can be obtained by reacting excess PPh_3 with **2**^{K} in THF (*Method A*) or by reacting S=PMe_3 with **1**^{K} in THF (*Method B*). Similarly, **3**^{Na} is obtained from the reaction of S=PMe_3 with **1**^{Na}.

Alternatively, complex **3**^{K} can be obtained by reduction of the radical complex **6** as well as be deprotonation of the μ-hydrosulfido complex **5** according to the following protocols:

(a) **6** (33 mg, 0.04 mmol, 1 equiv.) and excess potassium were suspended in 2 mL of THF at room temperature, and a color changes from brown to red occurred immediately. The mixture was stirred for 2 hours, all solid components were then removed by filtration, and the solvent of the remaining solution of the product **3**^{K} was removed under reduced pressure. The product **3**^{K} was identified by ^1H NMR spectroscopy.

(b) **5** (16.5 mg, 0.02 mmol, 1 equiv.) and excess potassium hydride were suspended in 2 mL of THF at room temperature. Gas (H_2) evolution was observed, and a color change from brown to red occurred within 2 hours. The mixture was stirred for 4 hours, and all solid components were then removed by filtration. Recrystallization of the crude product by layering hexane on the THF solution at –30°C yielded red block crystals of **3**^{K} (yield: 60%).

B.1 NMR spectra

Monitoring of the reaction of 2^K with PPh_3

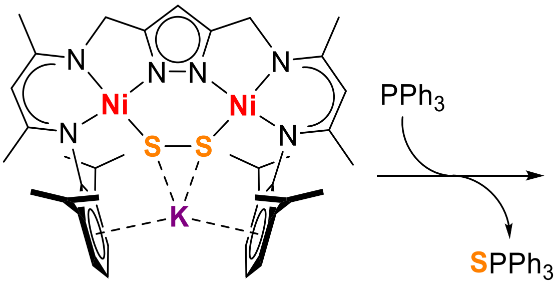

2^K **3^K**

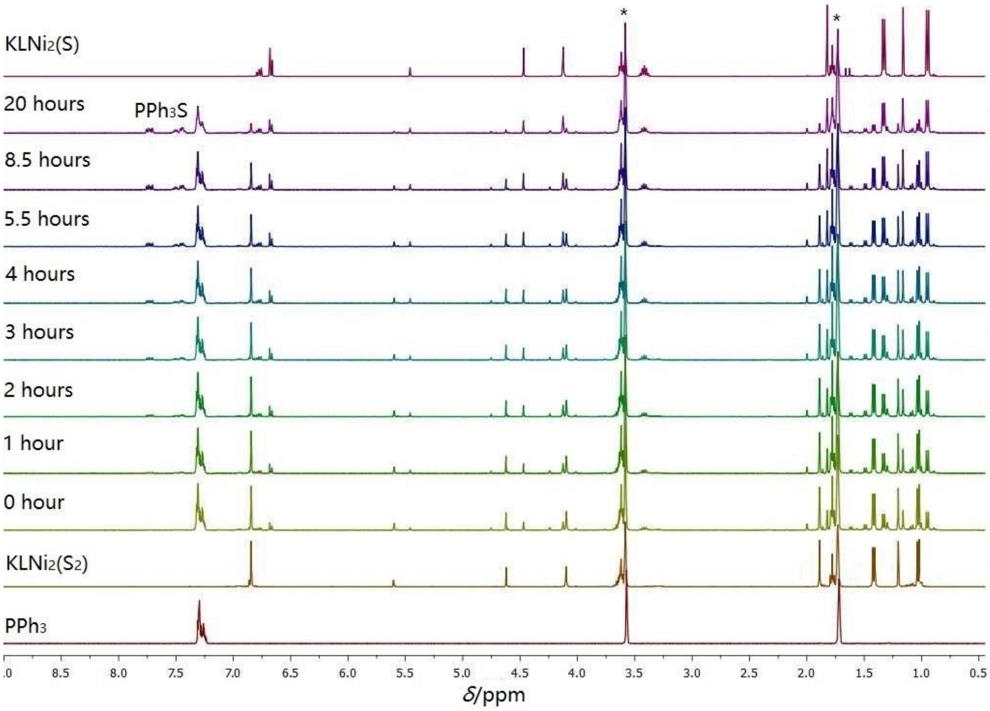

Figure S9. ^{1}H NMR spectroscopic monitoring of the slow conversion of $\mathbf{2^K}$ to $\mathbf{3^K}$ in the presence of PPh_3 at RT in THF-d_8. Residual solvents are marked with an asterisk (*).

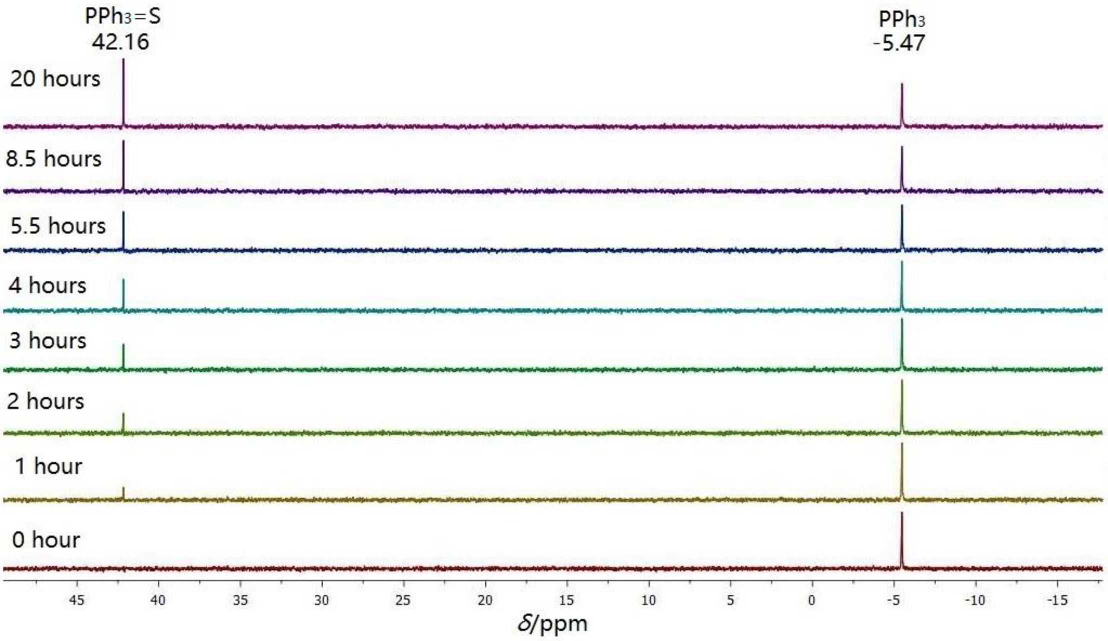

Figure S10. ^{31}P NMR spectroscopic monitoring of the slow conversion of PPh_3 to $SPPh_3$ at rt in THF-d_8 upon reaction of $\mathbf{2^K}$ to $\mathbf{3^K}$.

Direct synthesis of 3^M from 1^M

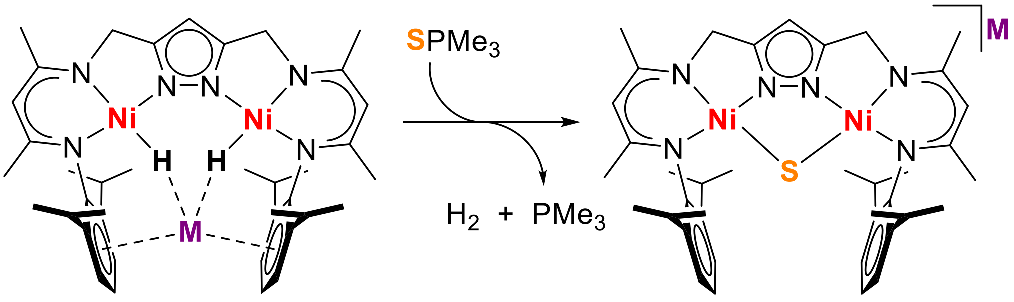

1^M $M^+ = K^+, Na^+$ 3^M

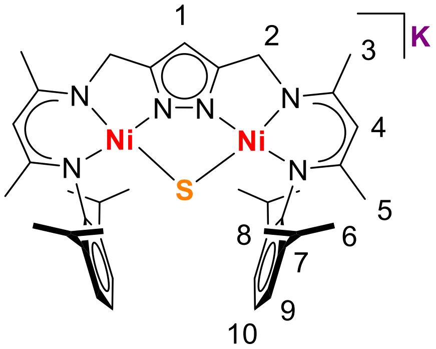

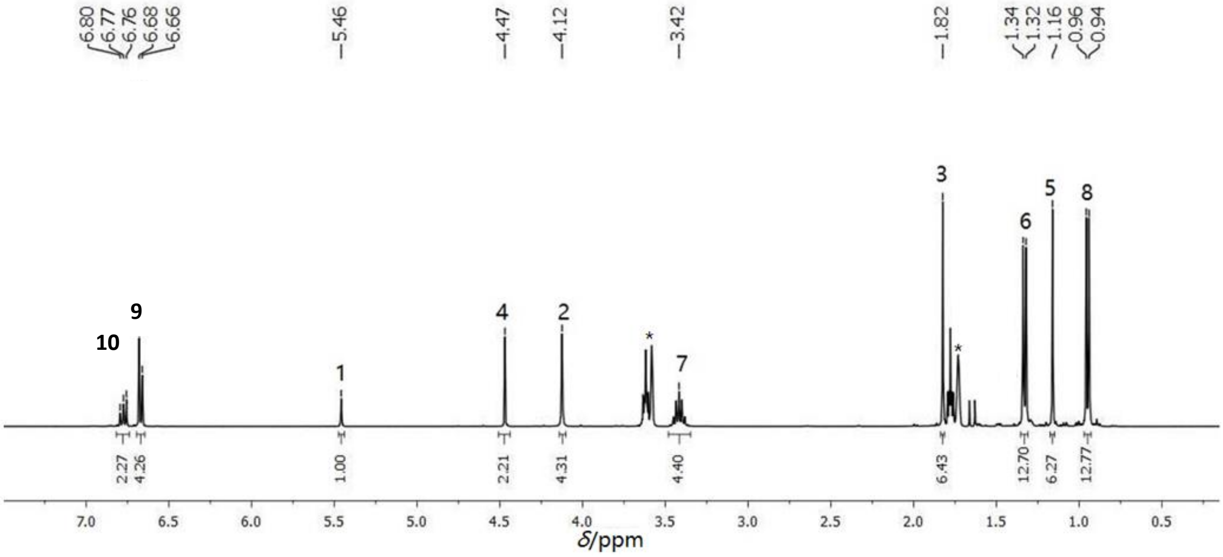

Figure S11. ^{1}H NMR spectrum of complex $\mathbf{3^K}$ in THF-d$_8$. Solvent signals are marked with an asterisk (*).

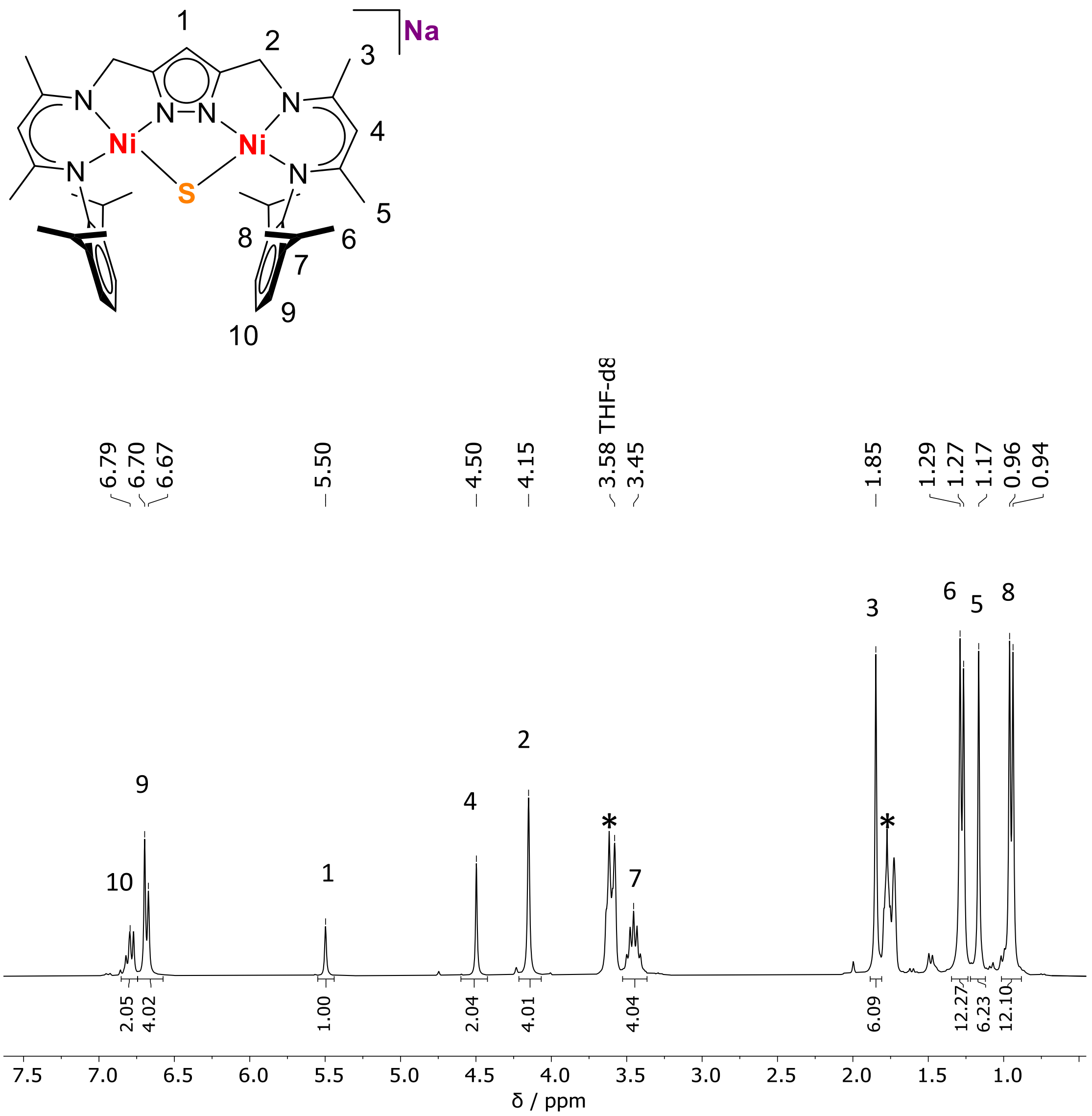

Figure S12. ^{1}H NMR spectrum of complex **3^{Na}** in THF-d$_8$. Solvent signals are marked with an asterisk (*).

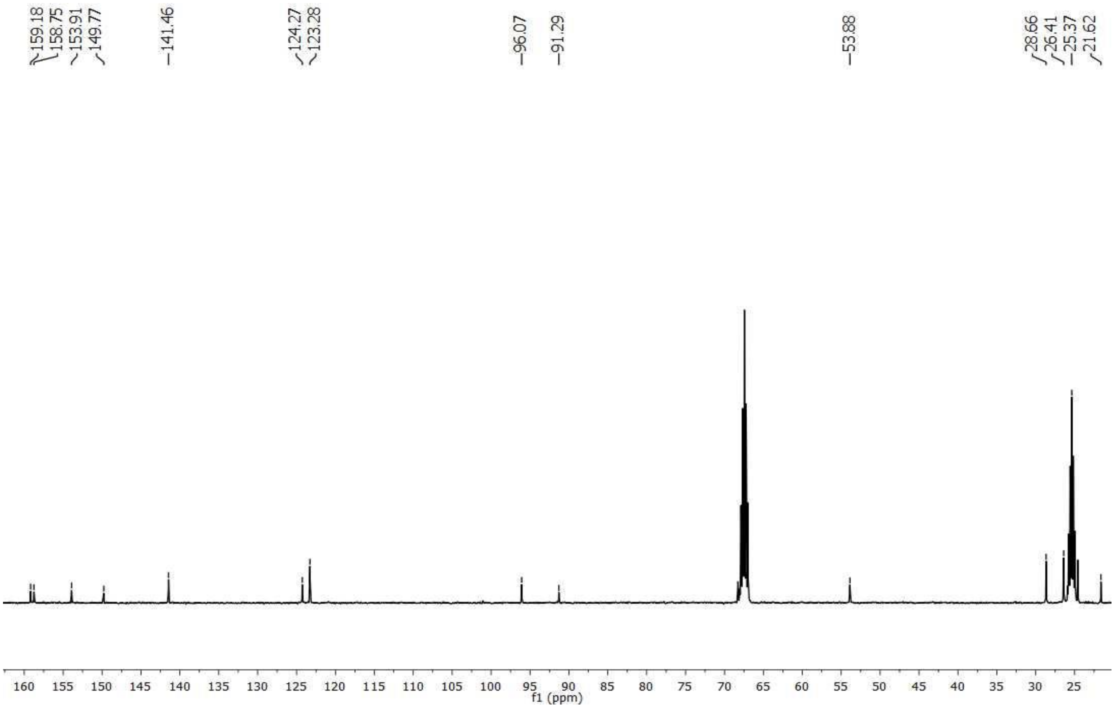

Figure S13. $^{13}C\{^1H\}$ NMR spectrum of complex $\mathbf{3^{Na}}$ in THF-d$_8$.

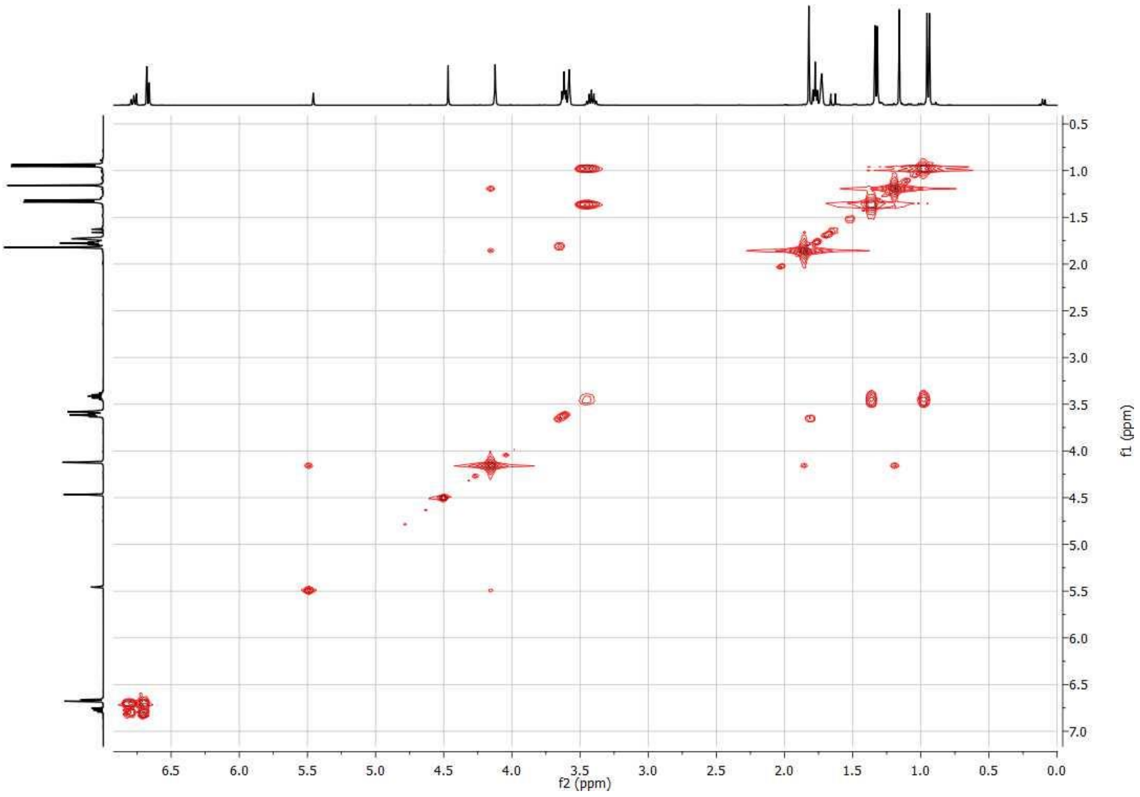

Figure S14. ^1H-^1H COSY (400 MHz) spectrum of $\mathbf{3^{Na}}$ in THF-d$_8$.

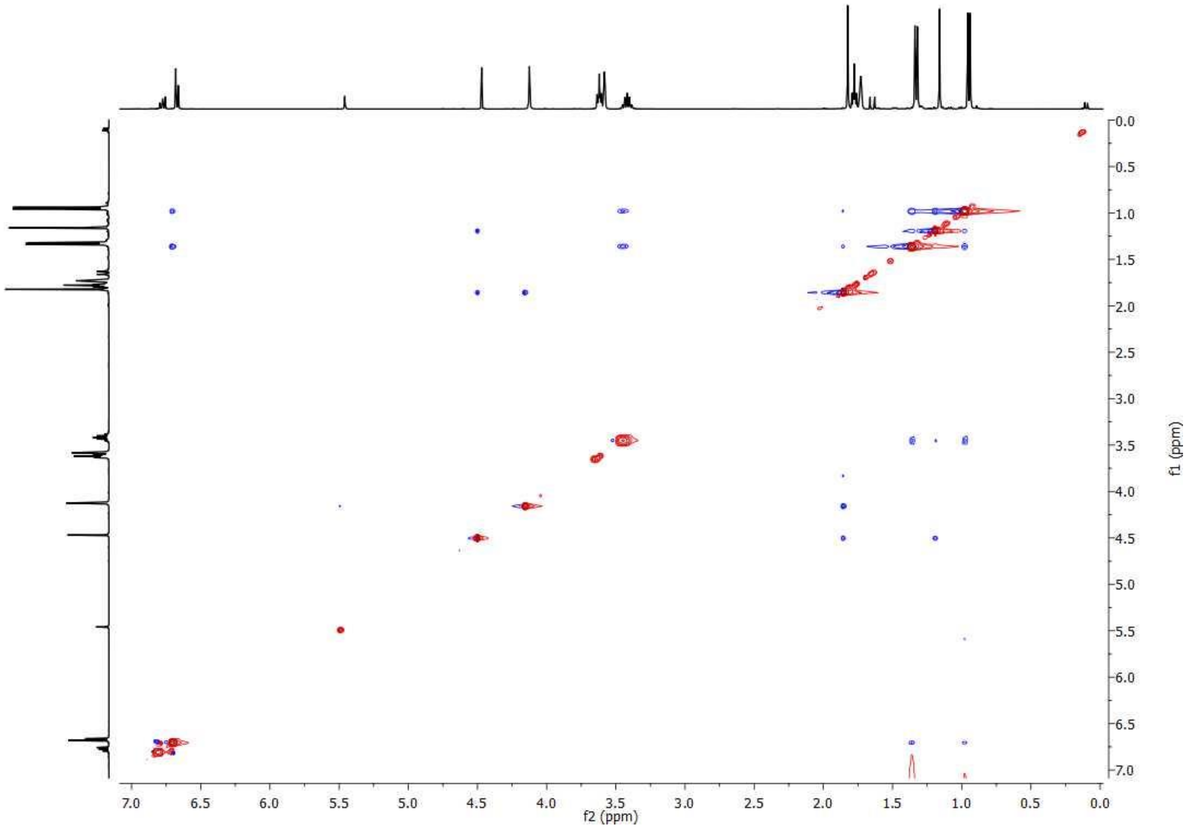

Figure S15. ^{1}H-^{1}H NOESY (400 MHz) spectrum of $\mathbf{3^{Na}}$ in THF-d_8.

B.2 IR spectrum

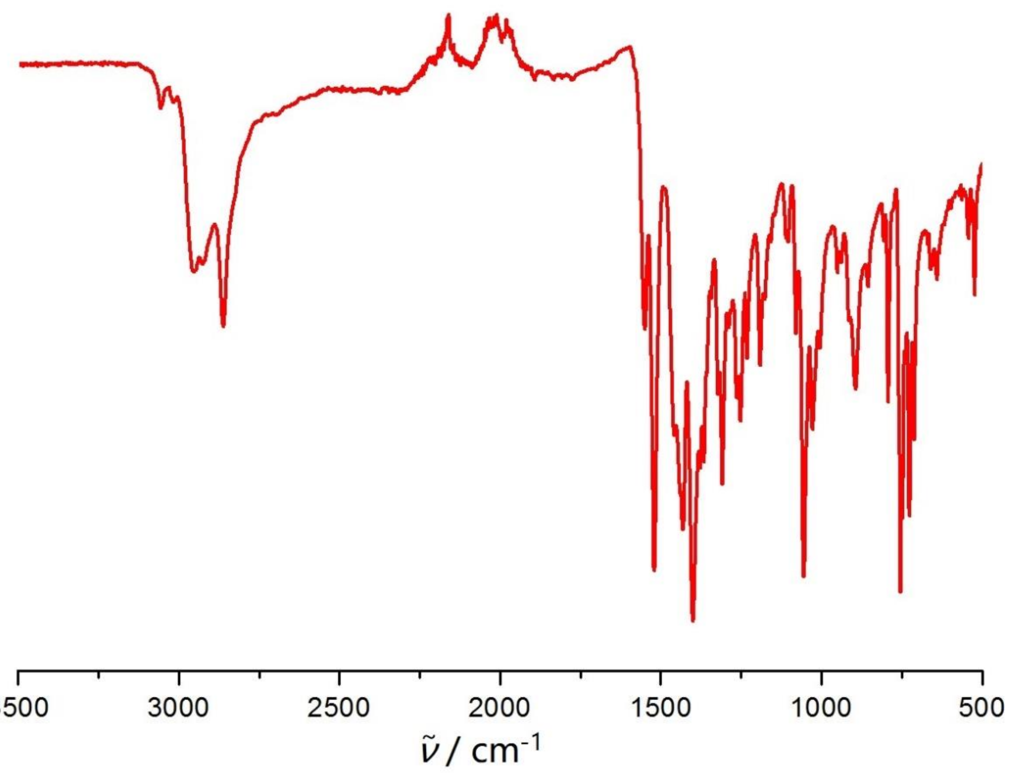

Figure S16. ATR IR spectrum of crystalline material of complex **3**^{K}.

B.3 ESI mass spectrum

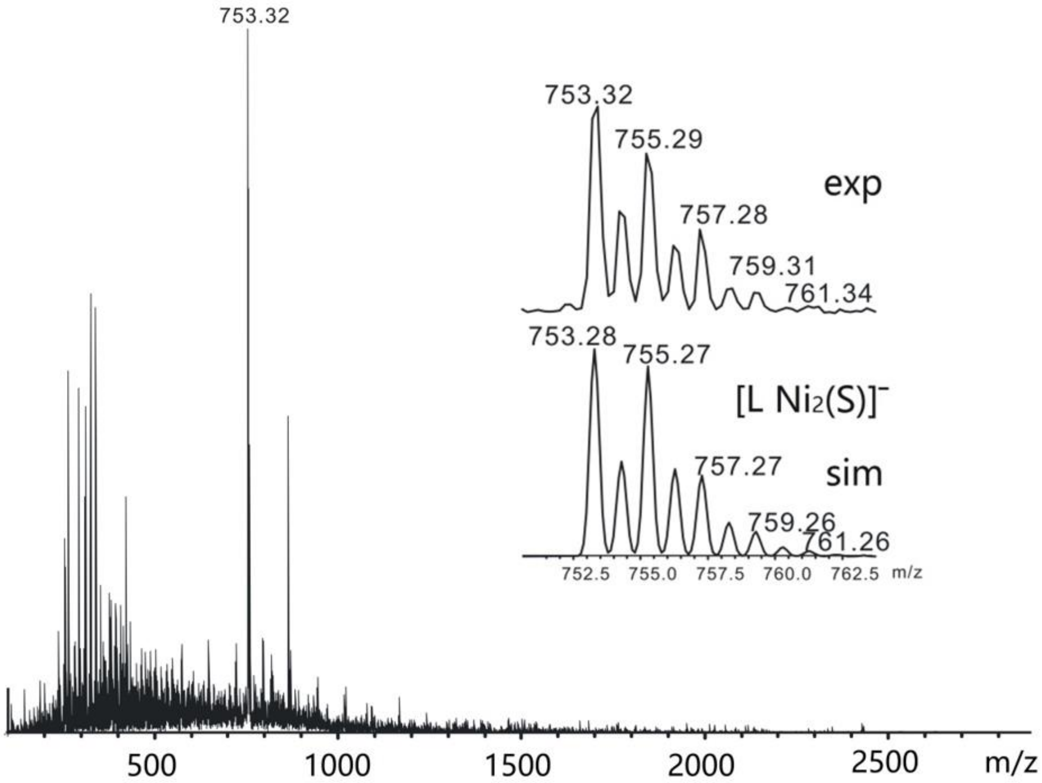

Figure S17. ESI-MS(-) spectrum of $\mathbf{3}^{K}$ in THF. The inset shows the experimental (top) and simulated (bottom) isotopic distribution pattern for the peak pattern around *m/z* = 753.32 characteristic for the ion $[LNi_2S]^-$.

B.4 UV-vis spectra

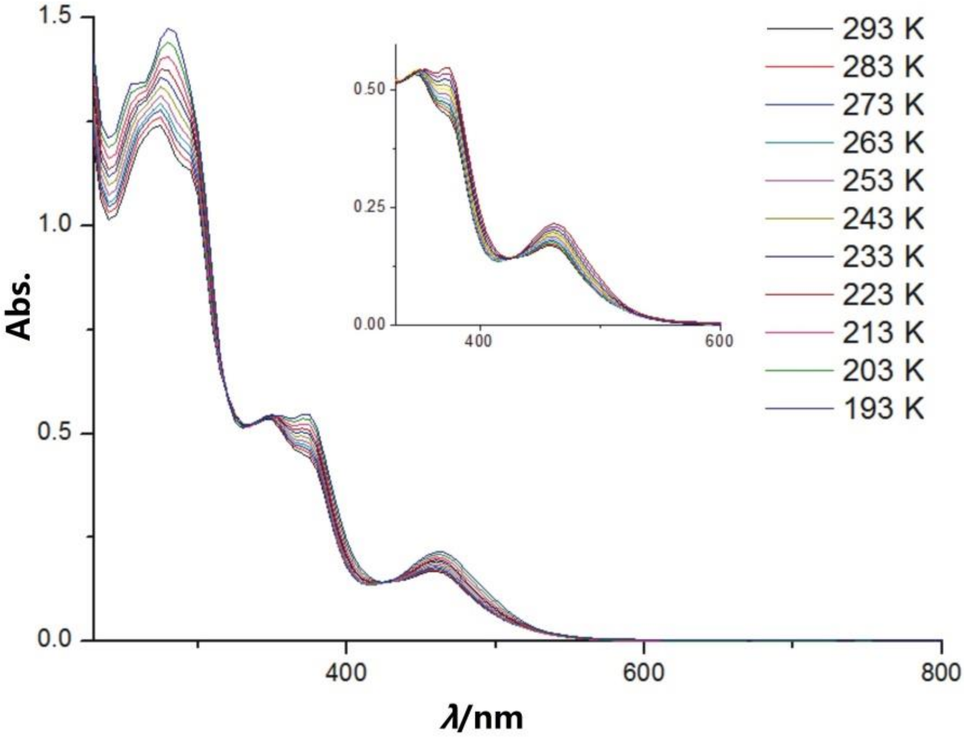

Figure S18. Variable temperature UV/vis spectra of complex **3^K^** in THF solution in the temperature range from 293 K to 193 K.

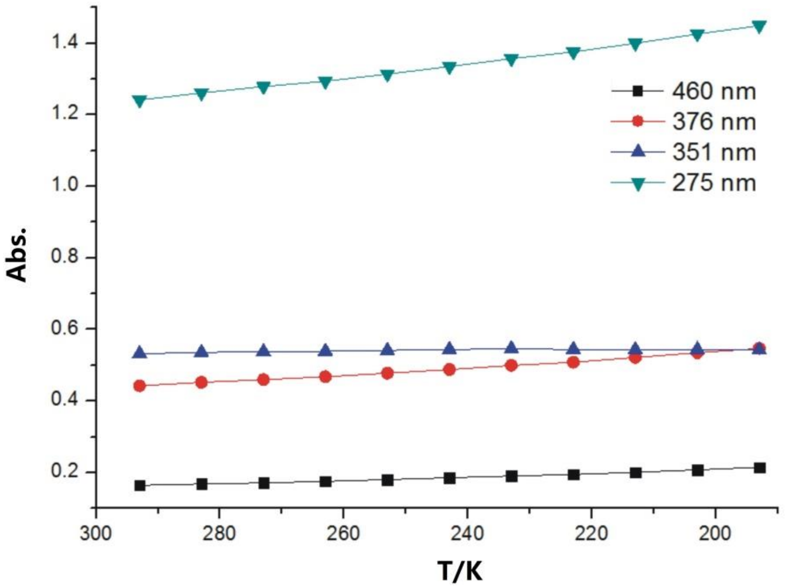

Figure S19. UV/vis absorption changes of a THF solution of complex **3^K^** in the temperature range from 293 K to 193 K at selected wavelengths.

B.5 Titration experiments

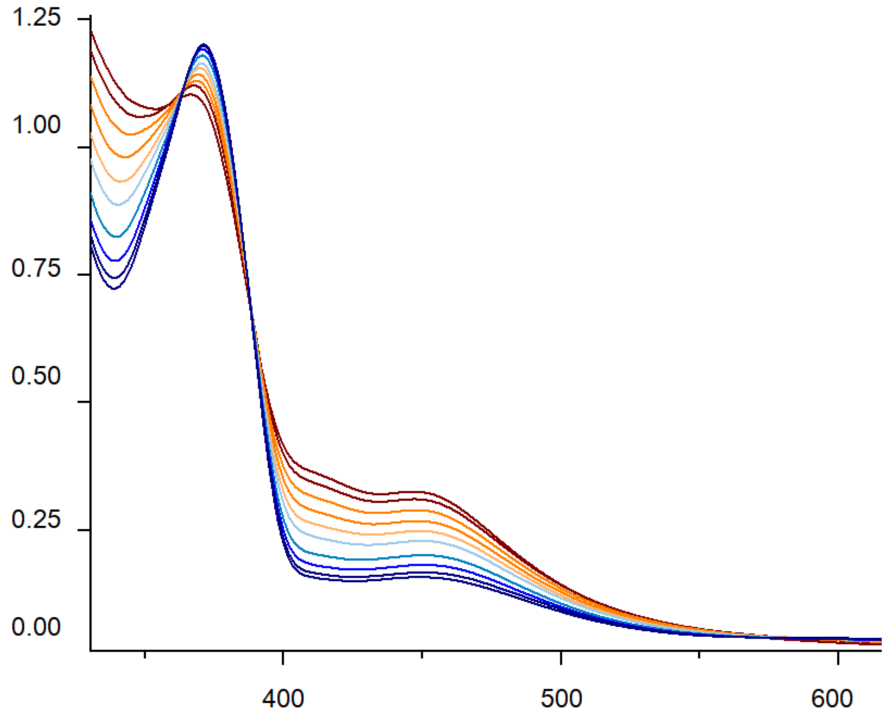

Figure S20. UV-vis titration of complex $\mathbf{3}^{\mathbf{Na}}$ (red spectrum) in THF at rt with benzoic acid (pK_a = 25.11 in THF)[1] in the 270-600 nm range. Full conversion to **5** occurs upon addition of 1 equiv. of acid. Three Isosbestic points are observed at 361, 388 and 580 nm. Since the band at 458 nm was the most characteristic of $\mathbf{3}^{\mathbf{Na}}$, it was chosen for the following titration experiments.

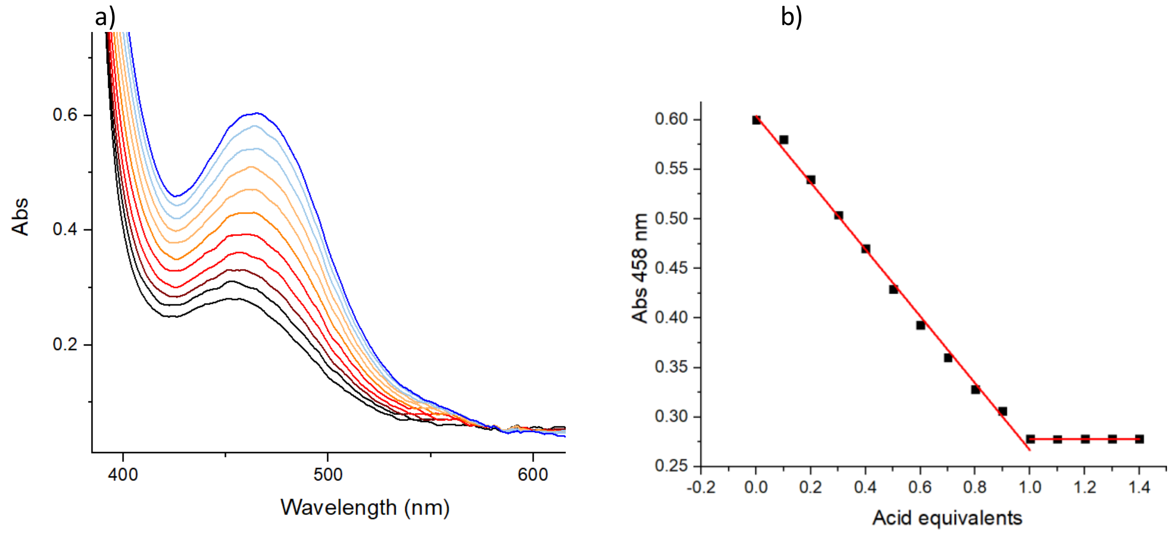

Figure S21. (a) Titration of complex $\mathbf{3}^{\mathbf{Na}}$ in THF solution at rt with phenol (pK_a = 29 in THF).[2] (b) Added equivalents of acid vs. the change in absorption, monitored at 458 nm.

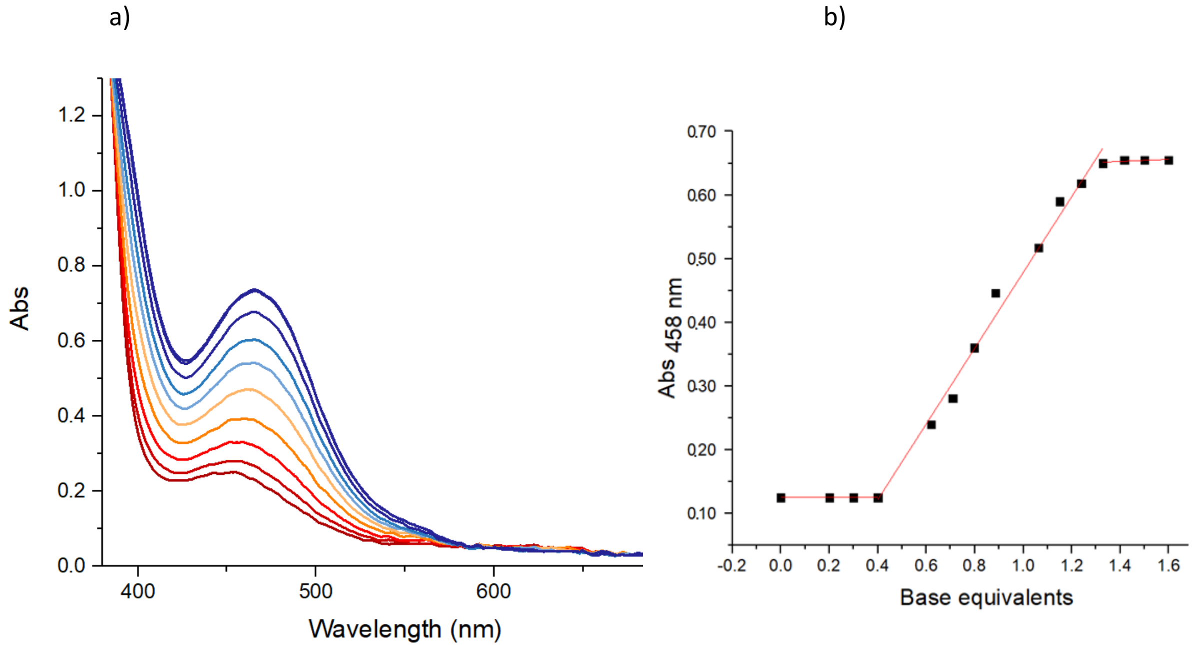

Figure S22. a) UV-vis spectroscopic monitoring of the back titration of complex **5**, obtained by the addition of 1.4 eq of [HLut]BF_4 to $\mathbf{3}^{\mathbf{Na}}$ in thf solution at rt, with P_4 base (pK_a = 33.9 in THF).[13] b) Added equivalents of base vs. the change in absorption, monitored at 458 nm.

B.6 pK_a determination

In order to determine an apparent pK_a value of the hydrosulfide ligand in **5**, the protonation equilibrium was described as the balance between two bases (complex $[LNi_2S]^-$ and the phosphonium ylide, here abbreviated P^-):[3]

$$P^- + [LNi_2(SH)] \rightleftharpoons HP + [LNi_2S]^-$$

Assuming the ion activity *a* being identical to the equilibrium concentrations determined by UV-vis spectroscopy, this implies that the relative basicity can be expressed as:

$$\mathrm{p}K_a(\mathrm{LNi_2(SH)}) = \mathrm{p}K_a(\mathrm{HP}) - \log\frac{[\mathrm{HP}][\mathrm{LNi_2S^-}]}{[\mathrm{LNi_2(SH)}][\mathrm{P^-}]}$$

with:

$$\log\frac{[\mathrm{HP}][\mathrm{LNi_2S^-}]}{[\mathrm{LNi_2(SH)}][\mathrm{P^-}]} = \log(K)$$

Consequently, the equilibrium constant *K* can be determined by plotting $[LNi_2S^-]^2/[LNi_2SH]$ vs. [P] in order to obtain the pK_a of LNi_2SH.

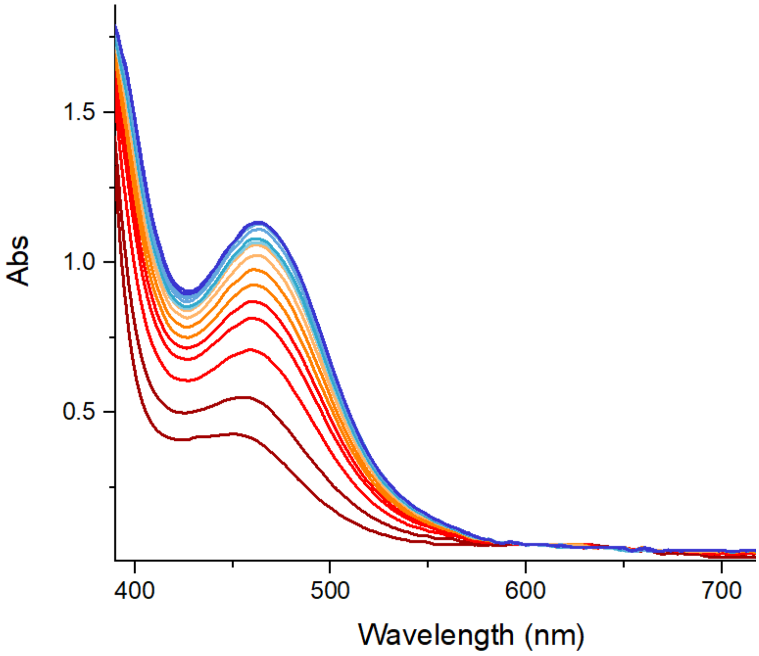

Figure S23. UV-vis spectroscopic monitoring of the back titration of complex **5**, obtained by the addition of 1.0 eq of $[HLut]BF_4$ to $\mathbf{3^{Na}}$ in THF solution at rt, with the phosphonium ylide $MeOCH{=}P(4\text{-}OMe\text{-}C_6H_4)_3$ (pK_a = 31.7 in THF).[4]

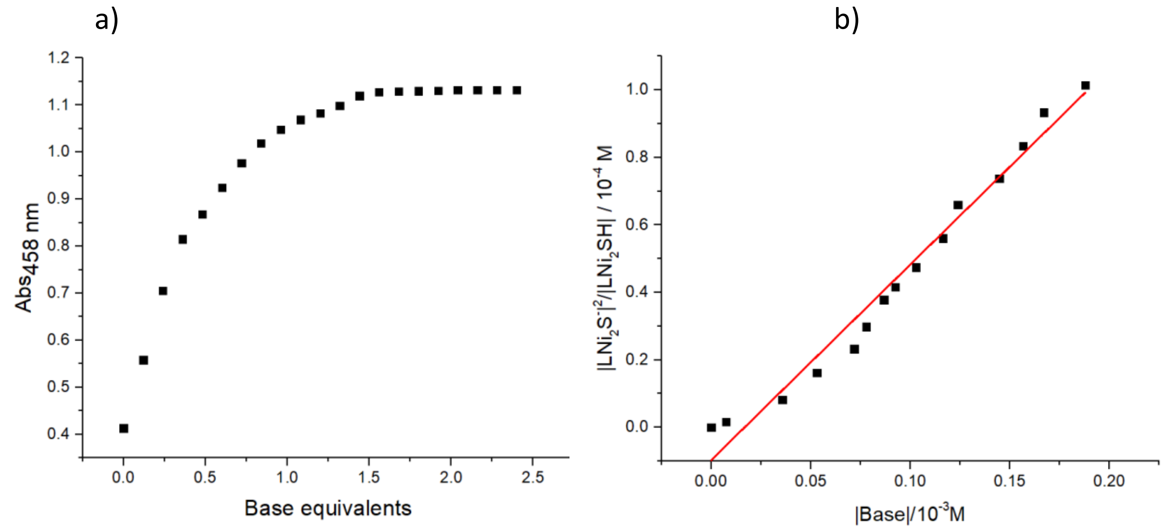

Figure S24. (a) Added equivalents of base vs. the change in absorption, monitored at 458 nm. (b) Plot of $[LNi_2S^{-}]^2/[LNi_2SH]$ vs. $[MeOCH{=}P(4\text{-}OMe\text{-}C_6H_4)_3]$ derived from the data shown in Figures S23 and S24a. The slope gives the equilibrium constant $K = 7.3$.

Based on the results of several independent titration experiments, uncertainties in the slope determination and further error approximations, an overall error of ±0.4 is assumed, thus giving $pK_a(LNi_2SH) = 30.8 \pm 0.4$.

B.7 CV measurements

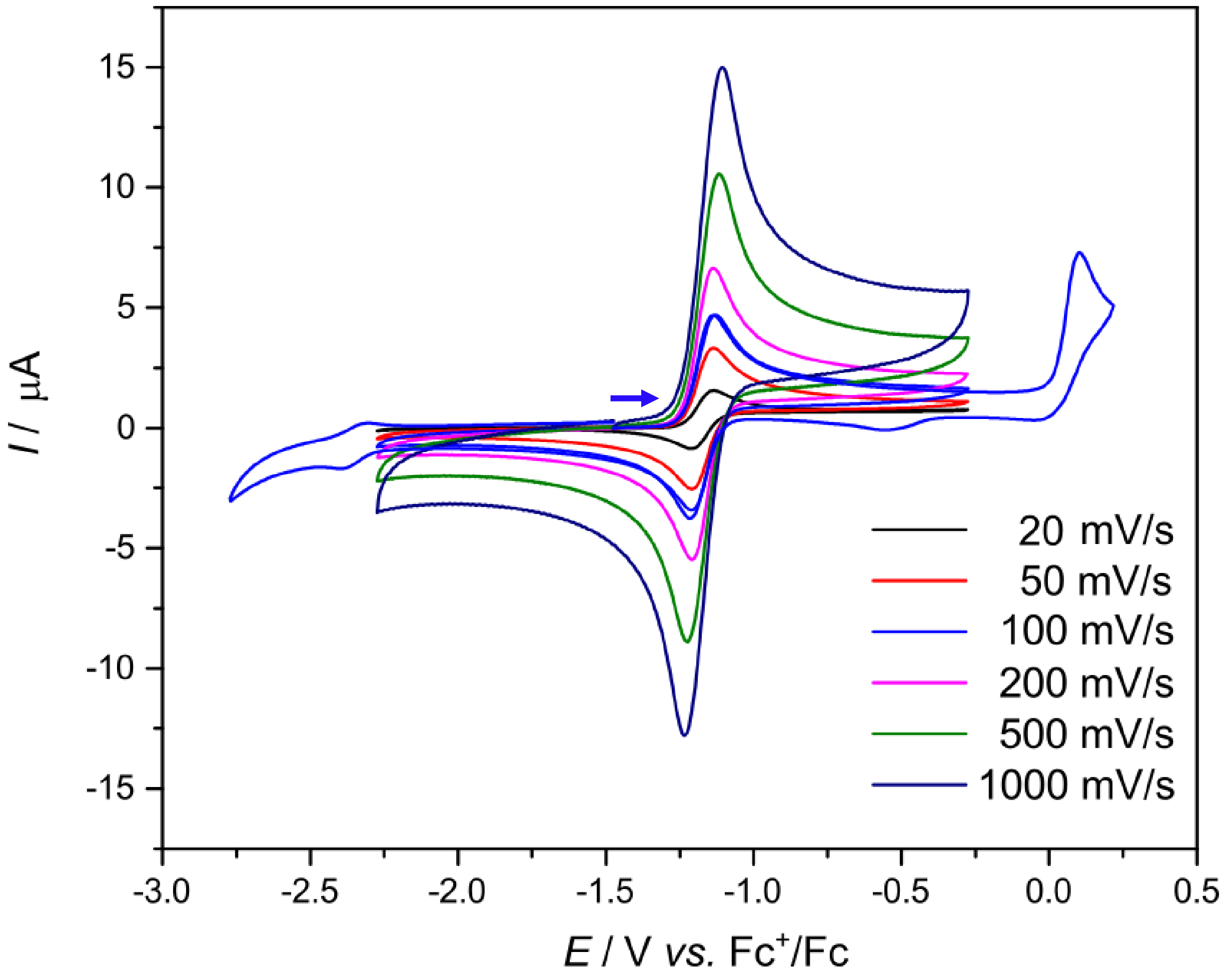

Figure S25. CV of $\mathbf{3}^{Na}$ in THF at rt, with NBu_4PF_6 as supporting electrolyte (0.1 M) at different scan rates. As observed for $\mathbf{3}^{K}$ a reversible oxidation occurs at $E_{1/2}$ = −1.17 V against Fc^+/Fc.

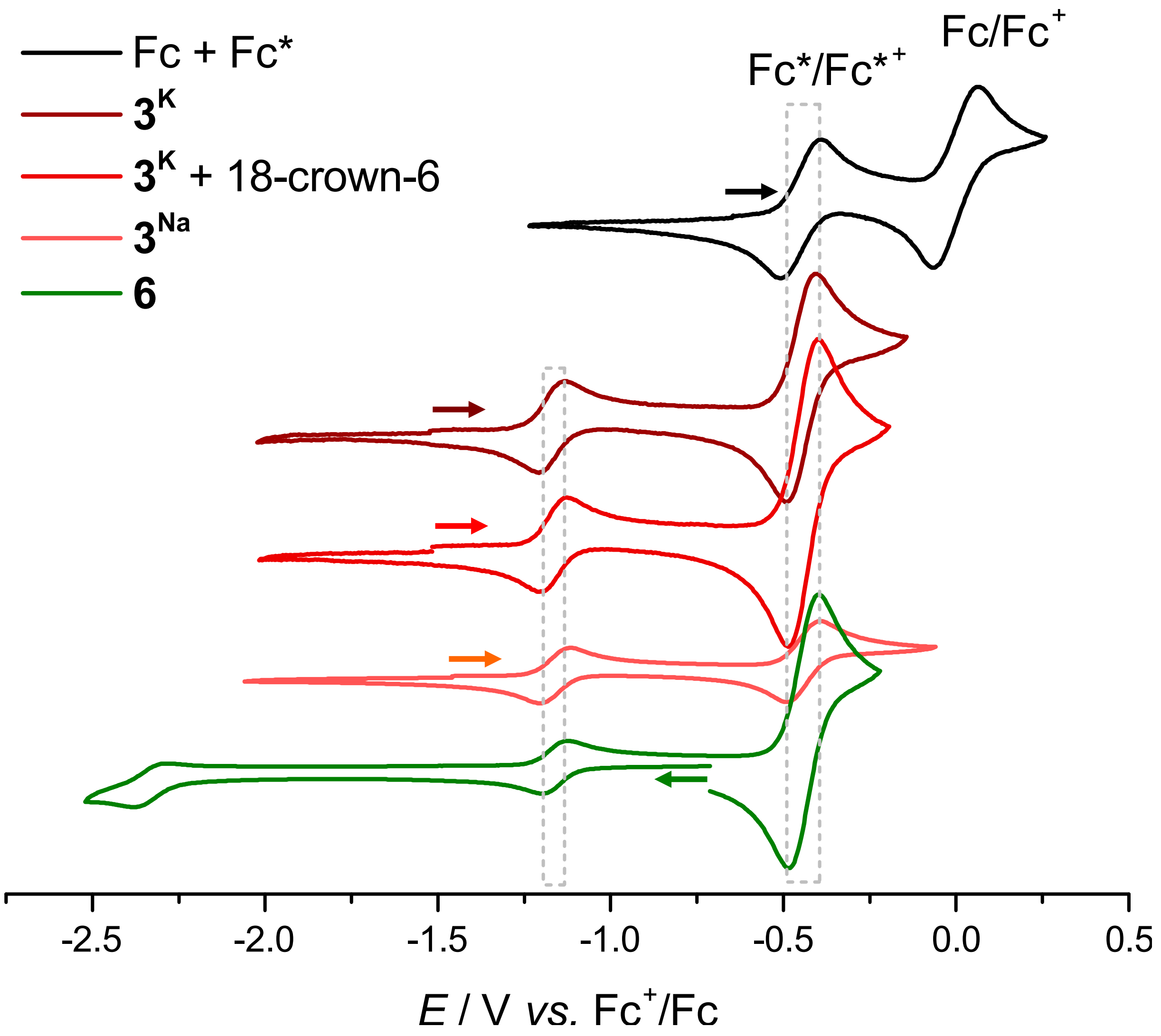

Figure S26. CV of $\mathbf{3^K}$ (bordeaux), of $\mathbf{3^K}$ with 10 equivalents of 18-crown-6 (red), of $\mathbf{3^{Na}}$ (orange) and **6** (green) in THF at rt, with NBu_4PF_6 as supporting electrolyte (0.1 M) at 100 mV/s with decamethylferrocene (Fc^{*+}/Fc^*) as internal standard. The black curve shows a mixture of ferrocene (Fc^+/Fc) and decamethylferrocene (Fc^{*+}/Fc^*) under the same conditions, giving $E_{1/2}(Fc^{*+}/Fc^*)$ = –0.45 V vs. Fc^+/Fc in THF. The vertical dashed lines are provided as a guide to the eye.

B.8 UV-vis SEC of 3^{Na}

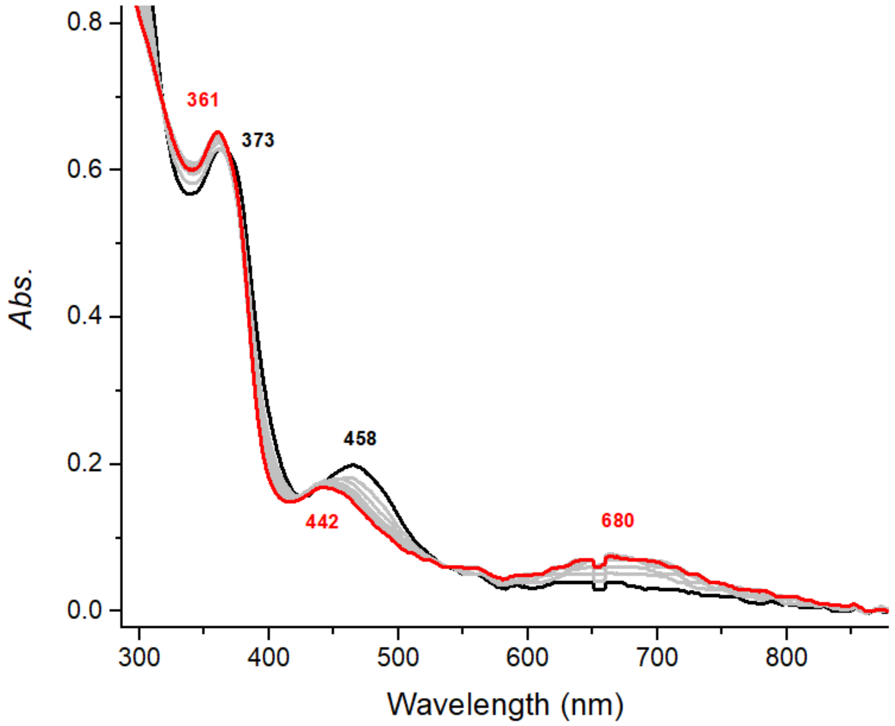

Figure S27. UV-vis spectroelectrochemistry showing the oxidation of $\mathbf{3}^{Na}$ to **6** in THF at rt (at −1.5 V vs Ag wire), with NBu_4PF_6 as supporting electrolyte (0.1 M); the spectroscopic changes are similar to those observed for $\mathbf{3}^{K}$ (Figure 7).

C Complex 4

C.1 NMR spectra

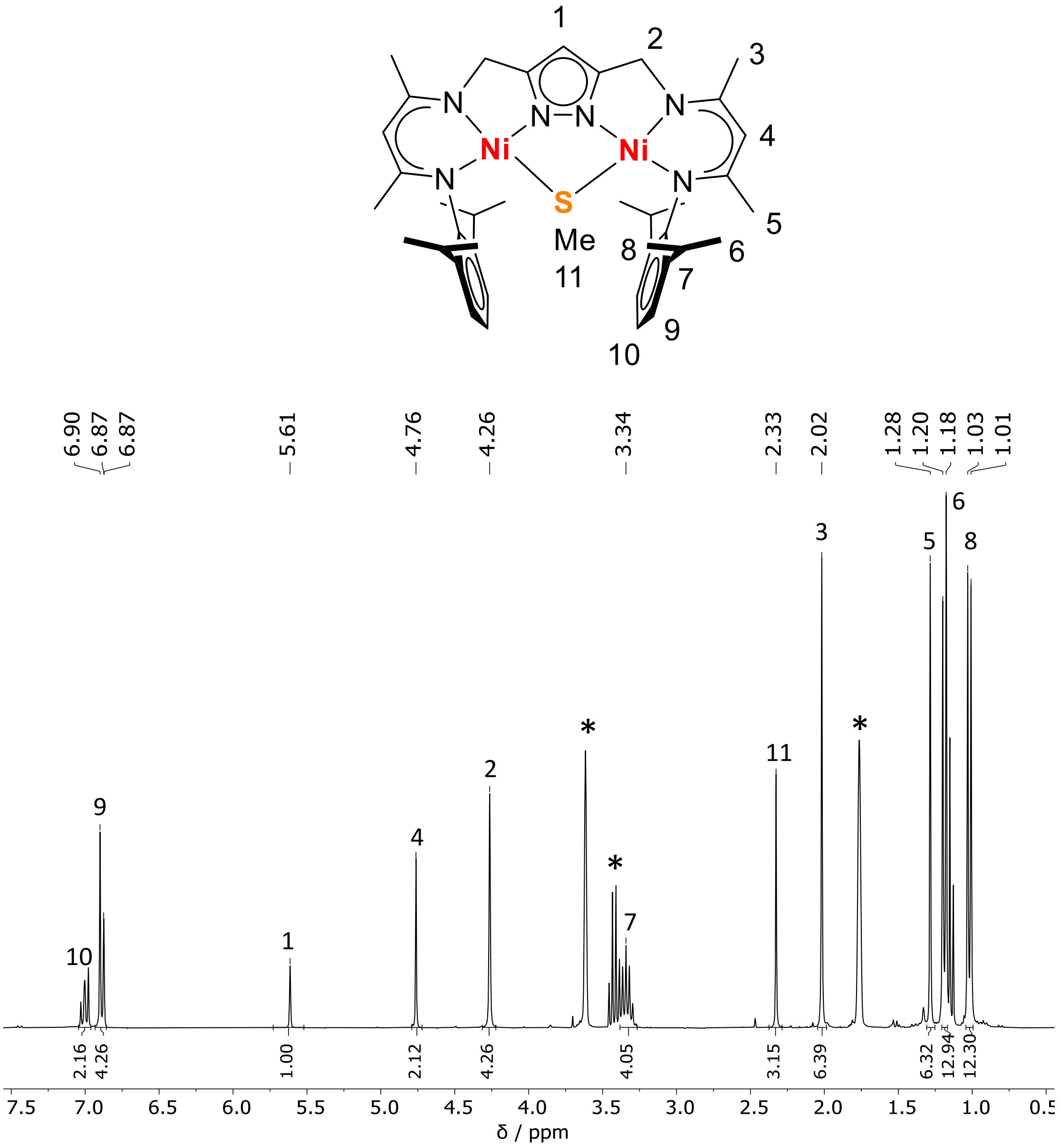

Figure S28. ^{1}H NMR spectrum of complex **4** in THF-d_8.

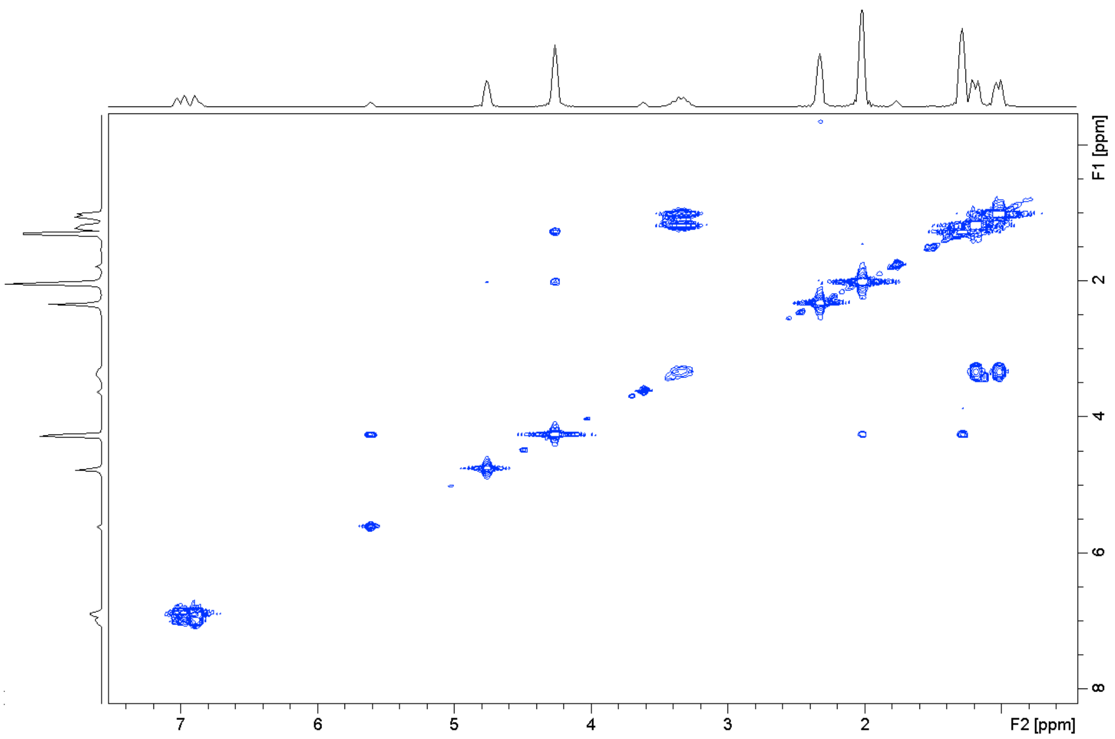

Figure S29. ^1H-^1H COSY (400 MHz) spectrum of **4** in THF-d_8.

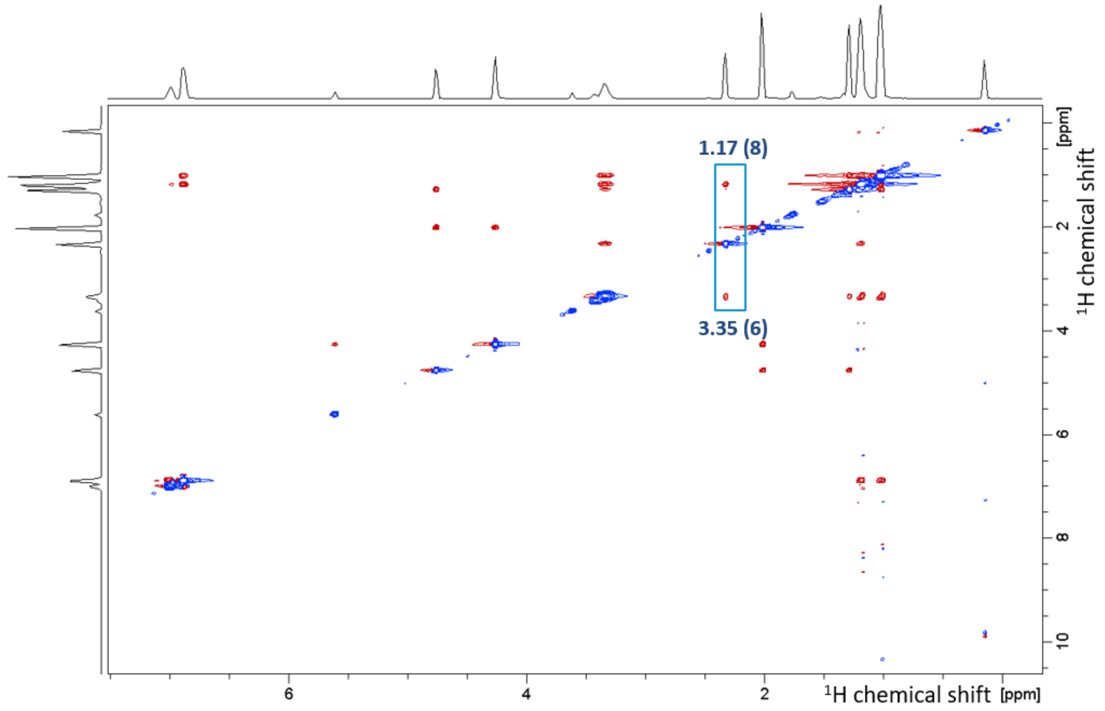

Figure S30. ^1H-^1H NOESY (400 MHz) spectrum of **4** in THF-d_8. The correlation of the signal for the μ-SMe (11) at 2.33 ppm with the Dipp groups (6,8) is highlighted.

C.2 IR spectrum

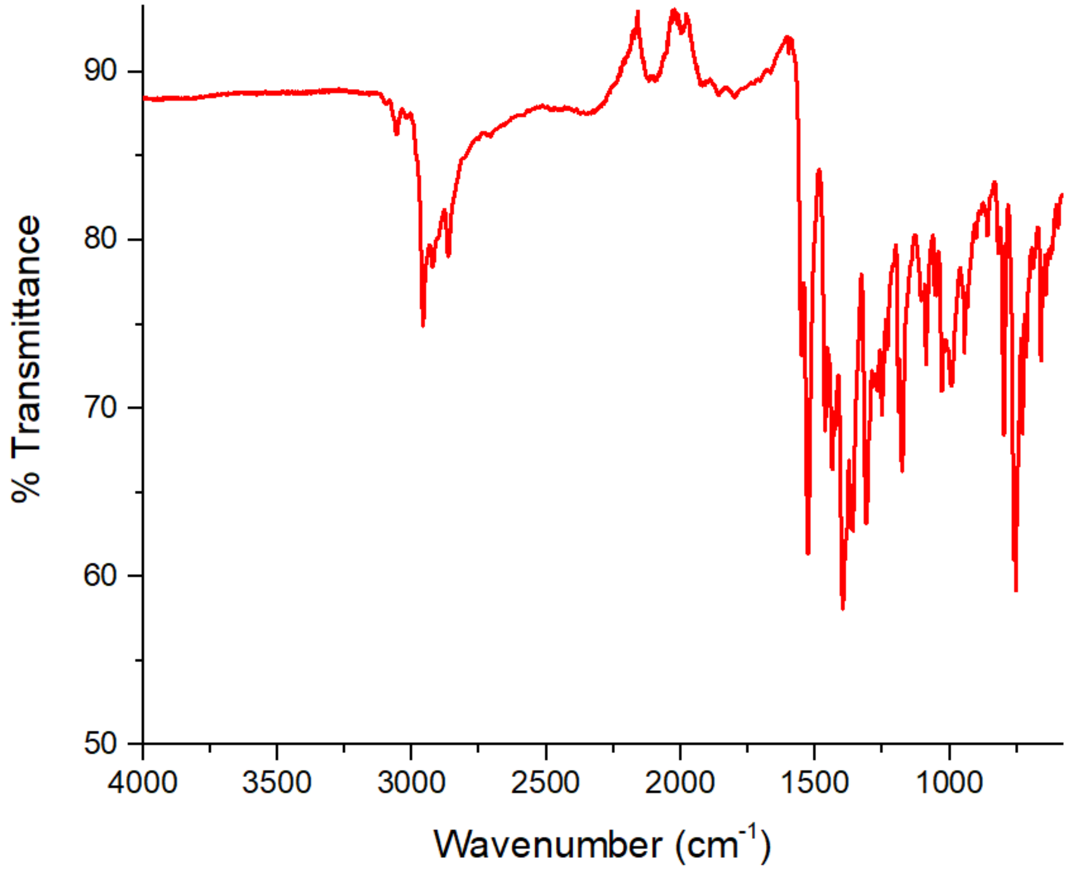

Figure S31. ATR IR spectrum of **4**.

D Complex 5

D.1 NMR spectra

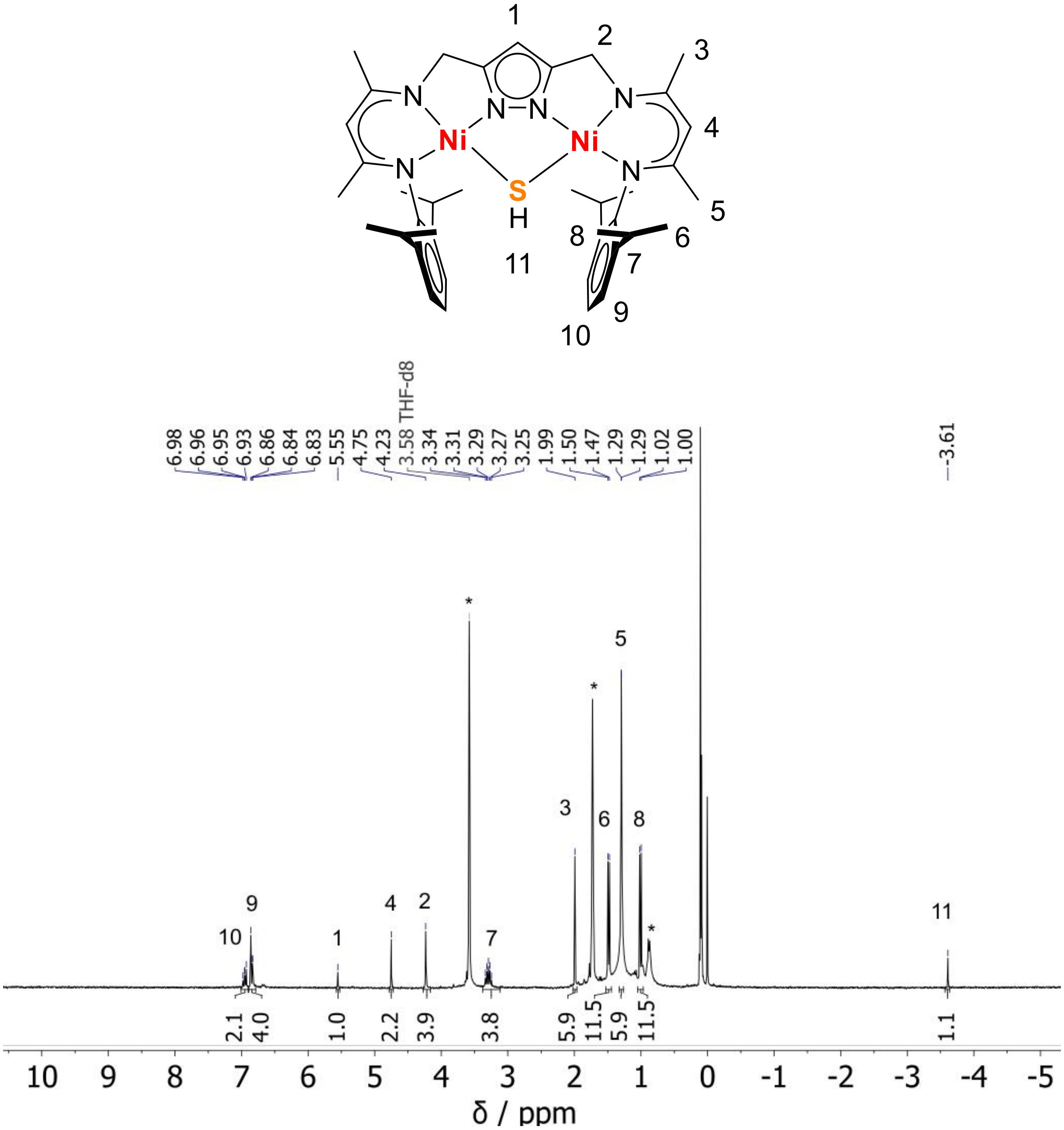

Figure S32. ^{1}H NMR spectrum (300 MHz) of **5** in THF-d_8. Residual solvent signals are indicated with an asterisk (*).

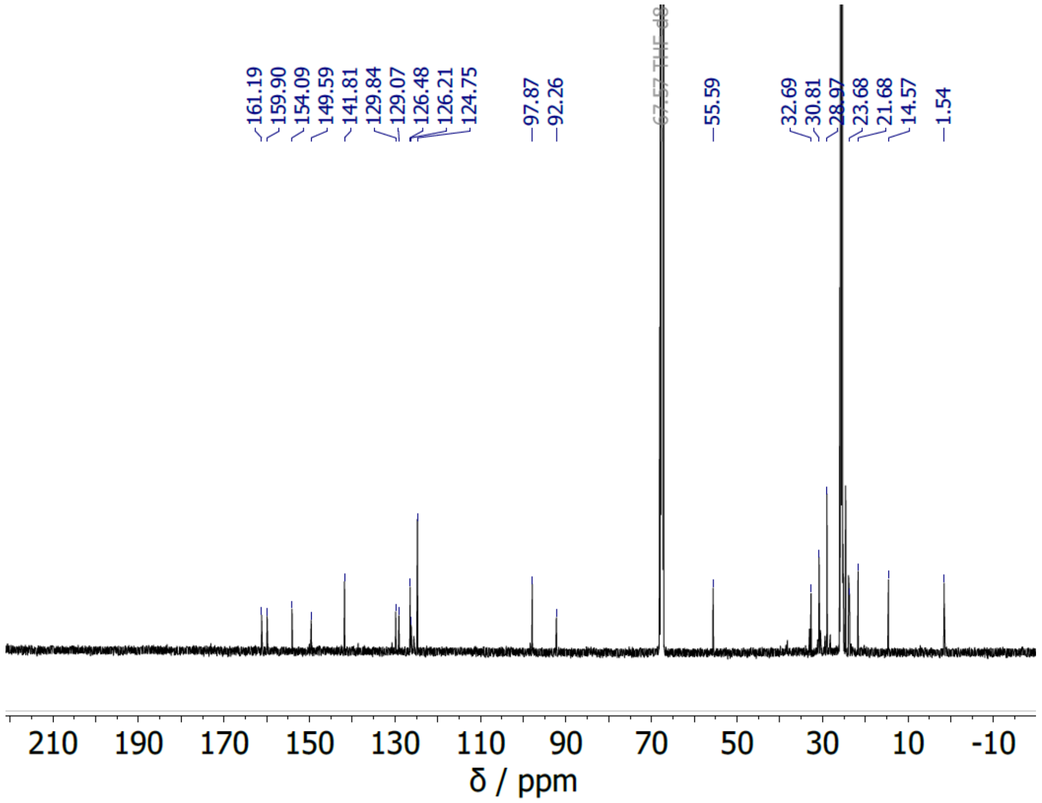

Figure S33. $^{13}C\{^1H\}$ spectrum (500 MHz) of **5** in THF-d$_8$.

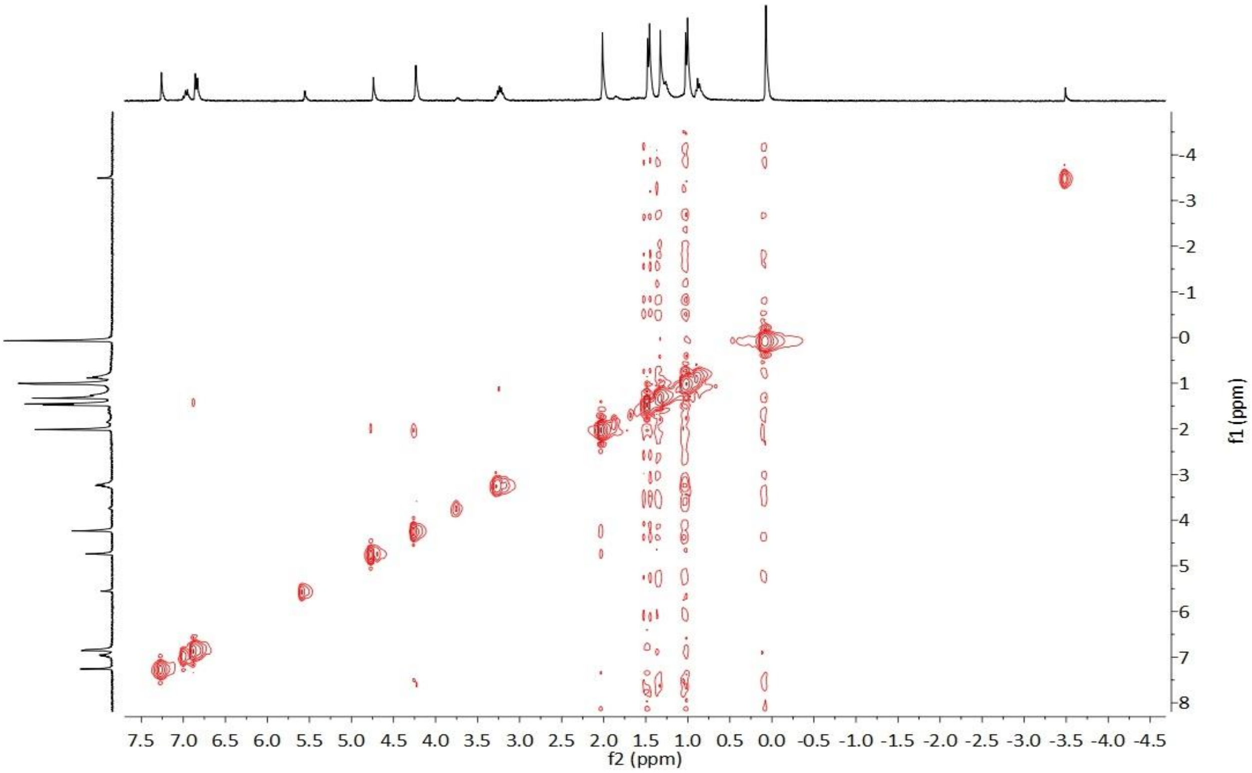

Figure S34. ^1H-^1H NOESY (300 MHz) spectrum of **5** in $CDCl_3$.

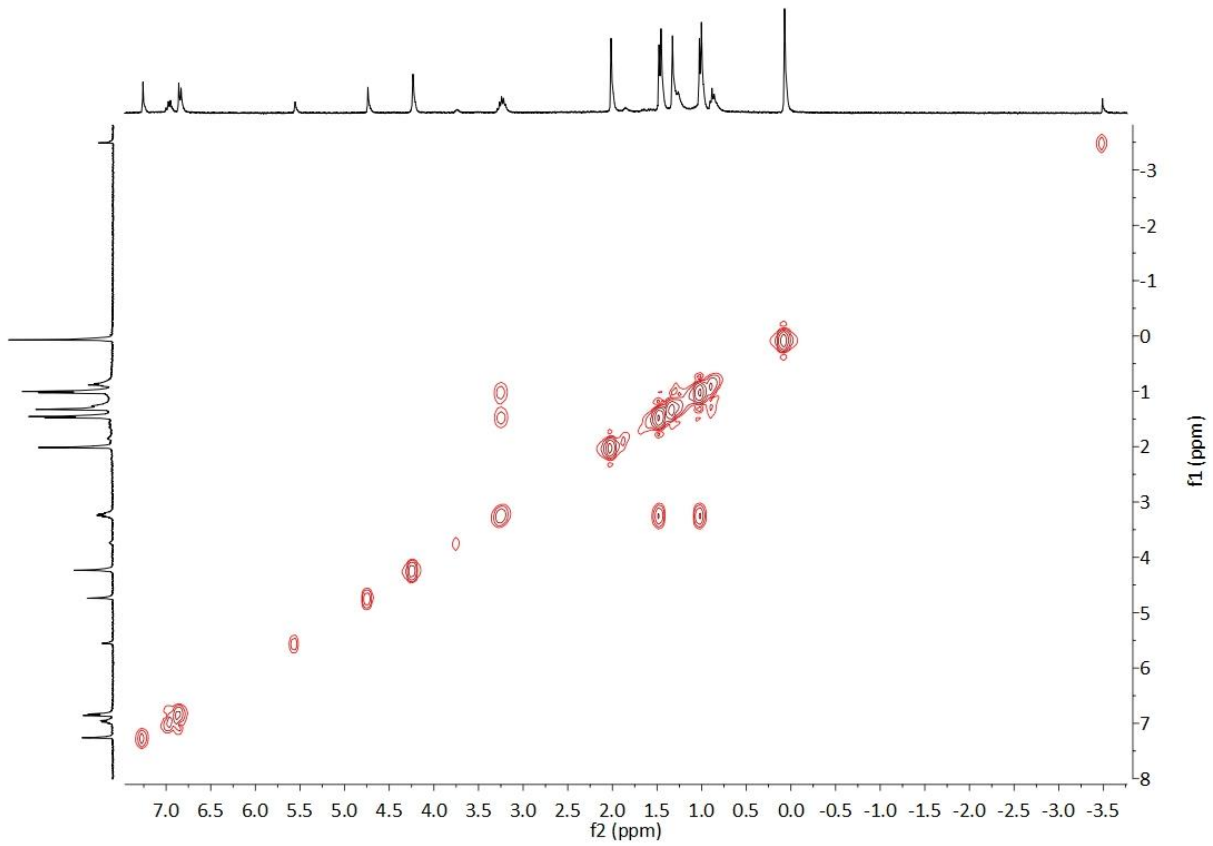

Figure S35: ^{1}H-^{1}H COSY (300 MHz) spectrum of **5** in $CDCl_3$.

D.2 IR spectra

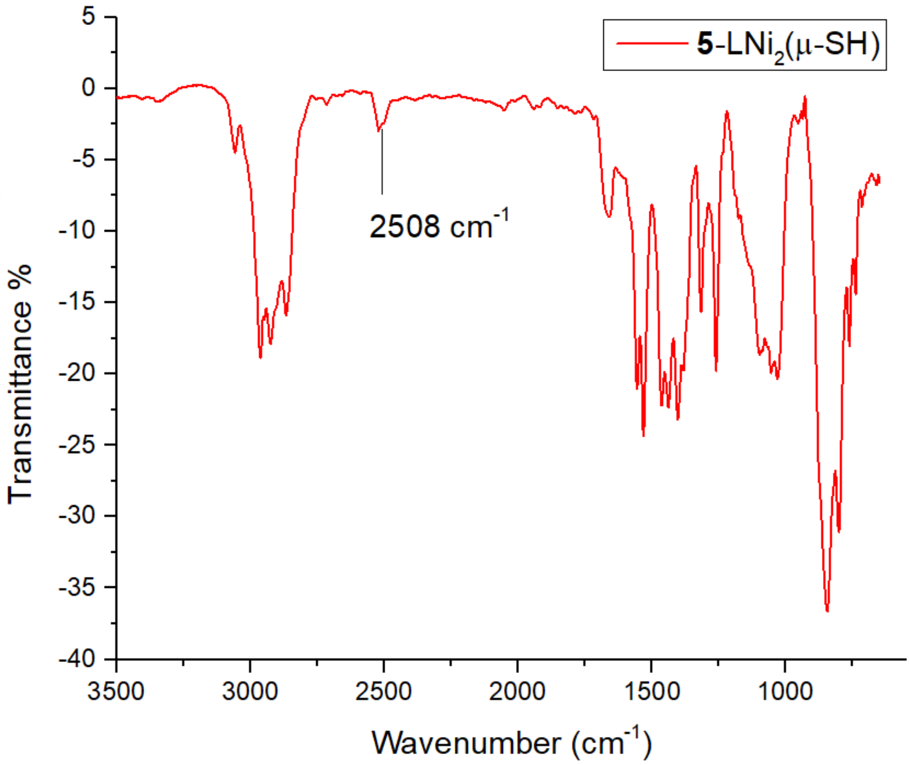

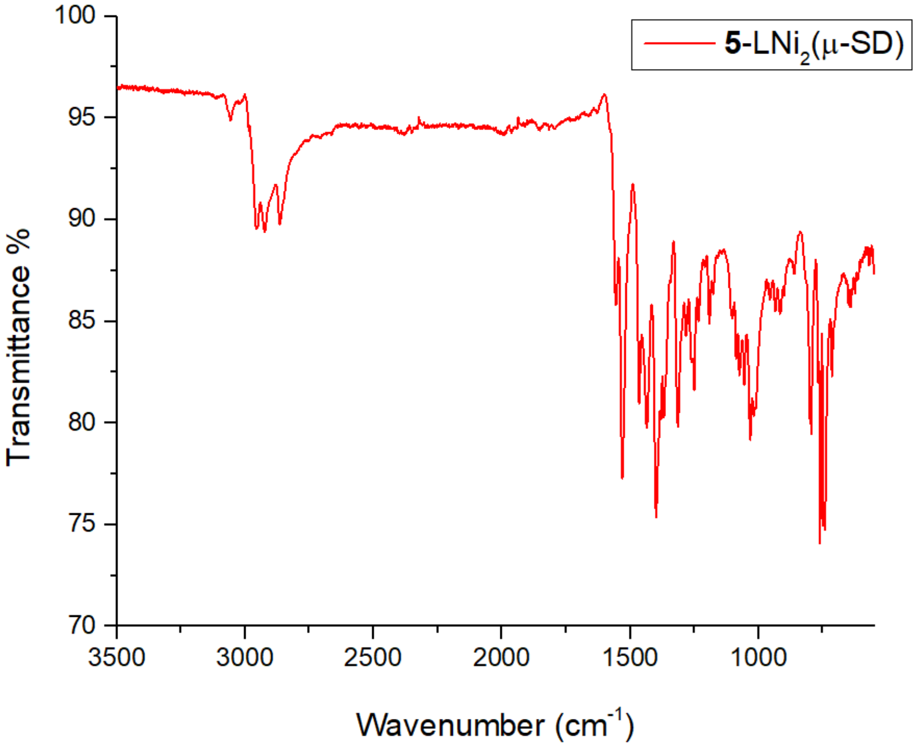

Figure S36: ATR IR spectra of crystalline material of **5** (top) and **5**^{D} (bottom).

D.3 UV-vis spectrum

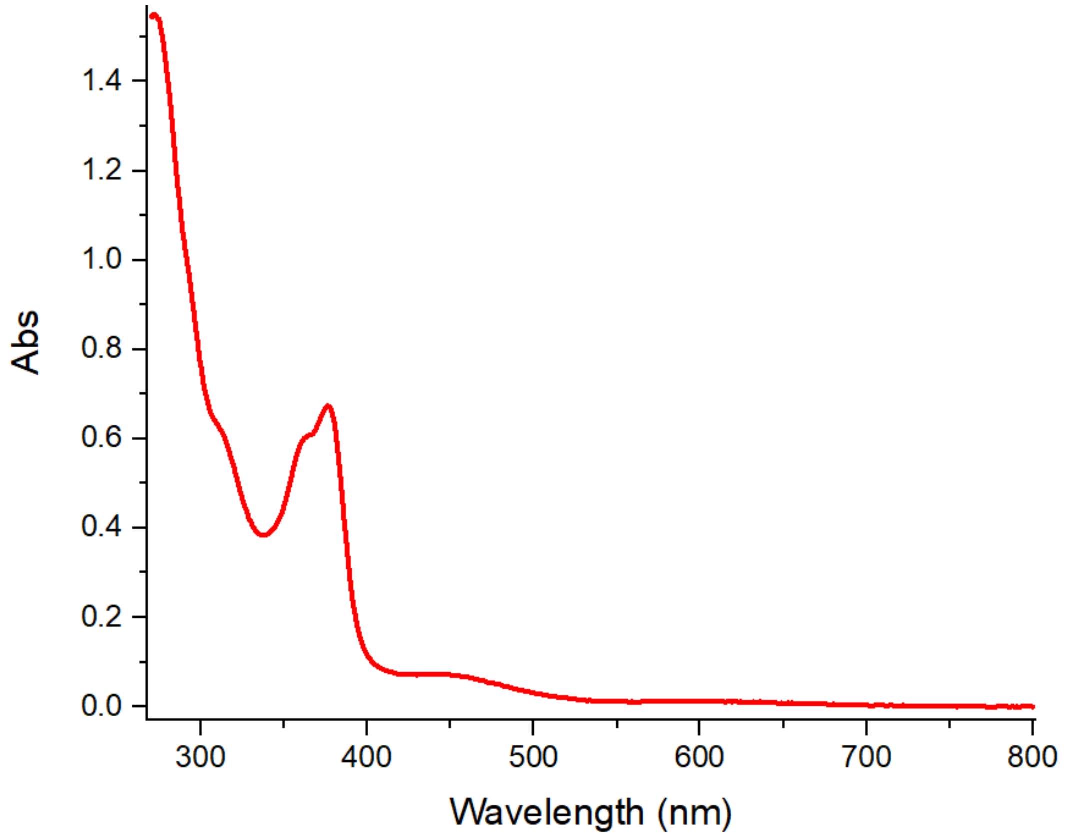

Figure S37. UV-vis spectrum of **5** in THF at room temperature.

D.4 CV measurement

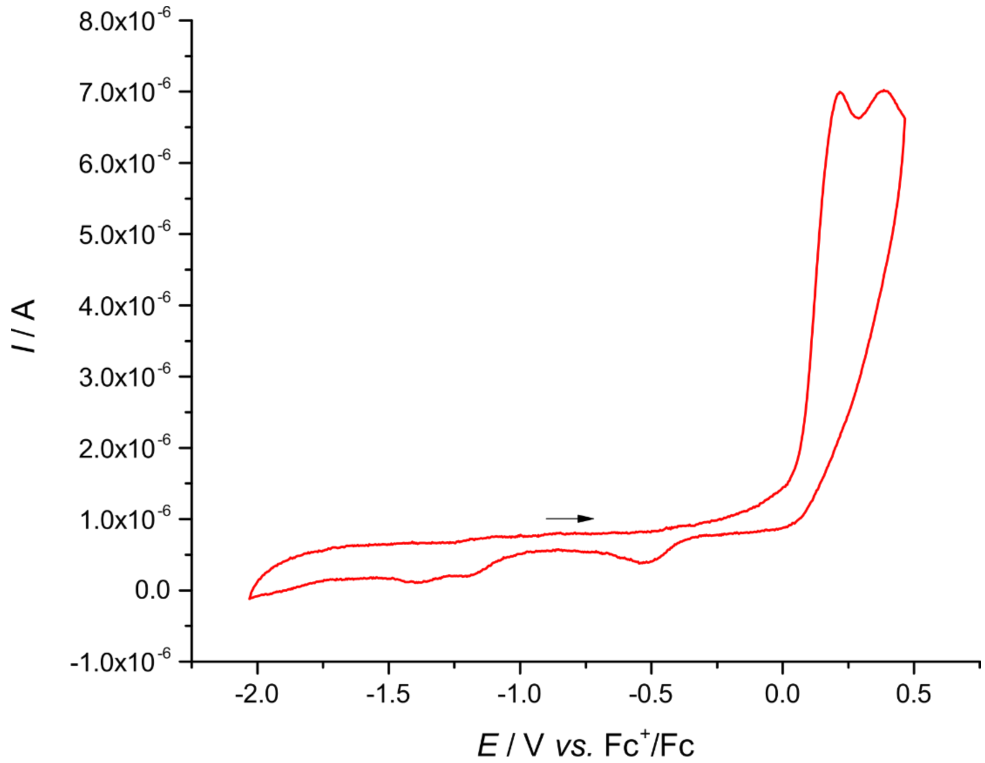

Figure S38. Cyclic voltammetry of **5** in THF at rt, 0.1 M NBu_4PF_6 supporting electrolyte; CE: Pt wire, RE: Ag wire, WE: 5 mm glassy carbon electrode.

E Complex 6

Oxidation of 3^M (M = Na, K) with $[Cp^*_2Fe]BF_4$, as descried in the main manuscript, is the most efficient way of obtaining pure **6**. Alternatively, **6** can also be obtained via direct oxidation of 3^M with O_2 at –30°C or via HAT from complex **5**.

E.1 CV measurements

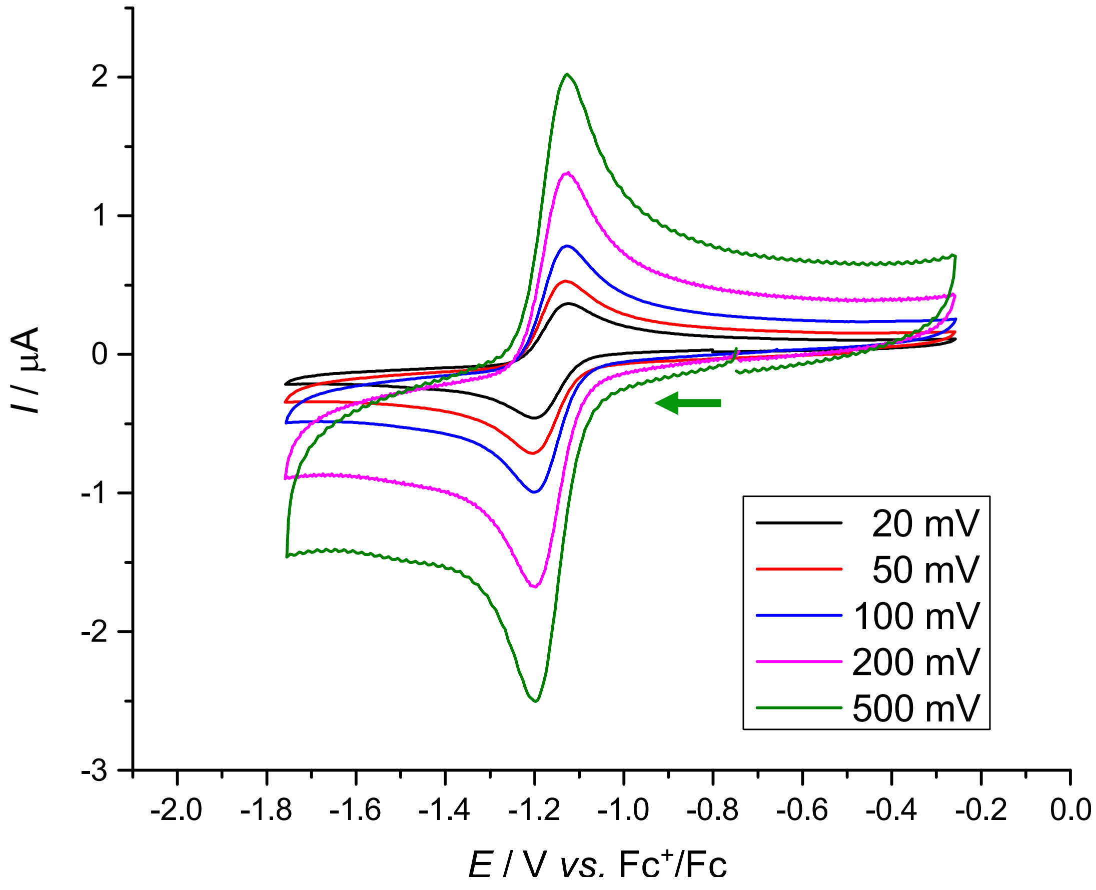

Figure S39. Cyclic voltammogram of **6** in THF (supporting electrolyte NBu_4PF_6; c = 0.2 M) at 25°C.

E.2 Substrate reactivity

HAT reactivity of complex **6** towards TEMPO-H in THF was monitored via UV-Vis spectroscopy at 293, 283, 273 and 263, and 253 K. Reactivity towards xanthene was found to be slow and was thus monitored only at 293 K.

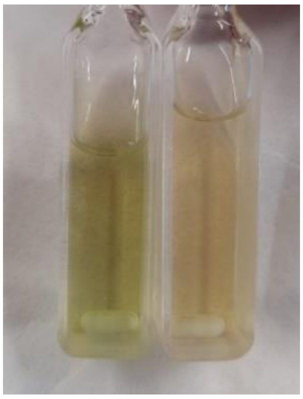

To a cuvette filled with 3 mL of a solution of **6** (0.2 mM) in THF and sealed with a septum, 20 µL of a solution of TEMPO-H / xanthene / DHA, respectively, in THF were added via a Hamilton syringe. A spectrum was recorded before addition of the substrate; afterwards UV-vis spectra were recorded every minute.

The reaction was followed with a great excess of H atom donor, (around 75, 150, 300 and 450 equivalents for TEMPO-H) in order to work in a pseudo-first order regime. Each kinetic measurement was performed three times.

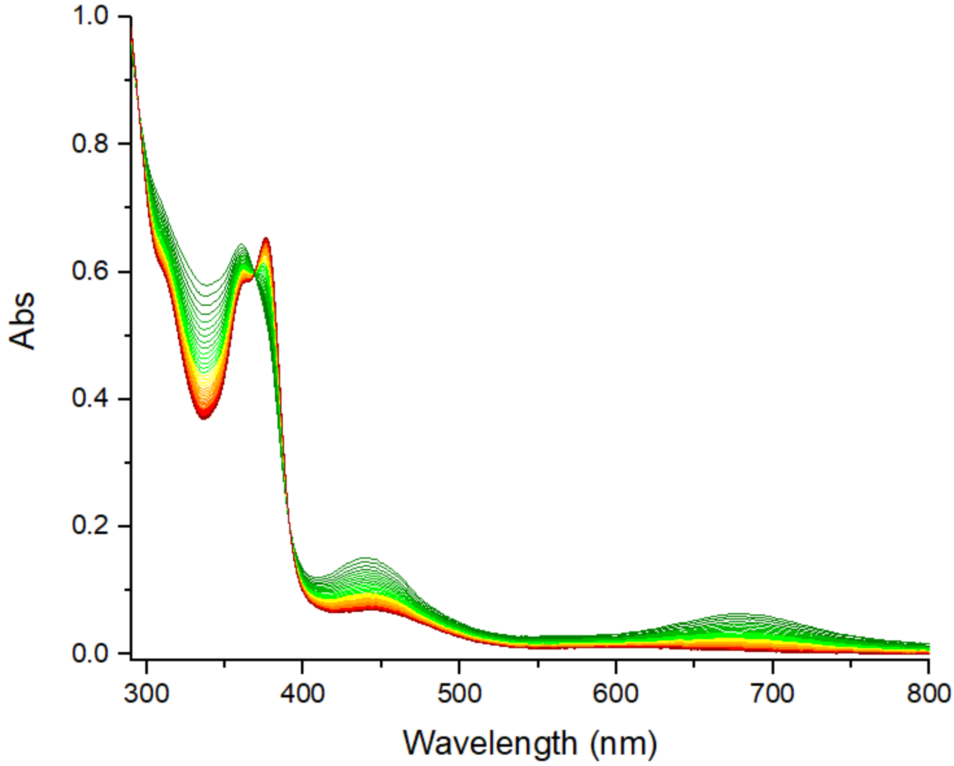

Figure S40. UV-vis spectrum of complex **6** (green) and its evolution upon addition of TEMPO-H (300 equivalents) at 283 K in THF (interval 60s). The characteristic band at 680 nm vanishes, those at 435 nm and 362 nm decrease with a concomitant rise of the band at 376 nm characteristic for complex **5** (red), and with isosbestic points at 368 and 392 nm.

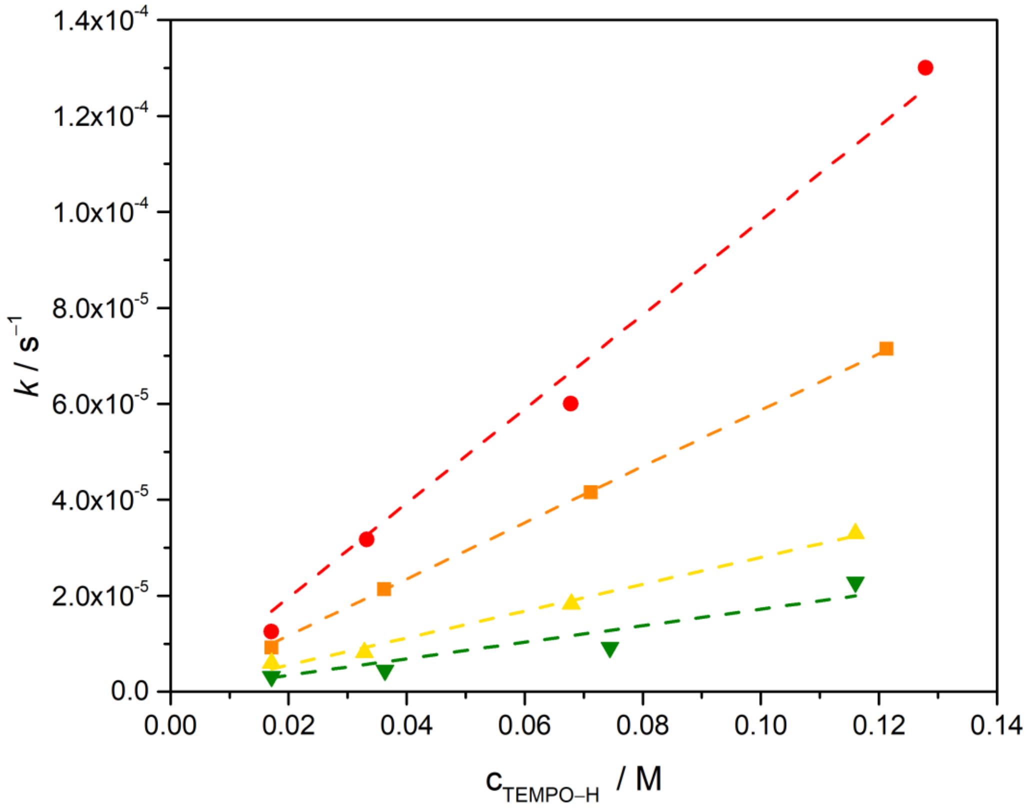

Figure S41 : Kinetic data for the reaction of complex **6** with TEMPO-H: plots of pseudo-first order rate constants k_{obs} (s^{-1}) versus concentration of TEMPO-H at 263, 273, 283 and 293 K in THF. The concentration of TEMPO-H corresponds to around 75, 150, 300 and 450 equiv. with respect to the substrate. Each measurement was repeated three times and the average value was used.

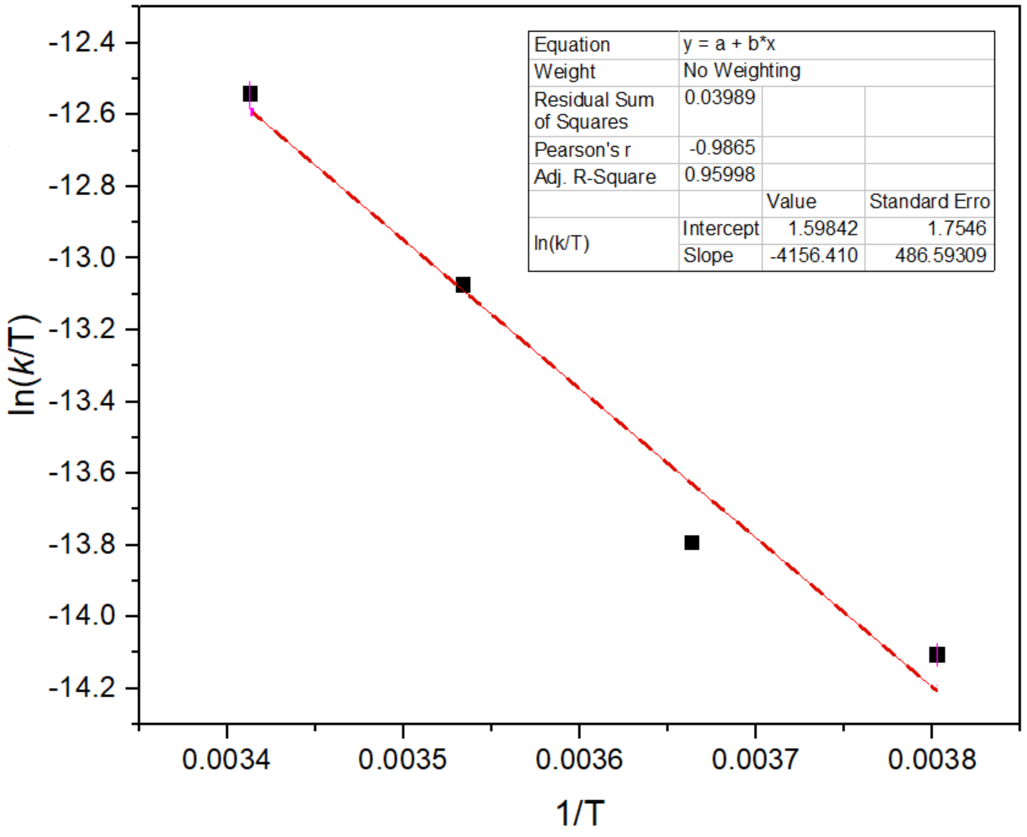

Figure S42 : Eyring plot obtained from the second order rate constants *k*(T) (M^{-1} s^{-1}) measured at 263, 273, 283 and 293 K. From a linear fit the activation parameters $\Delta H^{\ddagger}$ = (34.5 ± 4.1) kJ mol^{-1} = (8.2 ± 1.0) kcal mol^{-1} and $\Delta S^{\ddagger}$ = –(184 ± 13) J mol^{-1} K^{-1} = –(44 ± 3) cal mol^{-1} K^{-1} are obtained, which translate into an activation free energy $\Delta G^{\ddagger}$(293 K) = (88.3 ± 7.9) kJ mol^{-1}= (21.1 ± 1.9) kcal mol^{-1}.

Table S1: Substrates investigated and their BDFE values[5,6] in THF together with second order rate constants *k* for the reaction of complex **6** with TEMPO-H and xanthene at 293 K.

**Substrate**	**BDFE (kcal/mol) in THF**	**HAT reactivity**	***k*** **(s^{-1}·M^{-1}) at 293 K**
9,10-dihydroanthracene (DHA)	76.3	no	-
xanthene	72.2	Yes	$2.30 \cdot 10^{-4}$
TEMPO-H	65.5	yes	$1.16 \cdot 10^{-3}$

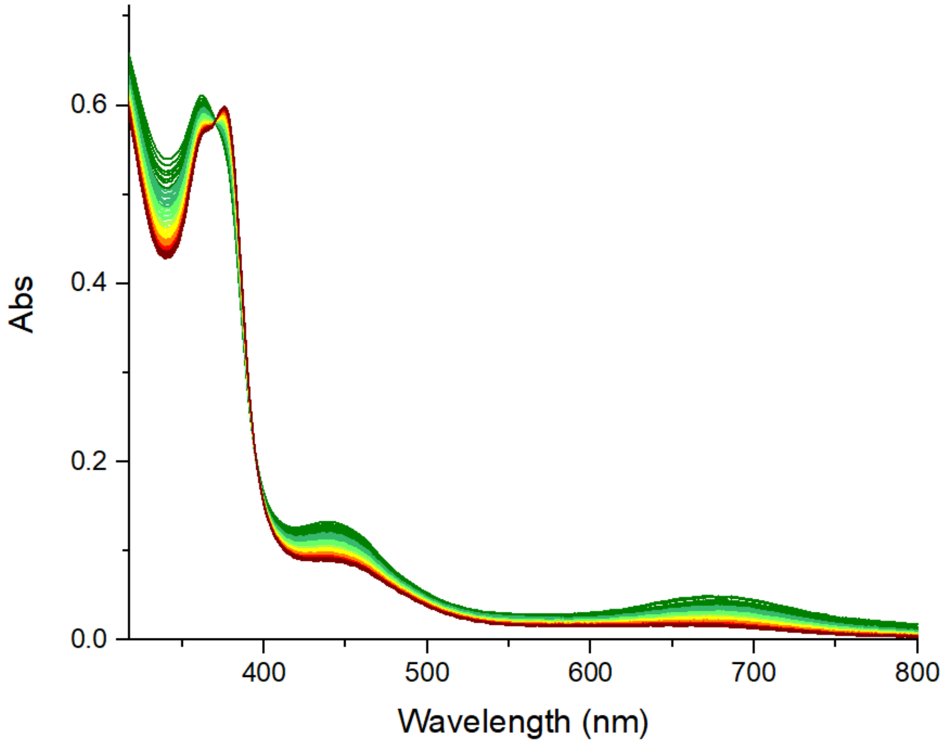

Figure S43. UV-vis spectrum of complex **6** (green) and its evolution upon addition of xanthene (300 equivalents) at 293 K in THF (interval 60s). The characteristic band at 680 nm vanishes, those at 435 nm and 362 nm decrease with a concomitant rise of the band at 376 nm characteristic for complex **5** (red), and with isosbestic points at 368 and 392 nm.

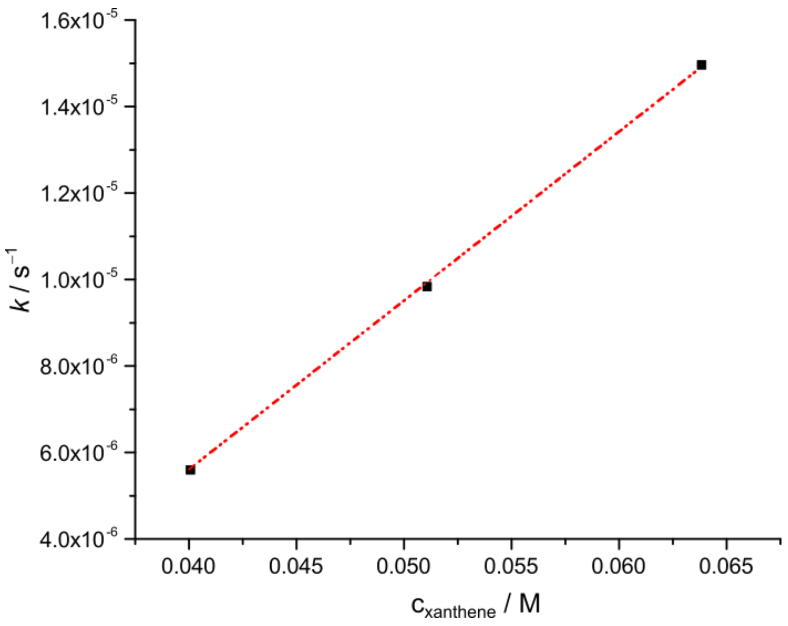

Figure S44. k_{obs} values for the reaction of **6** with xanthene, measured at 293 K in THF and plotted versus the concentration of xanthene. The concentration of xanthene corresponds to 450, 300 and 150 equiv. with respect to **6**. Second order rate constant $k = 2.30 \cdot 10^{-4}$ $M^{-1}s^{-1}$ at 293 K.

E.3 Protonation of radical complex 6

To test the possibility of protonating **6**, and to get information about the pK_a of putative **6H$^+$**, [HDBU]PF_6 was added to complex **6** in THF and the reaction monitored by UV-vis spectroscopy.

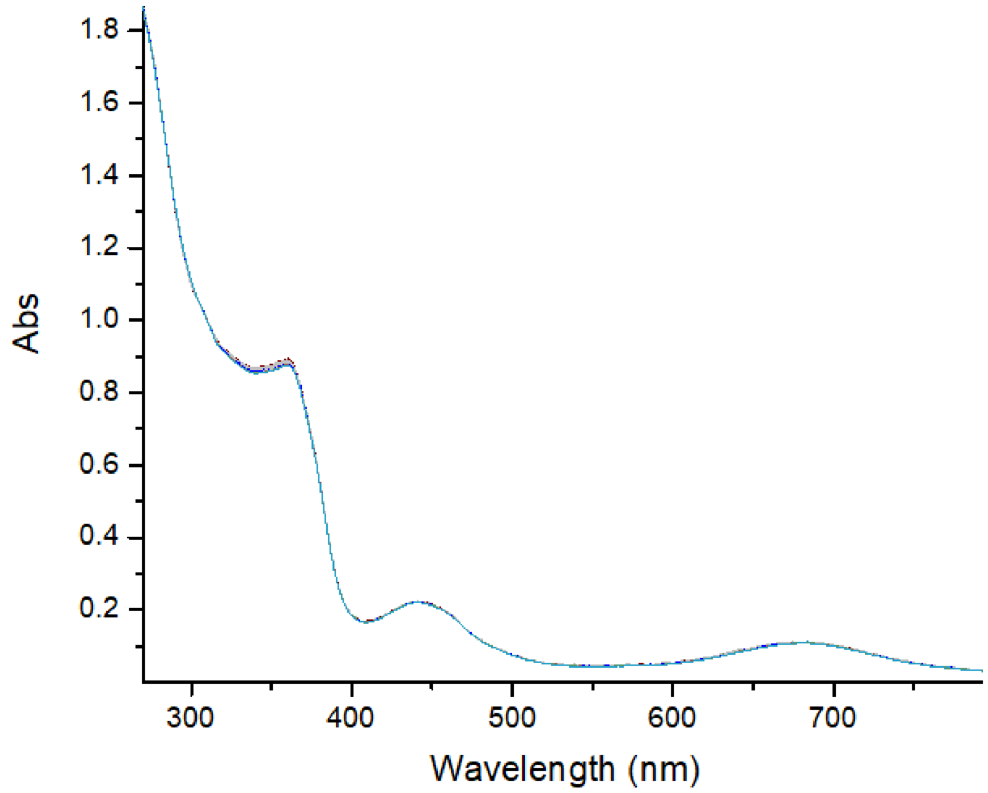

Figure S45. UV-vis monitoring of the titration of a 10^{-4} M solution of **6** in THF with [HDBU]PF_6 (pK_a = 19.1 in THF).[11] The reaction mixture was allowed to equilibrate after each addition. No changes in the spectrum were observed also in presence of excess of acid. Spectra were recorded over a period of two hours.

E.4 Comparison of concerted and stepwise pathways

In this section the thermodynamic parameters concerning the possibility of stepwise ET/PT or PT/ET pathways instead of a concerted PCET reaction (CPET) are evaluated.

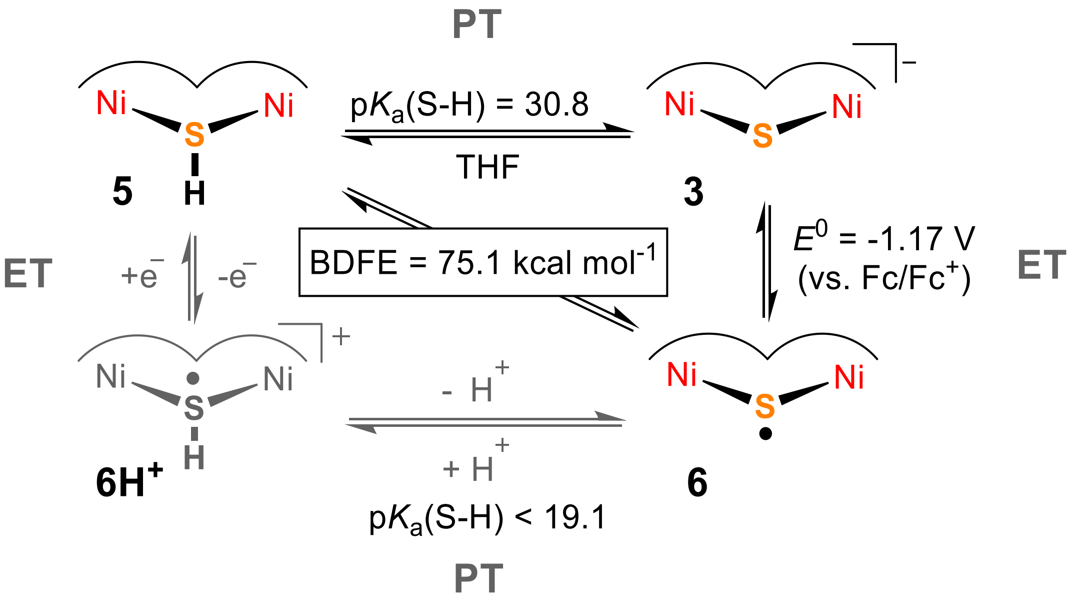

Figure S46. Thermodynamic PCET square scheme for the interconversion of **6** and **5**, including stepwise pathways via **3** and **6H⁺**.

$\Delta G°_{CPET}$ for the reaction of sulfur radical complex **6** with TEMPO-H to give **5** is calculated using the following equation, with a value of 65.5 kcal mol^{-1} for BDFE(TEMPO-H) in THF.[6]

$$\Delta G°_{CPET} = \text{BDFE(TEMPO-H)} - \text{BDFE(S-H)} = -9.6 \text{ kcal mol}^{-1} \ (-40.2 \text{ kJ mol}^{-1})$$

For the sulfido complexes **3** has been determined in this work $E°_{1/2} = -1.17$ V versus Fc^+/Fc (assuming $E_{½}$ as a good approximation of $E°$), and the pK_a of putative **6H⁺** has been shown to be lower than that of [HDBU]⁺ (pK_a= 19.1, Figure S42).[12] For TEMPO-H the following values can be found in literature:

$E°$(TEMPO-H$^{·+}$/TEMPO-H) = +0.58 V and pK_a (TEMPO-H) = 48 in THF.[6]

Thus, we can derive the free energy for the ET reaction of **6** with TEMPO-H to give **3** and TEMPO-H$^{·+}$:

$$\Delta G°_{ET} = -F\Delta E° = -(23.06 \text{ kcal mol}^{-1} \text{V}^{-1})\cdot\Delta E° = -(98.48 \text{ kJ mol}^{-1}\text{V}^{-1})\cdot\Delta E°$$

$$\Delta G°_{ET} = -(23.06 \text{ kcal mol}^{-1} \text{V}^{-1})\cdot 1.75 \text{ V} = 40.4 \text{ kcal mol}^{-1} \ (169.0 \text{ kJ mol}^{-1})$$

Similarly, we can derive the free energy for the PT reaction of **6** with TEMPO-H to give **6H⁺** and TEMPO⁻:

$\Delta G°_{PT} = –RT \ln(K) = –2.303\ RT(\Delta pK_a)= –(1.37\ \text{kcal mol}^{–1})\cdot\Delta pK_a$ (at 298 K) $= –(5.73\ \text{kJ mol}^{–1})\cdot \Delta pK_a$

$\Delta G°_{PT} > 39.6$ kcal mol$^{–1}$ (165.6 kJ mol$^{–1}$) with $\Delta pK_a > 28.9$

Since these values are much larger than the experimentally observed activation free energy of $\Delta G^{‡}_{293}$ = (21.1 ± 1.9) kcal mol$^{–1}$ (88.3 ± 7.9) kJ mol$^{–1}$ for the reaction of **6** with TEMPO-H to give **5**, the stepwise pathways can be safely excluded and the reaction should follow a concerted pathway (CPET).

E.5 Mass spectrometry

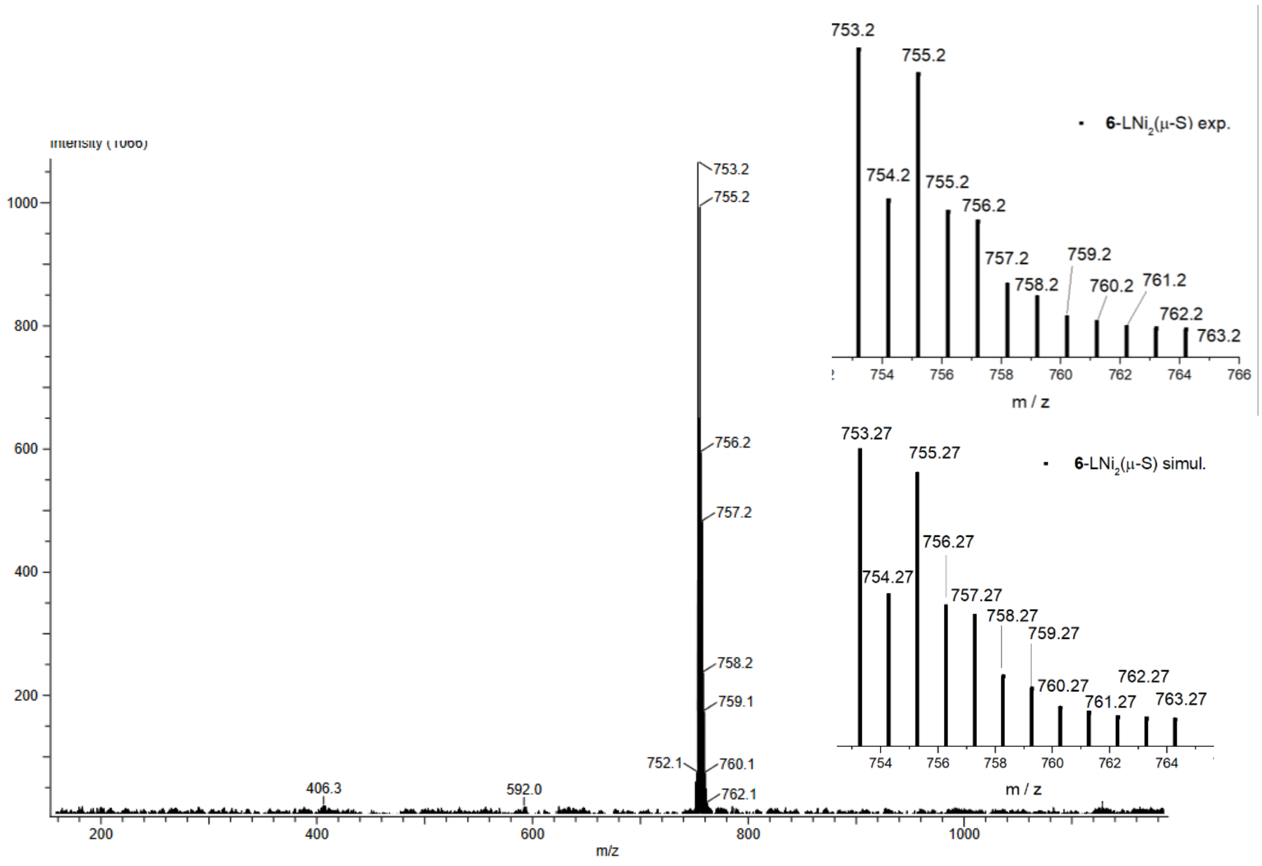

Figure S47: LIFDI spectrum of a 10^{-4} M solution of **6** in toluene. The inset shows the experimental (top) and simulated (bottom isotopic distribution pattern for [LNi_2S] at *m/z* = 753.2.

E.6 Reaction of 5 with phenoxy radical TTBP to give 6

To a pre-cooled and brown solution of **5** (15.6 mg, 0.02 mmol, 1 equiv) in THF (1 mL) was added 2,4,6-tri(*tert*-butyl)phenoxy radical (TTBP; 3.2 mg, 0.04 mmol, 2 equiv). The reaction mixture was allowed to react for 12 hours at −30°C. After layering hexane on the reaction mixture and keeping it at −30°C for two days, green single crystals of **6** suitable for X-ray diffraction were obtained.

E.7 IR spectrum

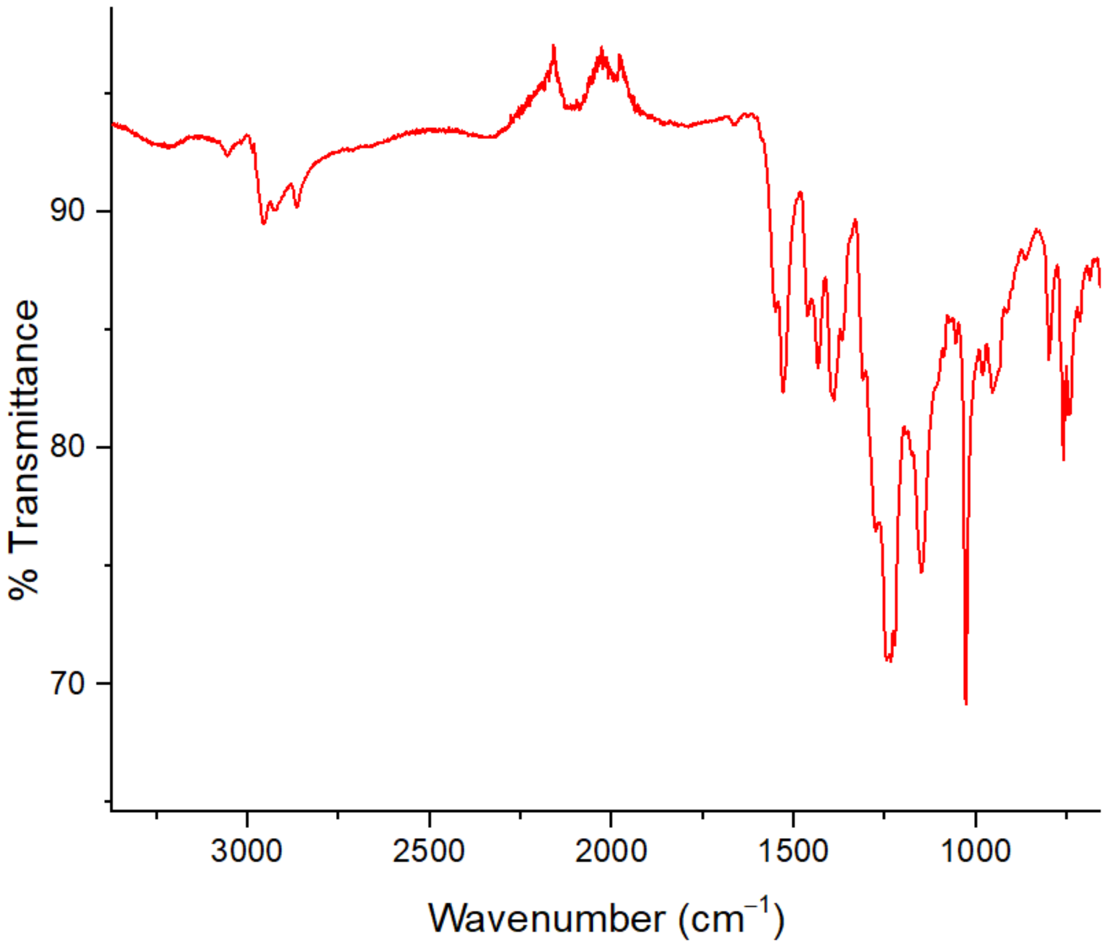

Figure S48. ATR IR spectrum of crystalline material of complex **6**.

F X-ray Crystallography

Single-Crystal X-Ray structure determinations. Crystal data and details of the data collections are given in Tables S2 and S3, selected bond lengths angles in Table 1, molecular structures are shown in Figures S49 – S54. X-ray data were collected on a STOE IPDS II or a BRUKER D8-QUEST diffractometer (monochromated Mo-Kα radiation, λ = 0.71073 Å) by use of ω or ω and ϕ scans at low temperature. The structures were solved with SHELXT and refined on F^2 using all reflections with SHELXL.[7] Non-hydrogen atoms were refined anisotropically. Most hydrogen atoms were placed in calculated positions and assigned to an isotropic displacement parameter of 1.5/1.2 U_{eq}(C). The sulfur bound hydrogen atom in **5** was refined freely. One potassium bound thf and one lattice thf molecule were found to be disordered about two positions in **2**$^{\mathbf{K}}$ (occupancy factors: 0.656(19) / 0.344(19) (K-bound); 0.569(16) / 0.431(16) (lattice)) and were refined using DFIX (*d*(C–C) = 1.54 Å, *d*(C–O) = 1.44), SAME, and RIGU restraints and EADP constraints.In case of **3**$^{\mathbf{K}}$ all potassium bound thf molecules were found to be disordered about two positions (occupancy factors: 0.507(7) / 0.493(7); 0.638(9) / 0.362(9); 0.516(7) / 0.484(7)). DFIX (*d*(C–C) = 1.54 Å, *d*(C–O) = 1.44), SADI (*d*(C···O)), SAME, and RIGU restraints and EADP constraints were used to model the disordered parts. All sodium bound thf molecules in both crystallographically independent molecules were found to be disordered about two positions in **3**$^{\mathbf{Na}}$ (occupancy factors: 0.79(2) / 0.21(2); 0.227(14) / 0.773(14); 0.840(10) / 0.160(10); 0.748(9) / 0.252(9)) and were refined using DFIX (*d*(C–C) = 1.54 Å, *d*(C–O) = 1.44), SADI (*d*(C···O), *d*(Na–O)), SAME, and RIGU restraints and EADP constraints. **3**$^{\mathbf{Na}}$ was further refined as inversion twin (BASF = 0.41(2)). In case of **5** and **6** one uncoordinated thf molecule was found to be disordered about a center of inversion with an additional positional disorder of the oxygen atom. The latter atom was refined at ¼ occupancy and the carbon atoms at ½ occupancy. DFIX restraints (*d*(C–C) = 1.54 Å, *d*(C–O) = 1.44) were applied to model the disorder. In **4** one isopropyl moiety was found to be disordered about two positions (occupancy factors: 0.523(9) / 0.477(9)) and was refined using SAME, SADI and RIGU restraints. Furthermore, disordered thf and hexane molecules were refined using DFIX (thf: *d*(C–C) = 1.54 Å, *d*(C–O) = 1.44; C_6H_{14}: *d*(CH_2–CH_3) = 1.51 Å, *d*(CH_2–CH_2) = 1.52 Å), SADI (*d*(C···O)) and RIGU restraints. After initial refinement the occupancy factors were fixed (thf: 0.6 / C_6H_{14}: 0.4). Face-indexed absorption corrections were performed numerically with the program X-RED[8] or by the multi-scan method with SADABS.[9]

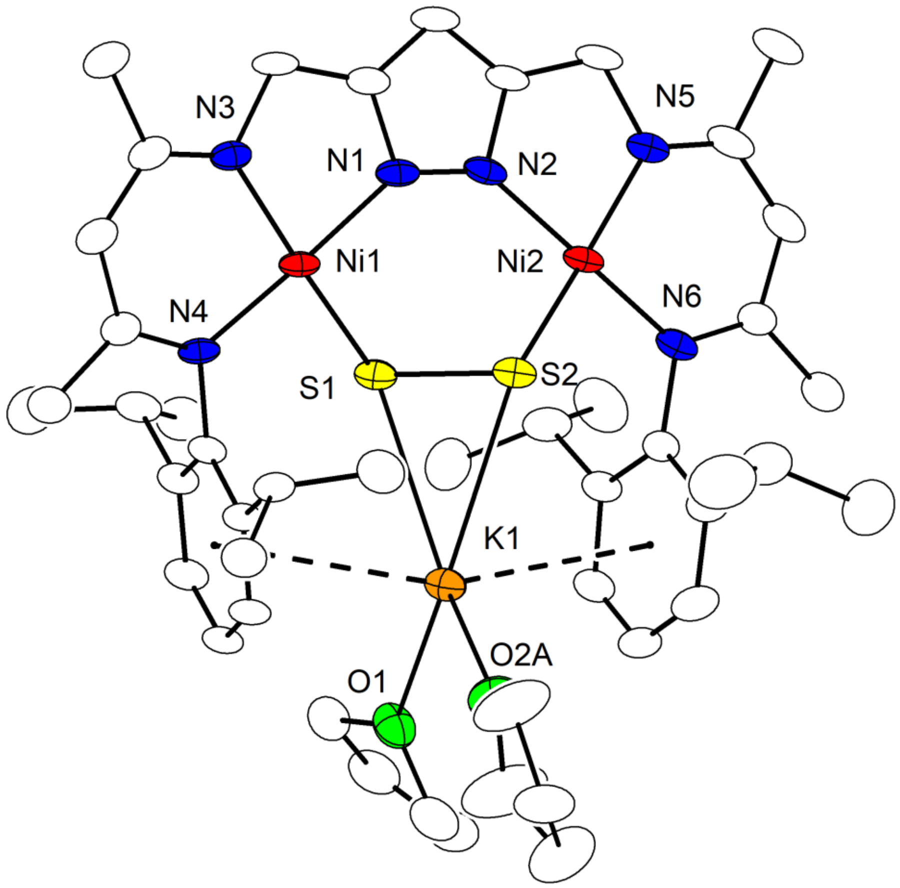

Figure S49. Plot (30% probability thermal ellipsoids) of the molecular structure of **2**^{K} (disorder and hydrogen atoms omitted for clarity). Cg1 is defined by the ring atoms close to N4. Cg2 is defined by the ring atoms close to N6. Selected bond lengths [Å] and angles [°]: Ni1–N1 1.928(6), Ni1–N3 1.915(5), Ni1–N4 1.925(5), Ni2–N2 1.931(6), Ni2–N5 1.904(5), Ni2–N6 1.914(6), Ni1–S1 2.1672(19), Ni2–S2 2.1599(19), Ni1···Ni2 4.2902(13), K1–S1 3.103(2), K1–S2 3.157(2), K1–Cg1 3.3570(18), K1–Cg2 3.2585(17), S1–S2 2.1599(19); N3–Ni1–N4 92.4(2), N3–Ni1–N1 84.9(2), N4–Ni1–N1 177.1(2), N3–Ni1–S1 170.03(16), N4–Ni1–S1 92.41(15), N1–Ni1–S1 90.41(16), N5–Ni2–N6 93.8(2), N5–Ni2–N2 84.4(2), N6–Ni2–N2 174.1(2), N5–Ni2–S2 167.80(16), N6–Ni2–S2 92.51(17), N2–Ni2–S2 90.38(16), S2–S1–Ni1 108.48(9), S1–S2–Ni2 106.89(10), Ni1–S1–S2–Ni2 81.1(1), Cg1–K1–Cg2 156.1(1).

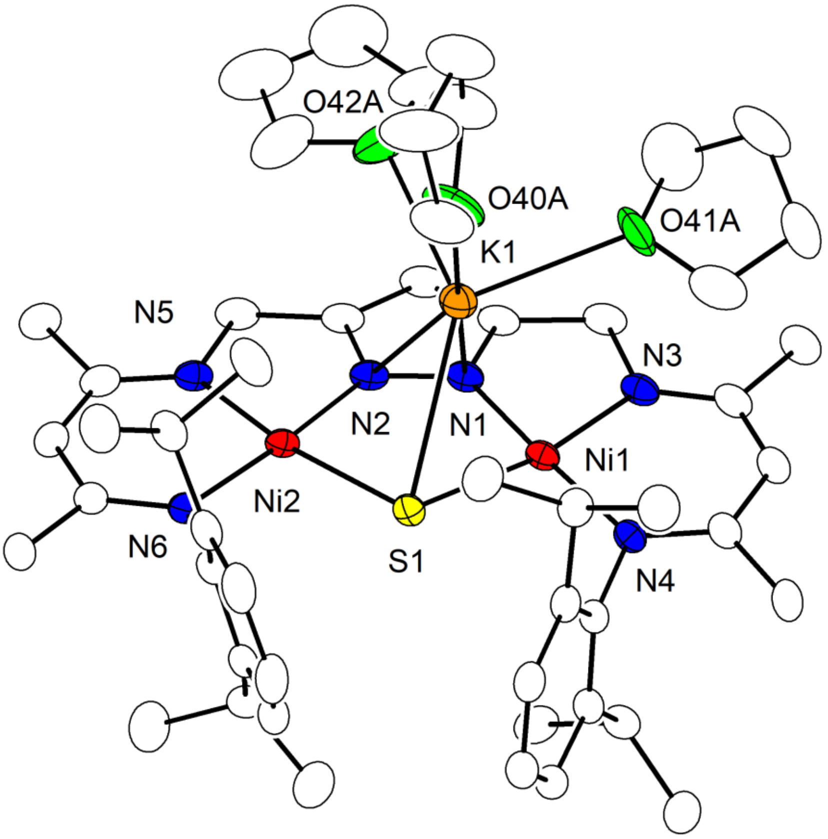

Figure S50. Plot (30% probability thermal ellipsoids) of the molecular structure of **3**^{K} (disorder hydrogen atoms omitted for clarity). Selected bond lengths [Å] and angles [°]: Ni1–N1 1.816(2), Ni1–N3 1.925(2), Ni1–N4 1.904(2), Ni2–N2 1.820(2), Ni2–N5 1.932(2), Ni2–N6 1.914(2), Ni1–S1 2.2404(7), Ni2–S1 2.2435(7), Ni1···K1 3.1975(7), Ni2···K1 3.7910(7), Ni1···Ni2 3.6515(5), K1–N1 3.160(2), K1–N2 3.446(2), K1–S1 3.1968(9); N1–Ni1–N4 176.47(9), N1–Ni1–N3 82.01(10), N4–Ni1–N3 94.51(9), N1–Ni1–S1 86.10(7), N4–Ni1–S1 97.40(6), N3–Ni1–S1 168.02(7), N2–Ni2–N6 176.17(9), N2–Ni2–N5 82.12(9), N6–Ni2–N5 94.41(9), N2–Ni2–S1 85.81(7), N6–Ni2–S1 97.73(6), N5–Ni2–S1 167.60(7), Ni1–S1–Ni2 109.05(3).

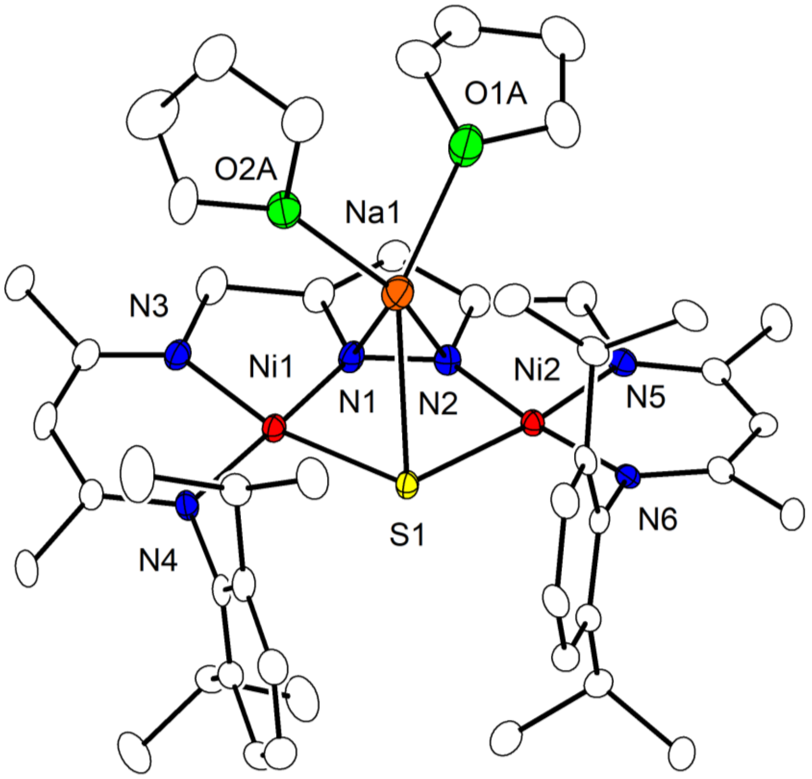

Figure S51. Plot (30% probability thermal ellipsoids) of the molecular structure of **3**$^{\mathbf{Na}}$ (disorder and hydrogen atoms omitted for clarity). Only one of the two crystallographically independent molecules is shown. Selected bond lengths [Å] and angles [°]: Ni1–N1 1.812(6), Ni1–N3 1.918(6), Ni1–N4 1.896(6), Ni2–N2 1.823(6), Ni2–N5 1.927(6), Ni2–N6 1.906(6), Ni1–S1 2.2489(19), Ni2–S1 2.2360(19), Ni1···Na1 2.977(3), Ni2···Na1 3.080(3), Ni1···Ni2 3.6262(13), Na1–N1 2.796(8), Na1–N2 2.922(7), Na1–S1 2.738(4); N1–Ni1–N4 175.1(3), N1–Ni1–N3 81.6(3), N4–Ni1–N3 95.3(3), N1–Ni1–S1 87.1(2), N4–Ni1–S1 96.34(19), N3–Ni1–S1 167.1(2), N2–Ni2–N6 174.2(3), N2–Ni2–N5 81.6(2), N6–Ni2–N5 95.0(3), N2–Ni2–S1 87.3(2), N6–Ni2–S1 96.31(17), N5–Ni2–S1 168.54(19), Ni1–S1–Ni2 107.91(7).

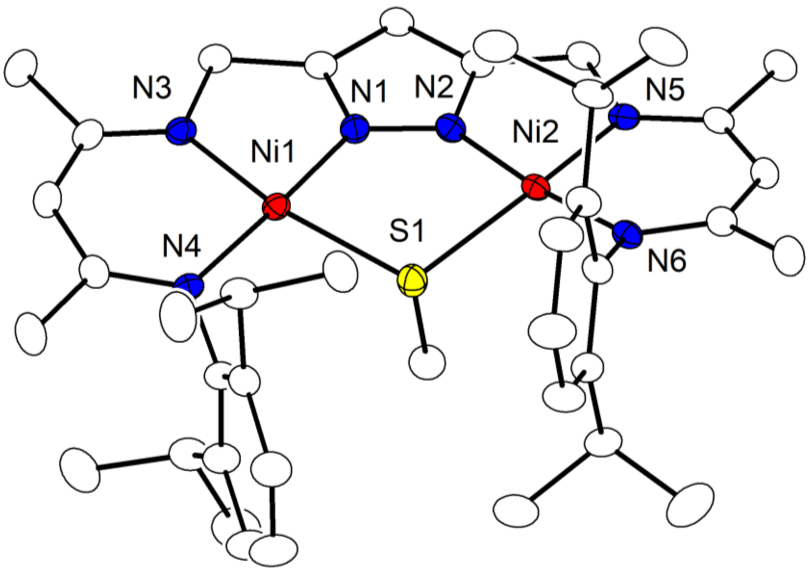

FIgure S52. Plot (30% probability thermal ellipsoids) of the molecular structure of **4** (hydrogen atoms omitted for clarity). Selected bond lengths [Å] and angles [°]: Ni1–N1 1.821(2), Ni1–N3 1.899(2), Ni1–N4 1.899(2), Ni2–N2 1.818(2), Ni2–N6 1.890(2), Ni2–N5 1.895(2), Ni1–S1 2.2810(8), Ni2–S1 2.2635(8), Ni1···Ni2 3.6488(4); N1–Ni1–N4 176.16(9), N1–Ni1–N3 81.84(9), N4–Ni1–N3 94.95(9), N1–Ni1–S1 86.00(7), N4–Ni1–S1 97.13(7), N3–Ni1–S1 167.70(7), N2–Ni2–N6 173.28(9), N2–Ni2–N5 81.88(10), N6–Ni2–N5 95.59(9), N2–Ni2–S1 86.11(7), N6–Ni2–S1 97.61(7), N5–Ni2–S1 162.93(7), Ni1–S1–Ni2 106.81(3).

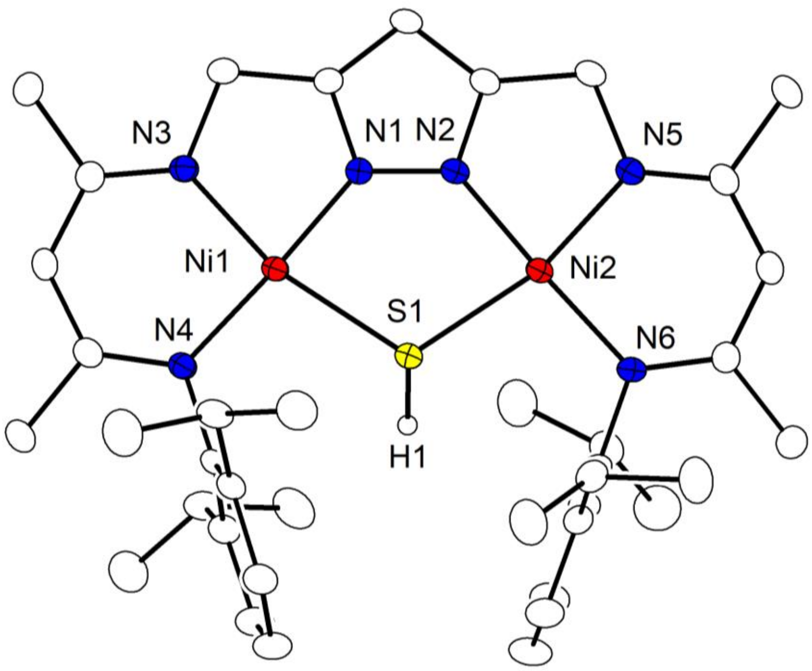

Figure S53. Plot (30% probability thermal ellipsoids) of the molecular structure of **5** (most hydrogen atoms omitted for clarity). Selected bond lengths [Å] and angles [°]: Ni1–N1 1.8293(17), Ni1–N3 1.9031(19), Ni1–N4 1.9006(17), Ni2–N2 1.8272(19), Ni2–N5 1.8998(18), Ni2–N6 1.8948(19), Ni1–S1 2.2665(7), Ni2–S1 2.2765(6), Ni1···Ni2 3.7126(5), S1–H1 1.21(3) ; N1–Ni1–N4 176.80(8), N1–Ni1–N3 82.11(8), N4–Ni1–N3 94.83(8), N1–Ni1–S1 84.32(6), N4–Ni1–S1 98.82(6), N3–Ni1–S1 165.18(6), N2–Ni2–N6 173.73(9), N2–Ni2–N5 82.48(8), N6–Ni2–N5 94.33(8), N2–Ni2–S1 84.15(6), N6–Ni2–S1 99.81(6), N5–Ni2–S1 163.82(6), Ni1–S1–Ni2 109.62(2).

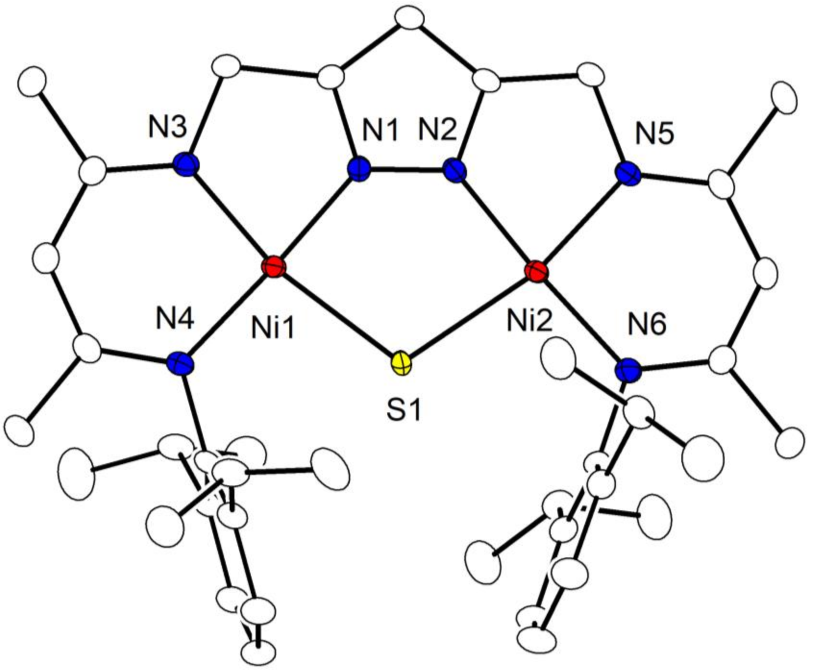

Figure S54. Plot (30% probability thermal ellipsoids) of the molecular structure of **6** (hydrogen atoms omitted for clarity). Selected bond lengths [Å] and angles [°]: Ni1–N1 1.825(2), Ni1–N3 1.896(2), Ni1–N4 1.898(2), Ni2–N2 1.827(2), Ni2–N5 1.893(2), Ni2–N6 1.893(2), Ni1–S1 2.2778(7), Ni2–S1 2.2889(7), Ni1···Ni2 3.7047(5); N1–Ni1–N3 82.03(9), N1–Ni1–N4 177.01(9), N3–Ni1–N4 95.00(9), N1–Ni1–S1 85.33(7), N3–Ni1–S1 166.54(7), N4–Ni1–S1 97.66(7), N2–Ni2–N5 82.25(9), N2–Ni2–N6 174.11(10), N5–Ni2–N6 94.47(9), N2–Ni2–S1 85.15(7), N5–Ni2–S1 165.18(7), N6–Ni2–S1 98.72(7), Ni1–S1–Ni2 108.44(3).

Table S2. Crystal data and refinement details for **2**^{K}, **3**^{K}, and **3**^{Na}.

**compound**	**2**^{K}	**3**^{K}	**3**^{Na}
empirical formula	$C_{51}H_{77}KN_6Ni_2O_3S_2$	$C_{51}H_{77}KN_6Ni_2O_3S$	$C_{47}H_{69}N_6NaNi_2O_2S$
moiety formula	$C_{47}H_{69}KN_6Ni_2O_2S_2$, C_4H_8O	$C_{51}H_{77}KN_6Ni_2O_3S$	$C_{47}H_{69}N_6NaNi_2O_2S$
formula weight	1042.82	1010.76	922.55
T [K]	133(2)	133(2)	120(2)
crystal size [mm^3]	0.450 x 0.230 x 0.140	0.500 x 0.490 x 0.460	0.290 x 0.247 x 0.226
crystal system	monoclinic	triclinic	monoclinic
space group	$P2_1/n$ (No. 14)	P–1 (No. 2)	Pn (No. 7)
a [Å]	17.3254(11)	12.9841(6)	13.7796(7)
b [Å]	15.5369(6)	13.4180(6)	24.1138(12)
c [Å]	20.4719(12)	18.0609(8)	14.4782(8)
α [°]	90	75.444(4)	90
β [°]	109.707(4)	74.712(4)	101.193(2)
γ [°]	90	62.255(3)	90
V [Å^3]	5187.9(5)	2655.6(2)	4719.3(4)
Z	4	2	4
ρ [g·cm^{-3}]	1.335	1.264	1.298
F(000)	2224	1080	1968
μ [mm^{-1}]	0.933	0.871	0.895
T_{min} / T_{max}	0.5016 / 0.8051*	0.6731 / 0.7859*	0.71 / 0.82$
θ–range [°]	1.336 – 25.839	1.182 – 25.829	2.049 – 27.920
hkl–range	±21, ±18, ±24	–13 to 15, ±16, ±21	±18, ±31, –17 to 19
measured refl.	37636	31554	52409
unique refl. [R_{int}]	9830 [0.1421]	10012 [0.0258]	19470 [0.0434]
observed refl. ($I > 2\sigma(I)$)	5586	8477	14963
data / restr. / param.	9830 / 185 / 684	10012 / 336 / 715	19470 / 468 / 1152
goodness–of–fit (F^2)	1.074	1.060	1.059
$R1$, $wR2$ ($I > 2\sigma(I)$)	0.0703 / 0.1455	0.0386 / 0.1115	0.0538 / 0.1197
$R1$, $wR2$ (all data)	0.1475 / 0.1857	0.0484 / 0.1201	0.0831 / 0.1365
res. el. dens. [e·Å^{-3}]	–0.539 / 1.194	–0.479 / 0.874	–0.691 / 1.508

*) X-RED. $) SADABS.

Table S3. Crystal data and refinement details for **4**, **5**, and **6**.

**compound**	**4**	**5**	**6**
empirical formula	$C_{44.80}H_{66.40}N_6Ni_2O_{0.60}S$	$C_{41}H_{58}N_6Ni_2O_{0.50}S$	$C_{41}H_{57}N_6Ni_2O_{0.50}S$
moiety formula	$C_{40}H_{56}N_6Ni_2S$, 0.4(C_6H_{14}), 0.6(C_4H_8O)	$C_{39}H_{54}N_6Ni_2S$, 0.5(C_4H_8O)	$C_{39}H_{53}N_6Ni_2S$, 0.5(C_4H_8O)
formula weight	848.12	792.41	791.40
T [K]	133(2)	133(2)	133(2)
crystal size [mm^3]	0.370 x 0.140 x 0.130	0.440 x 0.200 x 0.160	0.300 x 0.100 x 0.070
crystal system	triclinic	monoclinic	monoclinic
space group	P–1 (No. 2)	$P2_1/c$ (No. 14)	$P2_1/c$ (No. 14)
a [Å]	12.1569(5)	17.2224(6)	17.2564(4)
b [Å]	13.2965(5)	13.9813(4)	14.0118(2)
c [Å]	14.2127(6)	17.3091(7)	17.2717(4)
α [°]	86.865(3)	90	90
β [°]	70.848(3)	112.278(3)	112.378(2)
γ [°]	85.265(3)	90	90
V [Å^3]	2161.91(16)	3856.8(2)	3861.68(15)
Z	2	4	4
ρ [g·cm^{-3}]	1.303	1.365	1.361
F(000)	908	1688	1684
μ [mm^{-1}]	0.959	1.070	1.068
T_{min} / T_{max}	0.4983 / 0.7187*	0.6153 / 0.8679*	0.5630 / 0.8699*
θ–range [°]	1.517 – 26.945	1.278 – 26.771	1.933 – 26.787
hkl–range	±15, ±16, –18 to 17	±21, ±17, ±21	±21, ±17, ±21
measured refl.	30771	28765	46739
unique refl. [R_{int}]	9177 [0.0313]	8161 [0.0550]	8190 [0.0410]
observed refl. ($I > 2\sigma(I)$)	7142	5913	6505
data / restr. / param.	9177 / 90 / 586	8161 / 7 / 503	8190 / 5 / 499
goodness–of–fit (F^2)	1.019	0.921	1.122
$R1$, $wR2$ ($I > 2\sigma(I)$)	0.0400 / 0.1005	0.0364 / 0.0678	0.0380 / 0.0861
$R1$, $wR2$ (all data)	0.0587 / 0.1117	0.0626 / 0.0733	0.0589 / 0.0983
res. el. dens. [e·Å^{-3}]	–0.800 / 1.066	–0.313 / 0.671	–0.381 / 1.211

*) X-RED.

G XES and XAS Data and DFT Calculations

Sample preparation for XAS and XES measurements. For XAS experiments, solid samples were diluted with boron nitride to achieve a 2% (w/w) concentration of nickel, then packed into 1 mm thick aluminum sample cells and sealed with 13 μm Kapton tape. For XES experiments, all samples were measured in the solid state. For Ni XES, the pure solids were ground to a fine powder and packed into 0.5 mm thick aluminum sample holders and sealed with 13 μm Kapton tape. For S XES, the pure solids were ground to a fine powder and packed into 0.5 mm thick aluminum sample holders and sealed with polypropylene.

Ni K-edge XAS. Ni K-edge X-ray absorption data were measured at SuperXAS beamline of Swiss Light Source (SLS, Switzerland) (in QEXAFS mode). Samples were measured as solids diluted in BN, sealed with Kapton tape, followed by freezing and storage under liquid nitrogen until measurement. Samples were measured at room temperature inside a plastic bag filled with helium under anaerobic and anhydrous condition. The SLS storage ring was operated at 2.4 GeV with a 400 mA. X-ray radiation was generated from a superbending magnet and monochromatized by a Si(111) double crystal monochromator (QEXAFS mode) with an energy resolution of 1.4×10^{-4} ($\Delta E/E$). [10] The X-ray beam was vertically collimated and then horizontally focused by a Pt-coated toroidal mirror to the sample position, resulting in a beam spot size of approximately 0.5×0.2 mm (horizontal × vertical) and a photon flux of around 1011 photons/s. High order harmonics (> 10 keV) was removed by harmonic rejection mirror. The Ni K-edge XAFS spectra were collected between 8130 eV and 9520 eV (kmax = 17.6) in transmission mode. The incident beam intensity (I_0) and the transmitted beam intensity (I_t) were monitored by ionization chambers filled with mixture of He and N_2. For each sample, consecutive 900 full XAFS scans were collected within 15 minutes (1 spectrum/sec) and merged to improve the data quality.

No noticeable change/trend in the XANES region was observed for the samples during the 15-minute data collection period, suggesting no sign of radiation damage.

The Ni K-edge QEXAFS scans were processed using the ProQEXAFS python package (version 2.42).[11] First, the raw QEXAFS datasets were extracted from the binary stream data files. Then, the splitting of the oscillations of the monochromator (1 Hz) was analyzed with the data sampling frequency (1 MHz) to get the unique encoder values per half oscillation of the monochromator for refactoring of the original spectra. With the monochromator encoder defined, the monochromator angle to energy was calibrated by aligning the energy of the first inflection in the first derivative in XANES of Ni foil, which was simultaneously measured using the same frequency (1 Hz) during the QEXAFS data collection of the Ni complexes, to 8333 eV. As the raw data was collected with angular resolution of 0.00005°, the number of unique energy points per spectrum is about 42000, which is significantly oversampling of the XAFS spectrum. Therefore, fast localized radial basis function interpolation was applied to reduce the density of data points in the XAFS spectra, with energy step size of 0.2 eV for the XANES region. Meanwhile, a Butterworth noise filtering (cut off frequency of 20 kHz) was used to remove the high frequency noise which originates from the data acquisition system. In the last step, the 900 scans for each sample were merged and exported for further analysis. In the next step, pre-edge background subtraction and post-edge normalization were performed using the xraylarch python package (version 0.9.74). [12] For the XANES region, a linear regression background (8287 - 8322 eV) was determined, and a cubic regression for post-edge

normalization was applied (8387 - 8837 eV). Energy calibrations of the spectra were carried out based on the XAFS spectra of Ni foil which were simultaneously measured during each scan.

Ni and S VtC XES measurements. The Ni and S valence-to-core X-Ray Emission Spectroscopy (VtC XES) data collection was done at the PINK tender X-ray beamline at BESSY II.[13] The PINK beamline is currently being operated in commissioning mode. A considerable gain in intensity of the photon beam was obtained by using a multilayer monochromator (≈ 80–100 eV band pass). The beam size on the sample was 30 µm × 500 µm (V×H). All spectra were collected using two in-house designed energy dispersive von Hamos spectrometers. The analyzers were set up in a vertical dispersion direction, taking advantage of the small vertical beam size to improve the energy resolution. The sample environment was fixed at 22 K in a cryo-chamber, using Helium as the exchange gas, at a pressure of 8 mbar. The entrance window was made from 1μm graphenic carbon and for the exit window, a cold window of 8 μm Kapton was used and the window to the For Ni Kβ XES measurements an atmospheric von Hamos spectrometer was used. A Si (444) 1 mm striped crystal with a bending radius of R = 247 mm dispersed incoming fluorescence radiation onto an Eiger detector with a 75 µm × 75 µm pixel size. The Eiger detector accepted fluorescent radiation reflected from the crystal analyzer under 69–77º Bragg's angles that corresponded to an energy window of 8120–8480 eV (spanning the Kβ mainline and valence-to-core regions). The spectrometer resolution was about 1 eV. The excitation energy was 9500 eV and incoming photon flux ≈ $8{\cdot}10^{12}$ ph/s using atmospheric von Hamos spectrometer. Ni Kβ XES damage scans and assessments were performed for all samples. In all cases, the data were collected with continuous sample motion. Samples were scanned at a rate of 100 µm/s, resulting in a total exposure time of 0.50 s/pass and the total exposure was 20 x 0.5 =10 s per sample, with a total measurement time of 2 h. None of the samples exhibited damage or changes in the Kβ emission spectra within the measurement scan periods. XES data were processed and the integrated intensity of the Kβ main line was set to 1.0. For the energy calibration procedure Ta, Ni and Tb foils were used. The energy points used for the energy calibration were: Ta: $L\alpha_1$ 8146.1, Ni Kβ: 8264.66, Tb $L\alpha_2$ 6238.0 and Tb Lα1: 6272.8 eV.[14] The Tb $L\alpha_{1,2}$ spectrum was collected from Si (333) reflection of the same crystal without any rearrangements of the photon beam or the spectrometer. The peak positions were defined by maximum of intensity. Energies was translated into Bragg angles and a fit with tangential function was applied.

The S-Kβ XES spectra were collected using a Si (111) crystal with a bending radius of R = 247 mm dispersed incoming fluorescence radiation onto a CCD detector with a 26 μm × 26 μm pixel size. The CCD detector accepted fluorescent radiation reflected from the crystal analyser under 52º–54º Bragg's angles that corresponded to an energy window of 2436–2500 eV. In the current configuration, the detector resolution was 0.06 eV/pix. The spectrometer resolution was about 0.5 eV. The excitation energy was 4000 eV and incoming photon flux was ≈ $3{\cdot}10^{13}$ ph/s. Measurements were done using zigzag mode, when samples were irradiated from spot to spot over the sample area (moment dose 1s at spot, 4 passes, total dose 4s). This was replicated three times at different sample areas. Total measurement time for each sample was ≈ 3 x 15 min = 45 min. None of the samples exhibited damage or changes in VtC XES spectra the measurement scan periods. XES data were processed and the integrated intensity of the Kβ main line was set to 1.0. Calibration of the energy scale was done with three reference samples: Na_2SO_3 and Na_2SO_4 powders, and Ni foil. Ni K$\alpha_{1,2}$ emission spectrum was collected at higher excitation energy of 9500 eV using Si (333) reflection of the same crystal.

The reference emission spectra were fitted with 5 and 4 Voigt profiles, and 2 asymmetric Lorentzian profiles respectively. The reference energies are presented in Table S4. For the energy calibration we converted energies of the tabulated peaks to Bragg angles and fit positions of the picked peaks with a tangential function.

Table S4. Reference energies (eV) used for the S-Kβ XES energy calibration.[15]

	Kβ'	K$\beta_{1,3}$	Kβ_{XX}	Kα_1	Kα_2
Na_2SO_3	2451.84	2465.83	2472.31		
Na_2SO_4	2452.91	2467.15			
Ni foil				7478.26	7461.04

Computational details. All XES spectra were calculated within the ORCA code utilizing previously published protocols.[16] These DFT calculations were performed with the ORCA electronic structure package 5.0.3.[17] Geometry optimization calculations were carried out at the B3LYP[18] level of theory, using def2- variants of Ahlrichs' all electron Gaussian triple-ζ valence polarized recontracted basis set (def2-TZVP)[19] on all atoms and the AutoAux basis option for ORCA.[20] The calculations employed the resolution of identity (RI-J) algorithm for the computation of the Coulomb terms and the 'chain of spheres exchange' (COSX) algorithm for the calculation of the exchange terms[21] and a tight self-consistent field (SCF) convergence threshold was chosen via the "TightSCF" keyword. Defgrid2 was used during the SCF iterations and for the final energy evaluation after SCF convergence. The conductor-like polarizable continuum model (CPCM) was used for charge compensation in all calculations of complexes carrying a net positive/negative charge.[22] Ni Kβ and S Kβ VtC XES were calculated using the features described above, with the exception that a scalar relativistic basis set (ZORA-def2-TZVP)[19] was employed. In the XES block, "CoreOrb" dictates the orbitals that will be the electron acceptors. All virtual orbitals were chosen as potential acceptor orbitals. For transition metal XES spectra, the metal 1s orbital is usually orbital 0, and is thus selected. OrbOp defines the operators of the electrons (α = 0, β = 1) that will be calculated, both of which were selected. CoreOrbSOC defines which core orbitals (α = 0, β = 1) are treated with spin-orbit coupling (SOC), both of which were selected to accommodate the OrbOp selection. A 2.0 eV full-width half-max Gaussian broadening and −35 eV energy shift was applied when plotting all calculated Ni and S XES spectra.

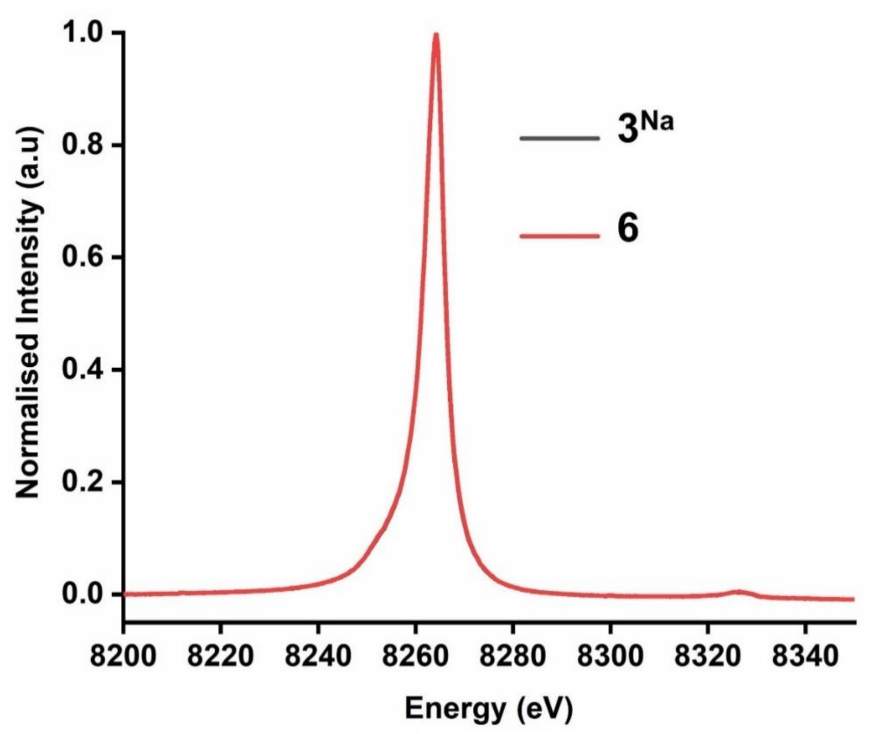

Figure S55. Experimental Ni K-*β* XES mainline spectra for complexes **3^{Na}** and **6**. The spectral data superimposes implicating no metal-based oxidation event.

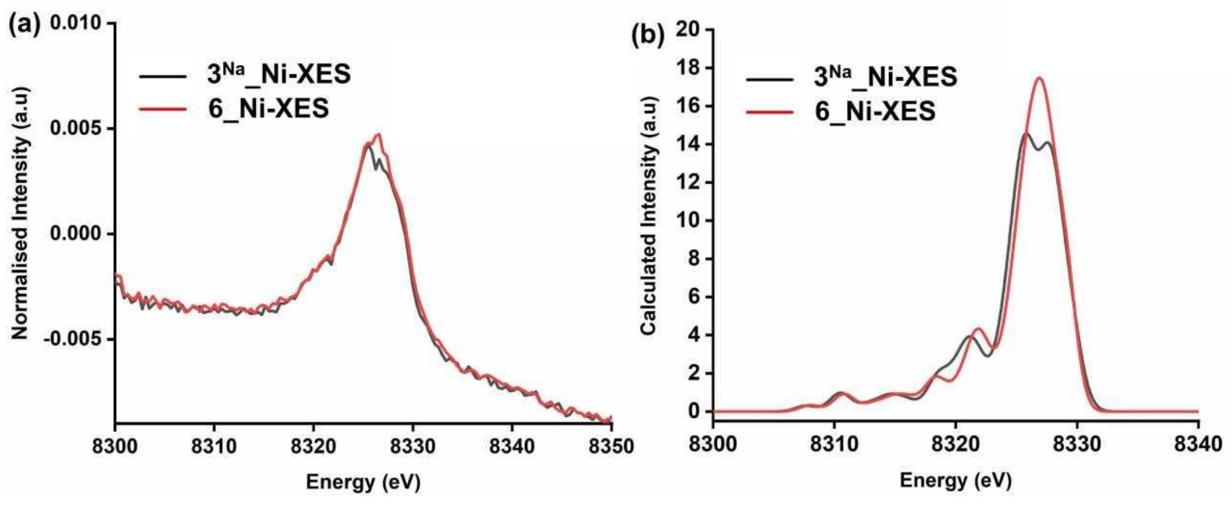

Figure S56. (a) Experimental (left) and (b) DFT calculated (right, B3LYP/def2-TZVP) Ni VtC K*β* XES spectra for complexes **3^{Na}** and **6**.

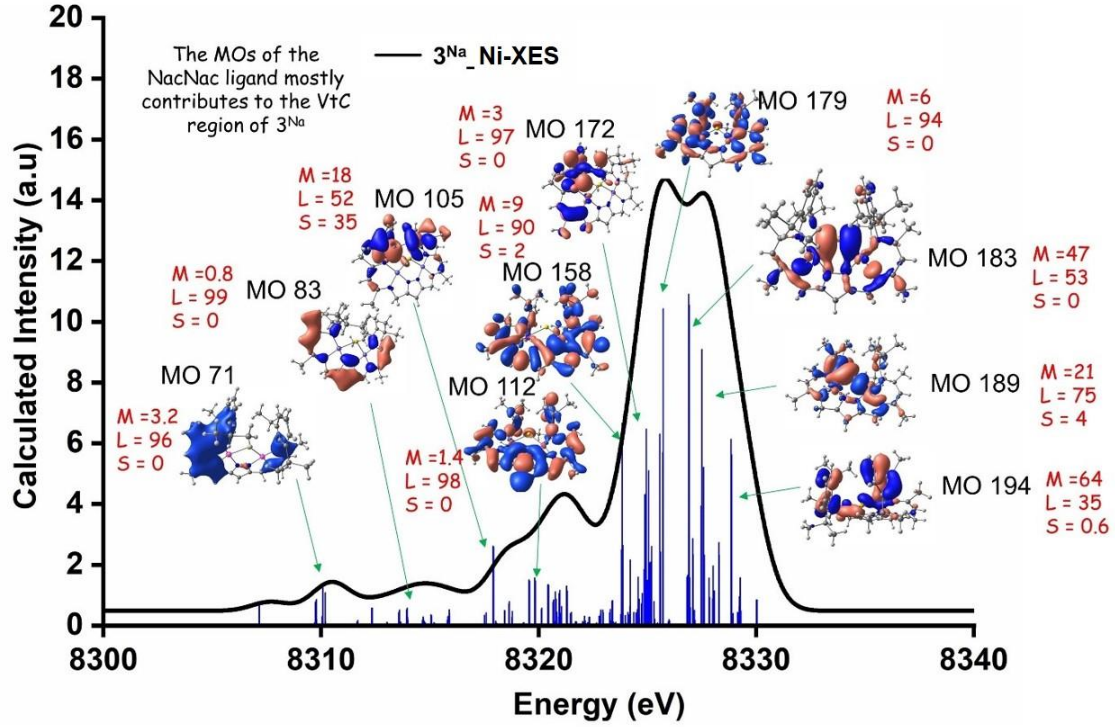

Figure S57. DFT calculated (B3LYP/def2-TZVP) Ni VtC Kβ XES spectrum for complex **3^{Na}** showing the molecular orbital contributions to the VtC transition.

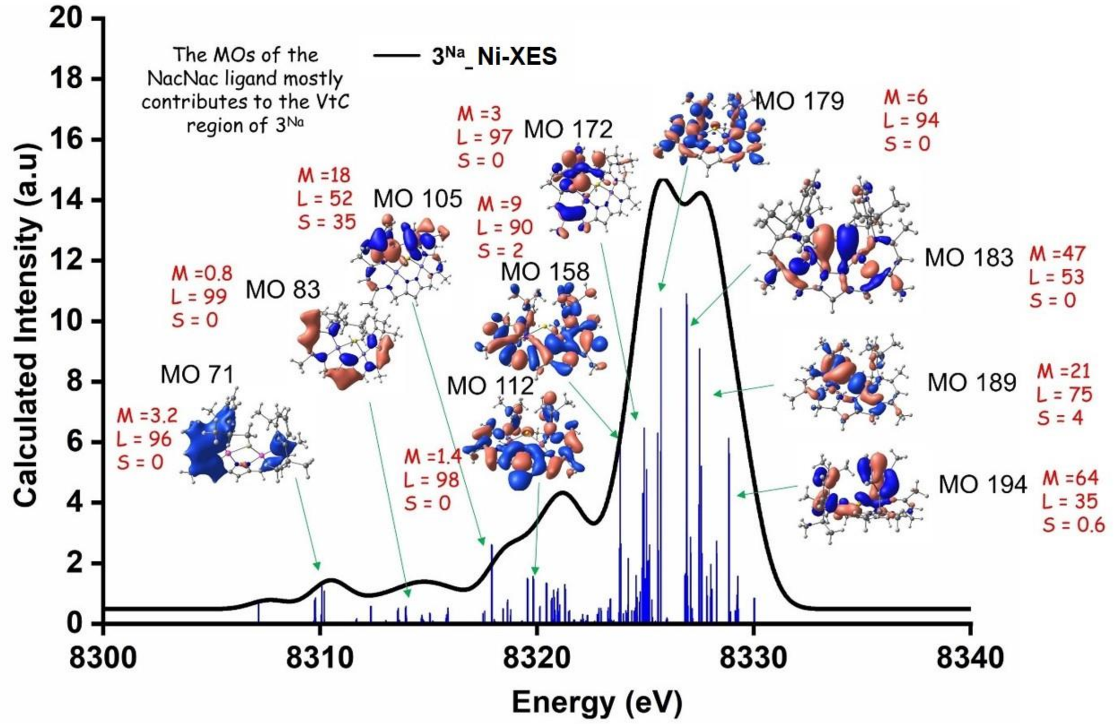

Figure S58. DFT calculated (B3LYP/def2-TZVP) Ni VtC Kβ XES spectrum for complex **6** showing the molecular orbital contributions to the VtC transition.

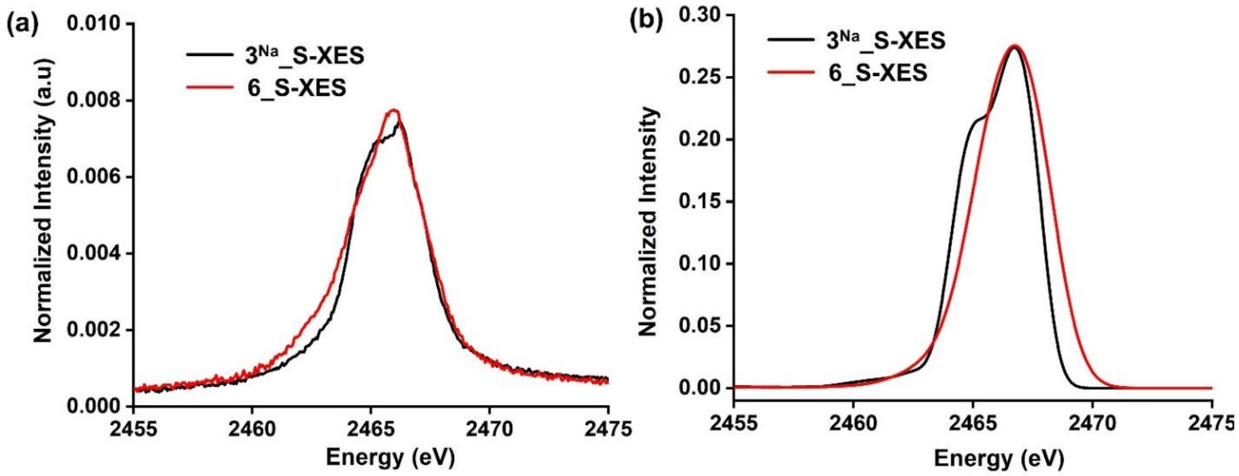

Figure S59. (a) Experimental (left) and (b) DFT calculated (right, B3LYP/def2-TZVP) S VtC Kβ XES spectra for complexes **3^{Na}** and **6**.

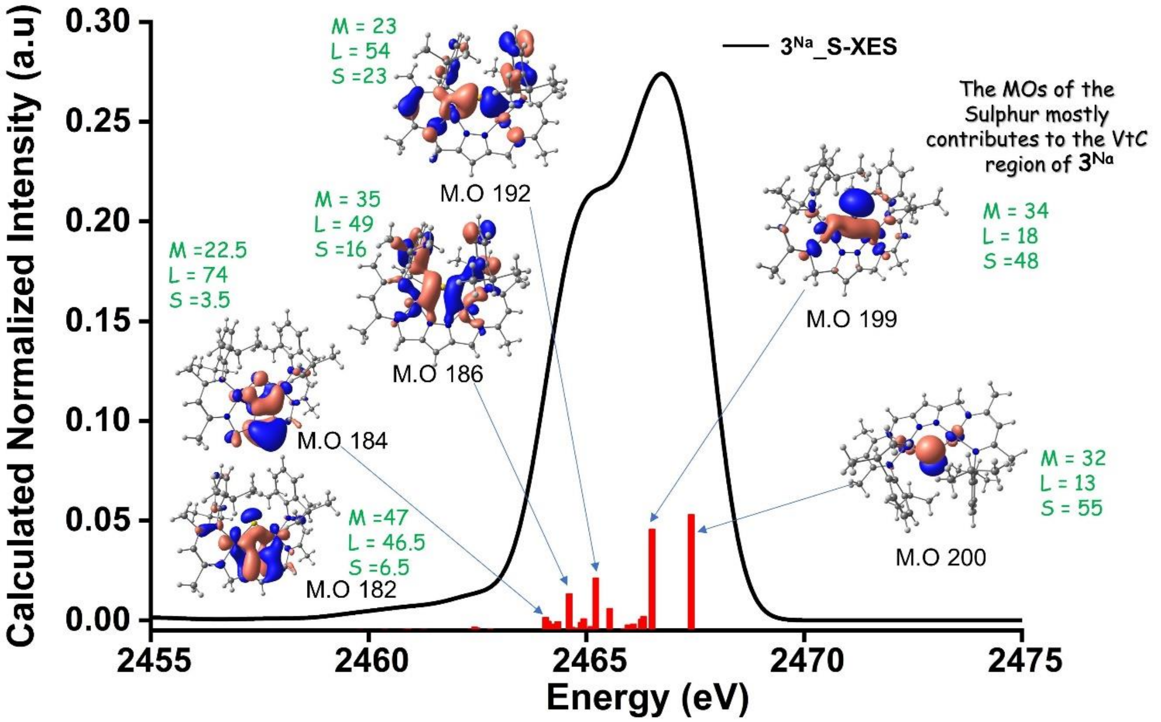

Figure S60. DFT calculated (B3LYP/def2-TZVP) S VtC Kβ XES spectrum for complex **3^{Na}** showing the molecular orbital contributions to the VtC transition.

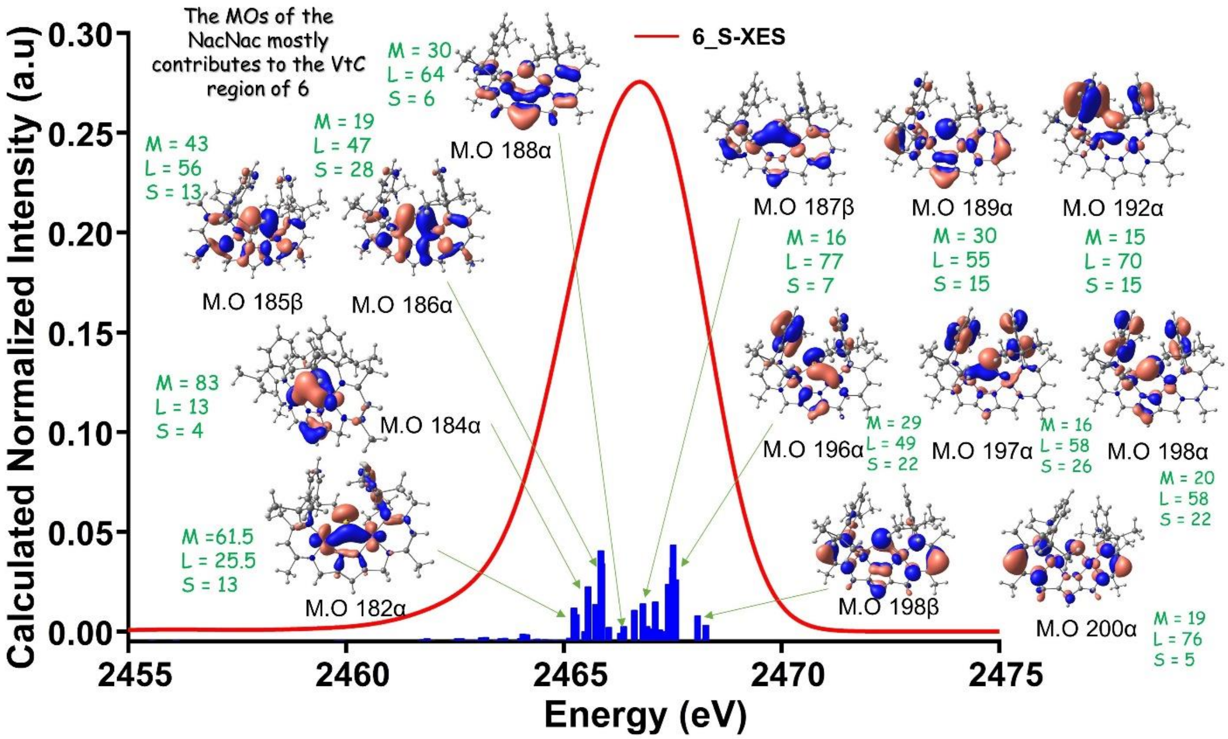

Figure S61. DFT calculated (B3LYP/def2-TZVP) S VtC K*β* XES spectrum for complex **6** showing the molecular orbital contributions to the VtC transition.

Table S5. Analyses of Löwdin charge (e) and Löwdin spin density for **3^{Na} and 6** with respect to Ni and S at the B3LYP-D3/def2-TZVP level.

Complex	Löwdin charge (e)			Löwdin spin density		
	Ni1	Ni2	S	Ni1	Ni2	S
**3^{Na}**	**-0.29**	**-0.29**	**0.19**	-	-	-
6	-0.22	-0.22	0.46	0.17	0.17	0.52

Löwdin charge analyses for electronic structure elucidation at the DFT level reveals that the charge on both Ni centers remains almost unchanged on going from **3^{Na}** to **6**, however the charge on S increases on going from **3^{Na}** to **6**, suggesting 1e$^-$ oxidation on S for complex **6**. The excess Löwdin spin density on S in complex **6** also implicates a sulfido based radical.

Table S6. DFT optimized coordinates for **3^{Na}**.

Ni	3.673975000000	16.426803000000	9.959151000000
Ni	0.234548000000	16.454293000000	11.096512000000
S	1.886185000000	15.148048000000	10.338792000000
N	2.638518000000	17.858680000000	10.412079000000
N	1.322773000000	17.858680000000	10.717439000000

N	5.007753000000	17.808041000000	9.845387000000
N	4.817535000000	15.030132000000	9.358230000000
N	-1.014163000000	17.875560000000	11.413377000000
N	-0.912722000000	15.059068000000	11.694592000000
C	2.041034000000	19.966226000000	10.771410000000
H	2.073649000000	20.913079000000	10.856513000000
C	3.090915000000	19.095718000000	10.444745000000
C	0.947376000000	19.141534000000	10.943264000000
C	4.549235000000	19.182528000000	10.115240000000
H	4.689574000000	19.753953000000	9.319599000000
H	5.052286000000	19.569289000000	10.874977000000
C	6.288152000000	17.629599000000	9.558490000000
C	6.814971000000	16.373270000000	9.228985000000
H	7.751573000000	16.333892000000	9.074444000000
C	6.112421000000	15.172403000000	9.102580000000
C	7.237395000000	18.823232000000	9.531505000000
H	8.152192000000	18.510115000000	9.369053000000
H	7.200947000000	19.290051000000	10.392706000000
H	6.970882000000	19.437218000000	8.815854000000
C	6.928277000000	14.012529000000	8.572815000000
H	7.878495000000	14.174357000000	8.749044000000
H	6.785213000000	13.928348000000	7.606555000000
H	6.648521000000	13.186463000000	9.017945000000
C	4.287830000000	13.706284000000	9.075595000000
C	3.842142000000	13.349400000000	7.797342000000
C	3.445627000000	12.040020000000	7.565836000000
H	3.160461000000	11.785764000000	6.696340000000
C	3.458773000000	11.082702000000	8.592699000000
H	3.203652000000	10.183644000000	8.417380000000
C	3.847737000000	11.466112000000	9.858169000000
H	3.831673000000	10.832642000000	10.566222000000
C	4.262084000000	12.761023000000	10.110979000000
C	3.758005000000	14.359768000000	6.669639000000
H	4.155111000000	15.219497000000	6.990083000000
C	2.317007000000	14.615374000000	6.291844000000
H	2.266603000000	15.397047000000	5.703819000000
H	1.791726000000	14.782483000000	7.103052000000
H	1.956908000000	13.831748000000	5.826375000000
C	4.537161000000	13.904017000000	5.433994000000
H	4.546401000000	14.625116000000	4.770042000000
H	4.106017000000	13.112216000000	5.049851000000
H	5.456622000000	13.683930000000	5.690242000000
C	4.688188000000	13.146844000000	11.536941000000
H	4.699935000000	14.144745000000	11.582731000000
C	3.681336000000	12.657334000000	12.572326000000
H	3.921968000000	13.011565000000	13.453511000000
H	3.689525000000	11.678482000000	12.599112000000
H	2.784796000000	12.970451000000	12.328237000000
C	6.089753000000	12.664568000000	11.849403000000
H	6.301842000000	12.859841000000	12.785979000000
H	6.730764000000	13.123116000000	11.266832000000
H	6.145014000000	11.697411000000	11.697291000000
C	-0.490555000000	19.262103000000	11.274189000000
H	-0.971217000000	19.740425000000	10.553184000000

H	-0.608961000000	19.761621000000	12.119711000000
C	-2.328684000000	17.709175000000	11.615057000000
C	-2.889570000000	16.469725000000	11.845142000000
H	-3.835307000000	16.443731000000	11.932617000000
C	-2.214833000000	15.247156000000	11.965866000000
C	-3.231801000000	18.924510000000	11.687491000000
H	-4.160730000000	18.634084000000	11.808542000000
H	-3.156377000000	19.438930000000	10.857450000000
H	-2.965165000000	19.486024000000	12.445623000000
C	-3.047614000000	14.118630000000	12.525457000000
H	-3.993722000000	14.284316000000	12.333818000000
H	-2.914379000000	14.063988000000	13.494656000000
H	-2.773207000000	13.273634000000	12.110280000000
C	-0.418589000000	13.742455000000	11.982909000000
C	-0.412861000000	12.758612000000	10.992974000000
C	-0.015377000000	11.466112000000	11.331001000000
H	-0.026114000000	10.783884000000	10.669746000000
C	0.390631000000	11.162278000000	12.607833000000
H	0.644707000000	10.274070000000	12.825406000000
C	0.433213000000	12.148532000000	13.576465000000
H	0.741956000000	11.937271000000	14.449511000000
C	0.027832000000	13.450678000000	13.285307000000
C	-0.881791000000	13.050389000000	9.595417000000
H	-0.907254000000	14.045348000000	9.488655000000
C	0.108019000000	12.498183000000	8.548670000000
H	-0.138011000000	12.828325000000	7.659944000000
H	0.075638000000	11.518825000000	8.554721000000
H	1.015633000000	12.796205000000	8.768615000000
C	-2.286782000000	12.522296000000	9.314202000000
H	-2.534768000000	12.730808000000	8.389258000000
H	-2.923182000000	12.945614000000	9.926811000000
H	-2.302006000000	11.551451000000	9.445663000000
C	0.106312000000	14.504451000000	14.378923000000
H	-0.494788000000	15.253666000000	14.099085000000
C	1.515892000000	15.088005000000	14.469821000000
H	1.536801000000	15.787015000000	15.157947000000
H	1.766194000000	15.475441000000	13.605097000000
H	2.149006000000	14.379083000000	14.706255000000
C	-0.388885000000	14.034232000000	15.732451000000
H	-0.339317000000	14.774381000000	16.373651000000
H	0.169824000000	13.293190000000	16.045182000000
H	-1.318050000000	13.733195000000	15.653171000000

Table S7. DFT optimized coordinates for **6.**

Ni	3.485443000000	7.002817000000	5.040450000000
Ni	3.195237000000	8.974838000000	8.163261000000
S	2.060754000000	7.622559000000	6.706066000000
N	4.777533000000	7.804713000000	6.050297000000
N	4.678627000000	8.497456000000	7.208834000000
N	4.980538000000	6.542670000000	3.968316000000
N	2.210850000000	6.151180000000	3.921042000000
N	4.491021000000	9.769728000000	9.291134000000
N	1.718017000000	9.653570000000	9.134139000000

C	6.839337000000	8.429779000000	6.649210000000
H	7.780387000000	8.559472000000	6.655774000000
C	6.077002000000	7.746284000000	5.707719000000
C	5.901695000000	8.882781000000	7.584951000000
C	6.316335000000	6.977596000000	4.466292000000
H	6.887889000000	6.191030000000	4.652291000000
H	6.768221000000	7.543981000000	3.791597000000
C	4.963988000000	5.856932000000	2.837888000000
C	3.783541000000	5.395944000000	2.257662000000
H	3.866627000000	4.933765000000	1.431609000000
C	2.498558000000	5.541667000000	2.763304000000
C	6.259618000000	5.523452000000	2.135803000000
H	6.069668000000	4.988929000000	1.336965000000
H	6.837083000000	5.013884000000	2.741072000000
H	6.711147000000	6.353132000000	1.874102000000
C	1.389961000000	4.962980000000	1.902946000000
H	1.770371000000	4.319992000000	1.269072000000
H	0.948797000000	5.686143000000	1.409473000000
H	0.735206000000	4.510903000000	2.474500000000
C	0.826186000000	6.105782000000	4.325109000000
C	-0.062109000000	7.109587000000	3.883191000000
C	-1.395490000000	7.025517000000	4.292208000000
H	-2.009350000000	7.696682000000	4.019519000000
C	-1.841663000000	5.980236000000	5.088363000000
H	-2.752992000000	5.939714000000	5.353466000000
C	-0.961285000000	4.999410000000	5.494186000000
H	-1.277328000000	4.283127000000	6.032872000000
C	0.383628000000	5.037242000000	5.129249000000
C	0.386564000000	8.252950000000	2.984981000000
H	1.349521000000	8.104593000000	2.759119000000
C	0.277344000000	9.579868000000	3.702080000000
H	0.545627000000	10.301545000000	3.095229000000
H	0.865150000000	9.576953000000	4.485889000000
H	-0.648946000000	9.721205000000	3.988136000000
C	-0.394696000000	8.323009000000	1.675359000000
H	-0.023768000000	9.032595000000	1.110704000000
H	-1.336674000000	8.517535000000	1.867075000000
H	-0.327924000000	7.464450000000	1.207887000000
C	1.331403000000	3.924705000000	5.543217000000
H	2.261563000000	4.236566000000	5.350112000000
C	1.255421000000	3.619248000000	7.022452000000
H	1.912092000000	2.927485000000	7.248330000000
H	0.355530000000	3.299176000000	7.244769000000
H	1.448041000000	4.431988000000	7.534994000000
C	1.104223000000	2.655236000000	4.744986000000
H	1.745288000000	1.971068000000	5.028887000000
H	1.228462000000	2.843204000000	3.790607000000
H	0.191743000000	2.332068000000	4.897414000000
C	5.903347000000	9.621623000000	8.865986000000
H	6.320827000000	10.511554000000	8.747928000000
H	6.416423000000	9.121766000000	9.549353000000
C	4.267459000000	10.407685000000	10.433061000000
C	2.984368000000	10.646866000000	10.919857000000
H	2.917203000000	11.041957000000	11.781174000000

C	1.791743000000	10.361166000000	10.262331000000
C	5.431093000000	10.937611000000	11.245666000000
H	5.096564000000	11.323300000000	12.081988000000
H	5.900881000000	11.627286000000	10.732278000000
H	6.048564000000	10.203785000000	11.448434000000
C	0.541245000000	10.967036000000	10.877853000000
H	0.681542000000	11.099489000000	11.839452000000
H	-0.217647000000	10.363450000000	10.739097000000
H	0.353698000000	11.830947000000	10.454334000000
C	0.413507000000	9.564455000000	8.522449000000
C	-0.030330000000	10.625148000000	7.711441000000
C	-1.285931000000	10.517257000000	7.115084000000
H	-1.600326000000	11.213181000000	6.550812000000
C	-2.078632000000	9.404720000000	7.337081000000
H	-2.936178000000	9.347622000000	6.931465000000
C	-1.635640000000	8.381859000000	8.138826000000
H	-2.194160000000	7.626511000000	8.277933000000
C	-0.376541000000	8.425295000000	8.757702000000
C	0.824668000000	11.865192000000	7.476029000000
H	1.497121000000	11.911711000000	8.215023000000
C	1.590206000000	11.783924000000	6.163212000000
H	2.164744000000	12.573054000000	6.069446000000
H	0.957359000000	11.750660000000	5.416040000000
H	2.144841000000	10.975275000000	6.157478000000
C	0.007565000000	13.158481000000	7.517553000000
H	0.612466000000	13.927967000000	7.474480000000
H	-0.506684000000	13.192894000000	8.350968000000
H	-0.605643000000	13.181082000000	6.753740000000
C	0.118845000000	7.280531000000	9.632115000000
H	1.100482000000	7.189945000000	9.466751000000
C	-0.519933000000	5.938201000000	9.280753000000
H	-0.053766000000	5.220516000000	9.758302000000
H	-0.452437000000	5.786691000000	8.315529000000
H	-1.463835000000	5.946762000000	9.545376000000
C	-0.062838000000	7.535546000000	11.125404000000
H	0.213978000000	6.741245000000	11.628427000000
H	-1.004775000000	7.729091000000	11.312409000000
H	0.487691000000	8.300688000000	11.395410000000

H References

[1] Barbosa, J.; Barròn, D.; Bosch, E.; Rosés, M. Resolution of acid strength in tetrahydrofuran of substituted benzoic acids *Anal. Chim. Acta* **1992**, *265*, 157-165. DOI:10.1016/0003-2670(92)85165-3

[2] Barbosa, J.; Barròn, D. Acid–base behaviour of substituted phenolic substances and resolution of acid strength in tetrahydrofuran *Anal. Chim. Acta* **2000**, *403*, 343. DOI: 10.1016/S0003-2670(99)00635-2

[3] Garrido, G.; Kort, E.; Rafols, C.; Bosch, E.; Rodima, T.; Leito, I.; Rosés, M. Acid-Base Equilibria in Nonpolar Media. Absolute pKa Scale of Bases in Tetrahydrofuran, *J. Org. Chem.* **2006**, *71*, 9062-9067. DOI: 10.1021/jo061432g

[4] Saame, J.; Rodima, T.; Tshepelevitsh, S.; Kütt, A.; Kaljurand, I.; Haljasorg, T.; Koppel, I. A.; Leito, I. Experimental Basicities of Superbasic Phosphonium Ylides and Phosphazenes *J. Org. Chem*. **2016**, *81*, 7349−7361. DOI :10.1021/acs.joc.6b00872

[5] Quist, D. A.; Ehudin, M. A.; Schaefer, A. W.; Schneider, G. L.; Solomon, E. I.; Karlin, K. D. Ligand Identity-Induced Generation of Enhanced Oxidative Hydrogen Atom Transfer Reactivity for a $Cu^{II}_2(O_2^{\bullet-})$ Complex Drivenby Formation of a $Cu^{II}_2(-OOH)$ Compound with a Strong O−H Bond *J. Am. Chem. Soc.* **2019**, *141*, 12682–12696. DOI: 10.1021/jacs.9b05277

[6] Wise, C. F.; Agarwal, R. G.; Mayer, J. M. Determining Proton-Coupled Standard Potentials and X−H Bond Dissociation Free Energies in Nonaqueous Solvents Using Open-Circuit Potential Measurements. *J. Am. Chem. Soc*. **2020**, *142*, 10681−10691. DOI: 10.1021/jacs.0c01032

[7] (a) Sheldrick, G. M. SHELXT- Integrated space-group and crystal-structure determination, *Acta Cryst*. **2015**, *A71*, 3-8; DOI: 10.1107/S2053273314026370; (b) Sheldrick, G. M. Crystal Structure Refinement with SHELXL, *Acta Cryst.* **2015**, *C71*, 3-8. DOI: 10.1107/S2053229614024218S

[8] *X-RED*; STOE & CIE GmbH, Darmstadt, Germany, **2002**.

[9] *SADABS*; BRUKER AXS GmbH, Karlsruhe, Germany, **2016**.

[10] Müller, O.; Nachtegaal, M.; Just, J.; Lützenkirchen-Hecht, D.; Frahm, R. Quick EXAFS Setup at the SuperXAS Beamline for in Situ X-Ray Absorption Spectroscopy with 10 Ms Time Resolution. *J. Synchrotron Rad.* **2016**, *23*, 260–266. https://doi.org/10.1107/S1600577515018007

[11] Clark, A. H.; Imbao, J.; Frahm, R.; Nachtegaal, M. ProQEXAFS : A Highly Optimized Parallelized Rapid Processing Software for QEXAFS Data. *J. Synchrotron Rad.* **2020**, *27*, 551–557. https://doi.org/10.1107/S1600577519017053

[12] Newville, M. Larch: An Analysis Package for XAFS and Related Spectroscopies. *J. Phys. Conf. Ser.* **2013**, *430*, 012007. DOI: 10.1088/1742-6596/430/1/012007

[13] Peredkov, S.; Pereira, N.; Grötzsch, D.; Hendel, S.; Wallacher, D.; DeBeer, S. PINK: a tender X-ray beamline for X-ray emission spectroscopy *J. Synchrotron Rad.* **2024**, *31*, 622-634. DOI: 10.1107/S1600577524002200

[14] Thompson, A., Vaughan, D., Laboratory, C. f. X.-r. O. L. B. N., optics, C. f. X.-r., source, a. l. & Source, L. B. N. L. A. L. **2009**; Lawrence Berkeley National Laboratory, University of California. URL: https://books.google.de/books?id=XAlzQEACAAJ

[15] a) Mori, R. A.; Paris, E.; Giuli, G.; Eeckhout, S. G.; Kavčič, M.; Žitnik, M.; Bučar, K.; Pettersson, L. G. M.; Glatzel, P. Sulfur-Metal Orbital Hybridization in Sulfur-Bearing Compounds Studied by X-ray Emission Spectroscopy *Inorg. Chem.* **2010,** *49*, 6468-6473. DOI: 10.1021/ic100304z; b) Hölzer, G.; Fritsch, M.; Deutsch, M.; Härtwig, J.; Förster, E. Ka1,2 and Kb1,3 X-ray emission lines of the 3d transition metals *Phys. Rev. A* **1997,** *56*, 4554-4568. DOI: 10.1103/PhysRevA.56.4554; c) Liu, Y.; Chatterjee, S.; Cutsail III, G. E.; Peredkov, S.; Gupta, S. K.; Dechert, S.; DeBeer, S.; Meyer, F. A Cu_4S Cluster in '0-Hole' and '1-Hole' States: Geometric and Electronic Structure Variations for the Active Cu_Z* site of N_2O Reductase. *J. Am. Chem. Soc.* **2023**, *145*, 18477−18486, DOI: 10.1021/jacs.3c04893

[16] (a) DeBeer, S., Petrenko, T.; Neese, F. Prediction of Iron K-Edge Absorption Spectra Using Time-Dependent Density FunctionalTheory *J. Phys. Chem. A* **2008,** *112*, 12936-12943. DOI: 10.1021/jp803174m; (b) Lee, N.; Bergmann, U.; Petrenko, T.; Neese, F.; DeBeer, S. Probing Valence Orbital Composition with Iron KβX-ray Emission Spectroscopy *J. Am. Chem. Soc.* **2010,** *132*, 9715-9727. DOI: 10.1021/ja101281e

[17] (a) Neese, F. The ORCA program system, *WIREs Comput. Mol. Sci.* **2012,** *2*, 73-78. DOI: 10.1002/wcms.81; (b) Neese, F. Software update: the ORCA program system, version 4.0 *WIREs Comput. Mol. Sci.* **2017**, *8*, e1327. DOI: 10.1002/wcms.1327

[18] (a) Lee, C.; Yang, W.; Robert G. Parr R. G. Development of the Colle-Salvetti correlation-energy formula into a functional of the electron density *Phys. Rev. B* **1988,** *37*, 785-789. DOI: 10.1103/physrevb.37.785; (b) Becke, A. D. Density-functional thermochemistry. III. The role of the exact exchange *J. Chem. Phys.* **1993**, *98*, 5648-5652. DOI: 10.1063/1.464913

[19] Weigend, F.; Ahlrichs, R. Balanced basis sets of split valence, triple zeta valence and quadruple zeta valence quality for H to Rn: Design and assessment of accuracy *Chem. Phys.* **2005**, *7*, 3297-3305. DOI: 10.1039/b508541a

[20] Stoychev, G. L.; Auer A. A.; Neese, F. J. Automatic generation of auxiliary basis set *Chem. Theory Comput.* **2017**, *13*, 554-562. DOI: 10.1021/acs.jctc.6b01041

[21] Neese, F.; Wennmohs, F., Hansen, A., Becker, U.; Efficient, approximate and parallel Hartree–Fock and hybrid DFT calculations. A 'chain-of-spheres' algorithm for the Hartree–Fock exchange *Chem. Phys.* **2009**, *356*, 98-109. DOI: 10.1016/j.chemphys.2008.10.036

[22] Klamt, A.; Schüürmann, G. COSMO: a new approach to dielectric screening in solvents with explicit expressions for the screening energy and its gradient *J. Chem. Soc., Perkin Trans. 2* **1993**, *5*, 799-805. DOI: 10.1039/p29930000799
